# Synthesis of cyclic β-1,6-oligosaccharides from glucosamine monomers by electrochemical polyglycosylation

**DOI:** 10.3762/bjoc.20.124

**Published:** 2024-06-26

**Authors:** Md Azadur Rahman, Hirofumi Endo, Takashi Yamamoto, Shoma Okushiba, Norihiko Sasaki, Toshiki Nokami

**Affiliations:** 1 Department of Chemistry and Biotechnology, Tottori University, 4-101 Koyamacho-minami, Tottori city, 680-8552 Tottori, Japan; 2 Center for Research on Green Sustainable Chemistry, Faculty of Engineering, Tottori University, 4-101 Koyamacho-minami, Tottori city, 680-8552 Tottori, Japan

**Keywords:** cyclic oligosaccharide, electrochemical glycosylation, glucosamine, polyglycosylation

## Abstract

The synthesis of protected precursors of cyclic β-1,6-oligoglucosamines from thioglycosides as monomers is performed by electrochemical polyglycosylation. The monomer with a 2,3-oxazolidinone protecting group afforded the cyclic disaccharide exclusively. Cyclic oligosaccharides up to the trisaccharide were obtained using the monomer with a 2-azido-2-deoxy group.

## Introduction

**Scheme 1:**
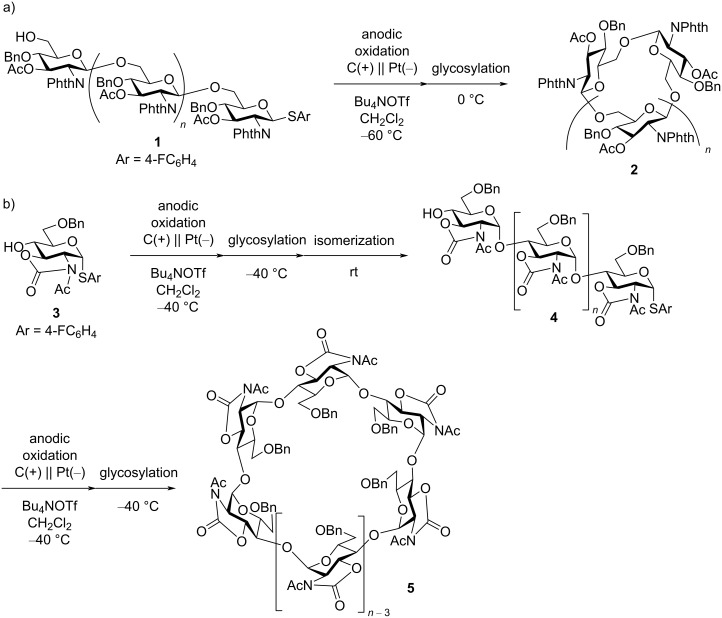
Preparation of cyclic oligoglucosamines a) via intramolecular glycosylation and b) via polyglycosylation and intramolecular glycosylation.

Electrochemical polymerization of organic molecules is an important strategy for the preparation of functional materials, such as conducting polymers [1–5]. Electrochemical reactions can be controlled by electric potential or current, electrodes, and electrolytes, which are not available in conventional chemical reactions. Therefore, electrochemical polymerizations can be utilized for selective synthesis. Cyclic oligosaccharides are an important class of host molecules, and some natural cyclic oligosaccharides are produced by enzymatic processes. However, the corresponding chemical syntheses are still primitive [6–10]. Thus, chemical glycosylation has to be improved to be able to synthesize complex oligosaccharides, including cyclic oligosaccharides. In this context, electrochemical glycosylation is an important alternative to conventional chemical glycosylations because the precise control of reaction time and rate is possible under electrochemical conditions [11–13]. We have been interested in the preparation of cyclic oligosaccharides under electrochemical conditions and electrochemical conversion of linear oligosaccharides of glucosamine into the corresponding cyclic oligosaccharides by intramolecular glycosylation (Scheme 1a) [14]. One-pot two-step synthesis via electrochemical polyglycosylation and intramolecular glycosylation has also been achieved in order to synthesize unnatural cyclic oligosaccharides of glucosamine (Scheme 1b) [15]. Here, we report the direct synthesis of cyclic oligoglucosamines via electrochemical polymerization of thioglycoside monomers that are derived from glucosamine hydrochloride.

## Results and Discussion

### Electrochemical polyglycosylation of 2-deoxy-2-phthalimido-substituted thioglycoside monomers

**Scheme 2:**
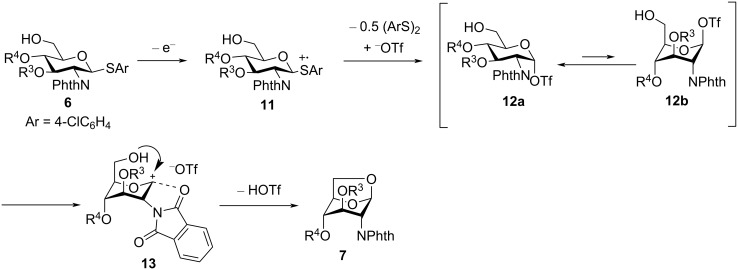
Proposed reaction mechanism of the formation of 1,6-anhydrosugar **7**.

We initiated our research with the electrochemical polyglycosylation of monomers **6** with a 2-deoxy-2-phthalimido (PhthN) group (Table 1). The influence of the anomeric leaving group was not investigated in this study. However, the *p*-ClC_6_H_4_S (ArS) group was used to avoid the exchange of the anomeric leaving group [16]. The monomer **6a** (R^3^ = R^4^ = Bz) was completely consumed with a slight excess of total charge (*Q* = 1.05 F/mol). However, 1,6-anhydrosugar **7a** (R^3^ = R^4^ = Bz) was formed as a major product, together with cyclic disaccharide **8a** (R^3^ = R^4^ = Bz) (Table 1, entry 1). The monosaccharide **6b** (R^3^ = Ac, R^4^ = Bn) was also completely consumed under the same reaction conditions. However, the yield of 1,6-anhydrosugar **7b** (R^3^ = Ac, R^4^ = Bn) was lower than that of **7a** (Table 1, entry 2). Because no linear oligosaccharides were obtained, we reduced the amount of total charge from 1.05 to 0.525 F/mol (Table 1, entry 3). Linear disaccharide **9b** (R^3^ = Ac, R^4^ = Bn) and trisaccharide **10b** (R^3^ = Ac, R^4^ = Bn) were obtained in 13% and 6% yield, respectively. The protecting group R^3^ of 3-OH was changed from an acetyl to a benzyl group. However, conversion and yield of linear oligosaccharides **9c** and **10c** decreased, and the corresponding cyclic disaccharide **8c** was not obtained at all (Table 1, entry 4). The reasons for the lower conversion and yield are unclear. However, the lower yield may stem from the lower stability of glycosylation intermediates with a benzyl protecting group at C-3. In all cases, the major product was 1,6-anhydrosugar **7**, which was the intramolecular glycosylation product of monomer **6**. The proposed mechanism is shown in Scheme 2. Anodic oxidation of thioglycoside **6** generated radical cation **11**, which was converted to glycosyl triflate **12**. 1,6-Anhydrosugar **7** was produced via ^4^C_1_-to-^1^C_4_ conformational change of the pyran ring to generate cation intermediate **13**. Therefore, prevention of the conformational change might be necessary to synthesize larger cyclic oligosaccharides.

**Table 1:** Electrochemical polyglycosylation of monomers **6** with a 2-PhthN group.

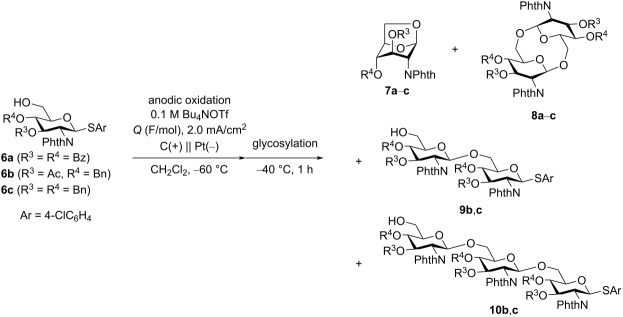								

entry	R^3^	R^4^	total charge *Q* (F/mol)	conversion^a^	yield of **7**	yield of oligosaccharide		

						**8**	**9**	**10**

1	Bz	Bz	1.05	>99%	**7a** (73%)	**8a** (3%)	—	—
2	Ac	Bn	1.05	>99%	**7b** (28%)	**8b** (6%)	—	—
3	Ac	Bn	0.525	67%	**7b** (25%)	**8b** (7%)	**9b** (13%)	**10b** (6%)
4	Bn	Bn	0.525	59%	**7c** (25%)	—	**9c** (4%)	**10c** (2%)

^a^Based on recovered starting material **6a**–**c**.

### Electrochemical polyglycosylation of 2,3-oxazolidinone-substituted thioglycoside monomer

**Scheme 3:**
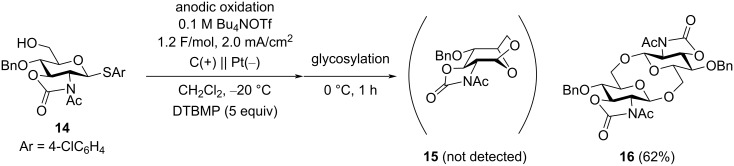
Electrochemical polyglycosylation of monomer **14** with a 2,3-oxazolidinone protecting group.

To avoid formation of 1,6-anhydrosugar, we introduced an *N*-acetyl-2,3-oxazolidinone protecting group to the thioglycoside monomer **14** (Scheme 3) [17–18]. The electrochemical polyglycosylation of **14** was carried out in the presence of 2,6-di-*tert*-butyl-4-methylpyridine (DTBMP) to ensure the formation of a β-glycosidic bonds [19]. Although we could suppress formation of 1,6-anhydrosugar **15**, cyclic disaccharide **16** was obtained as an exclusive product. The optimized structure of **15** calculated by DFT (B3LYP/6-31G(d)) suggested that the pyran ring preferred the boat conformation because the chair conformation of the pyran ring was controlled by the introduction of the 2,3-oxazolidinone protecting group (see DFT calculations in Supporting Information File 1). Therefore, it was proven that the 2,3-oxazolidinone protecting group was powerful enough to prevent intramolecular glycosylation of monomer **14**. However, it did not prevent intramolecular glycosylation of the linear disaccharide and promote the formation of larger cyclic oligosaccharides.

### Electrochemical polyglycosylation of 2-azido-2-deoxy-substituted thioglycoside monomers

**Scheme 4:**
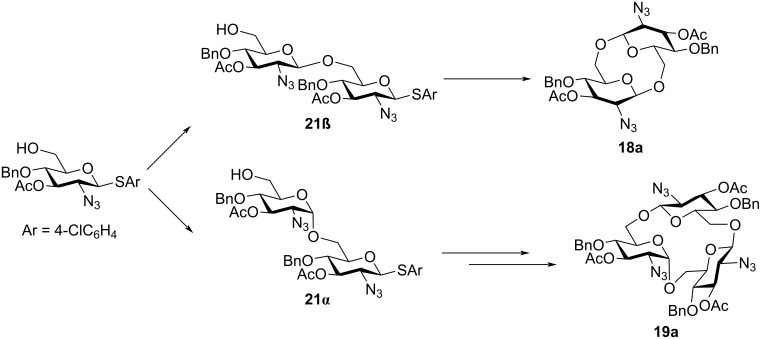
Proposed reaction mechanism of the formation of cyclic trisaccharide **19a**.

Based on the results shown in Table 1 and Scheme 3, we changed the substituent in position C-2 of the thioglycoside monomer from PhthN to azide, which has no neighboring group effect. Although glycosyl donors with an N_3_ group in position C-2 have been used for α-selective glycosylation [20–21], we have already found that β-selective glycosylation proceeds using a glycosyl donor with an N_3_ group under electrochemical conditions [22]. The results of the electrochemical polyglycosylation using the thioglycoside monomer **17** with an N_3_ group are summarized in Table 2. Cyclic trisaccharide **19a** was obtained together with cyclic disaccharide **18a**, along with trace amount of linear and cyclic tetrasaccharides by introduction of an N_3_ group (Table 2, entry 1). Cyclic disaccharide **18b** and linear trisaccharide **20b** were produced with monomer **17b** with a 3,4-di-*O*-benzyl group (Table 2, entry 2). Although the 3-hydroxy protecting group R^3^ also affected the product distribution, formation of the corresponding 1,6-anhydrosugars was not observed in both cases. NMR data suggested that cyclic trisaccharide **19a** contained one α-glycosidic bond and two β-glycosidic bonds. Based on these results, we assumed that the formation of the α-glycosidic bond was crucial for producing the cyclic trisaccharide **19a** (Scheme 4). Moreover, the α-glycosidic bond might have formed in the first step, and linear disaccharide **21α**, which did not afford the cyclic disaccharide, should have been produced as an intermediate of **19a**.

**Table 2:** Electrochemical polyglycosylation of monomers **17** with a 2-azido group.

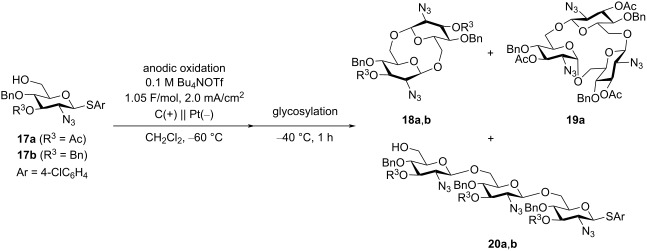					

entry	R^3^	conversion	yield of oligosaccharide		

			**18**	**19**	**20**

1	Ac	>99%	**18a** (49%)	**19a** (16%)	—
2	Bn	73%	**18b** (14%)	—	**20b** (13%)

**Scheme 5:**
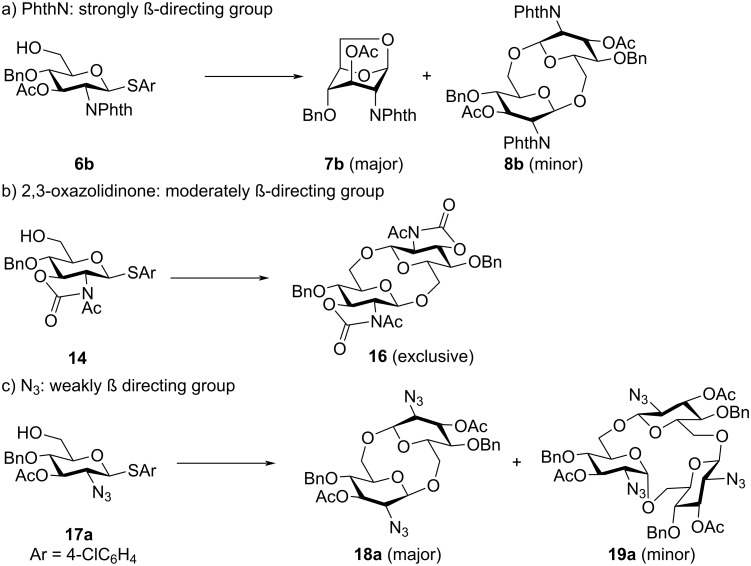
Influence of the functional group in position C-2 on the formation of the cyclic product.

The influence of the functional group in position C-2 on the formation of the cyclic products is summarized in Scheme 5. Notably, the C-2 position is the most influential because it is the closest to the anomeric carbon atom, which is the reaction site of the glycosylation. The PhthN group is known to be a strongly β-directing group, and 1,6-anhydrosugar **7b** was obtained as a major product (Scheme 5a). In this case, the competition between intramolecular glycosylation and intermolecular glycosylation (polyglycosylation) occurred, and intramolecular glycosylation was dominant because of the strong directing effect of the 2-PhthN group. By introducing a 2,3-oxazolidinone group at the C-2 and C-3 positions, the undesired intramolecular glycosylation of monomer **14** was suppressed. However, the intramolecular glycosylation of the disaccharide intermediate afforded cyclic disaccharide **16** exclusively (Scheme 5b). Only the C-2 azido group afforded cyclic trisaccharide **19**, which contained both α- and β-glycosidic linkages (Scheme 5c). Therefore, the synthesis of a cyclic trisaccharide with only β-glycosidic linkages by electrochemical polyglycosylation was not achieved. Further optimizations of the protection group are required to suppress the formation of 1,6-anhydrosugar and cyclic disaccharides.

## Conclusion

We investigated the synthesis of cyclic β-1,6-oligoglucosamines by electrochemical polyglycosylation. The choice of the protecting group of the monomers was important to prevent intramolecular glycosylation, which formed 1,6-anhydrosugars as side products. It was revealed that the formation of cyclic disaccharides must be controlled to produce cyclic β-1,6-trisaccharides. Further optimizations of monomers and another synthetic approach using dimers for production of larger cyclic oligosaccharides are in progress in our laboratory.

## Experimental

Electrochemical polyglycosylation (Scheme 3) was performed using our second-generation automated electrochemical synthesizer equipped with the H-type divided electrolysis cell. Thioglycoside **14** (0.40 mmol, 186 mg), Bu_4_NOTf (1.0 mmol, 393 mg), DTBMP (2.0 mmol, 411 mg), and dry CH_2_Cl_2_ (10 mL) were added to the anodic chamber. Triflic acid (0.4 mmol, 35 μL) and CH_2_Cl_2_ (10 mL) were added to the cathodic chamber. Electrolysis was performed at −20 °C under constant current conditions until 1.2 F/mol of total charge had been consumed. Then, the reaction temperature was elevated to 0 °C, and this temperature was kept for 1 h. The reaction was quenched with Et_3_N (0.5 mL), and the reaction mixture was dissolved in EtOAc and washed with water to remove electrolyte. It was further washed with aqueous 1 M HCl solution and dried over Na_2_SO_4_. Then, the solvent was removed under reduced pressure, and the crude product (220 mg) was purified with recycling preparative gel permeation chromatography equipped with two series-connected JAIGEL-2HH columns (eluent: CHCl_3_, flow rate: 7.5 mL/min, recycle numbers: 3) to obtain pure cyclic oligosaccharide **16** (0.125 mmol, 79.7 mg, 62%).

## Supporting Information

File 1 Synthetic details, DFT calculations, and compound characterization data.

## Data Availability

All data that supports the findings of this study is available in the published article and/or the supporting information to this article.
