# Supplementary material for: Synthesis of cyclic β-1,6-oligosaccharides from glucosamine monomers by electrochemical polyglycosylation
Source: Beilstein J Org Chem. 2024 Jun 26;20:1421–7. doi: 10.3762/bjoc.20.124 (PMC11216080; doi:10.3762/bjoc.20.124)

BEILSTEIN JOURNAL OF ORGANIC CHEMISTRY

Supporting Information

for

Synthesis of cyclic β-1,6-oligosaccharides from glucosamine monomers by electrochemical polyglycosylation

Md Azadur Rahman, Hirofumi Endo, Takashi Yamamoto, Shoma Okushiba, Norihiko Sasaki and Toshiki Nokami

Beilstein J. Org. Chem. **2024,** *20,* 1421–1427. doi:10.3762/bjoc.20.124

Synthetic details, DFT calculations, and compound characterization data

License and Terms: This is a supporting information file under the terms of the Creative Commons Attribution License (https://creativecommons.org/licenses/by/4.0). Please note that the reuse, redistribution and reproduction in particular requires that the author(s) and source are credited and that individual graphics may be subject to special legal provisions.
The license is subject to the *Beilstein Journal of Organic Chemistry* terms and conditions: (https://www.beilstein-journals.org/bjoc/terms)

Contents

1. General S2
2. Preparation of building blocks S2
3. General procedure for cyclic oligoglucosamine with a phthalimide group S13
4. General procedure for cyclic oligoglucosamine with a 2,3-oxazolidinone group S17
5. General procedure for cyclic oligoglucosamines with an azido group S18
6. Molecular orbital calculations for the anhydrosugar **15** S21
7. References S23
8. ^{1}H and ^{13}C NMR spectra of monosaccharides and oligosaccharides S24

1. General

All reactions were conducted under argon atmosphere except when otherwise noted. ^{1}H NMR and ^{13}C NMR spectra were recorded on a Bruker AVANCE II 600 (600 MHz for ^{1}H and 150 MHz for ^{13}C) and a JEOL JNM–ECZ 600 (600 MHz for ^{1}H and 150 MHz for ^{13}C). ESI–MS spectra were recorded on a Thermo Scientific Exactive spectrometer. MALDI–TOF MS spectra were recorded on Bruker Ultraflextreme spectrometer. Merck TLC (silica gel 60 F_{254}) was used for TLC analysis. Gel permeation chromatography (GPC) was used with JAI Labo Ace LC–5060 recycling preparative HPLC (eluent: $CHCl_3$). Kanto silica gel (spherical, neutral, 63–210 µm) was used. Starting materials **S1**,[1] **S4**,[2] **S5**,[2] and **S11**[3] were prepared according to the reported procedures. All reagents were purchased from commercial suppliers and used without extra purification. Products **7a**,[4] **7b**,[5] **7c**,[6] and **8a**[7] are known compounds, and NMR spectra of these compounds were compared with reported values.

2. Preparation of building blocks

Preparation of 4-chlorophenyl-2-deoxy-2-phtalimido-1-thio-6-*O*-trityl-β-D-glucopyranoside (**S2**)

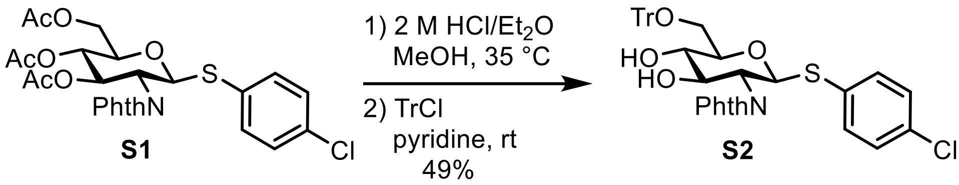

To a stirred solution of **S1**[1] (4.64 mmol, 2.61 g) in methanol (50 mL) at 35 °C was added 2 M HCl/Et_2O (10 mL). After 2 hours, the progress of the reaction was checked by TLC analysis, and the solution was concentrated under reduced pressure to afford deacetylated sugar. Then, the crude product and trityl chloride (TrCl) (6.96 mmol, 1.94 g, 1.5 equiv) were dissolved in pyridine (20 mL). The reaction mixture was kept stirred overnight, at which point the TLC analysis indicated consumption of the starting material. The reaction was quenched with excessive methanol, and the solvent was removed under reduced pressure. The crude product was purified with silica gel chromatography (hexane/EtOAc 3:2) to obtain **S2** (2.29 mmol, 1.55 g, 49%) as a white solid.

4-Chlorophenyl-2-deoxy-2-phtalimido-1-thio-6-*O*-trityl-β-D-glucopyranoside (**S2**): TLC (eluent: hexane/EtOAc 1:1) $R_f = 0.22$; ^{1}H NMR ($CDCl_3$, 600 MHz) d 7.92–7.82 (m, 2 H), 7.78–7.74 (m, 2 H), 7.49–7.45 (m, 6 H), 7.42–7.39 (m, 2 H), 7.36–7.32 (m, 6 H), 7.30–7.27 (m, 3 H), 7.21–7.18 (m, 2 H), 5.56 (d, *J* = 10.3 Hz, 1 H), 4.30 (ddd, *J* = 12.7, 8.6, 4.1 Hz, 1 H), 4.20 (*pseudo*–t, *J* = 10.3 Hz, 1 H), 3.62 (td, *J* = 8.8, 3.2 Hz, 1 H), 3.59–3.54 (m, 1 H), 3.53 (dd, *J* = 10.0, 4.0 Hz, 1 H), 3.45 (dd, *J* = 10.0, 4.5 Hz, 1 H) 2.50 (d, *J* = 3.2 Hz, 1 H), 2.36 (d, *J* = 4.3 Hz, 1 H); ^{13}C NMR ($CDCl_3$, 150 MHz) d 143.6, 134.4, 134.3, 130.3, 129.1, 128.6, 128.0, 127.3, 87.1, 83.2, 78.3, 72.9, 72.7, 63.6, 56.2; HRMS (ESI) *m/z* calcd for $C_{39}H_{32}ClKNO_6S$; $[M+K]^+$, 716.1271, found 716.1211.

Preparation of 4-chlorophenyl-3,4-di-*O*-benzoyl-2-deoxy-2-phthalimido-6-*O*-trityl-1-thio-β-D-glucopyranoside (**S3**)

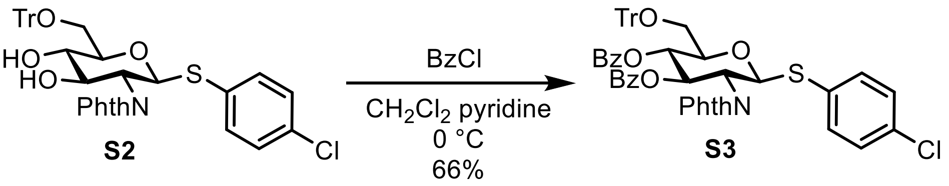

To a stirred solution of **S2** (2.19 mmol, 1.48 g) in CH_2Cl_2 (25 mL) and pyridine (5.0 mL) was added benzyl chloride (BzCl) (11.0 mmol, 0.890 mL, 5.0 equiv) dropwise. The reaction was kept stirring over a period of 4 hours at 0 °C, and the progress of the reaction was checked by TLC analysis. The solution was concentrated under the reduced pressure. The mixture was diluted with in EtOAc (100 mL) and washed with 1 M HCl aqueous solution (50 mL × 2), saturated aqueous $NaHCO_3$ solution (50 mL × 2), and brine (50 mL). The organic layer was dried over anhydrous Na_2SO_4, filtered and concentrated under reduced pressure to give the crude product. The crude product was purified with silica gel chromatography (hexane/EtOAc 3:1) to afford **S3** (1.45 mmol, 1.28 g, 66%) as a white solid.
4-Chloropheny-3,4-di-*O*-benzoyl-2-deoxy-2-phthalimido-6-*O*-trityl-1-thio-β-D-glucopyranoside (**S3**): TLC (eluent: hexane/EtOAc 2:1) R_f = 0.55; ^{1}H NMR ($CDCl_3$, 600 MHz) d 7.92–7.88 (m, 2 H), 7.77–7.65 (m, 7 H), 7.55–7.52 (m, 2 H), 7.50–7.46 (m, 1 H), 7.44–7.40 (m, 6 H), 7.30–7.27 (m, 4 H), 7.24–7.22 (m, 2 H), 7.20–7.15 (m, 6 H), 7.14–7.12 (m, 3 H); 6.14 (dd, *J* = 10.3, 9.3 Hz, 1 H), 5.84 (d, *J* = 10.5 Hz, 1 H), 5.58 (*pseudo*–t, *J* = 9.8 Hz, 1 H), 4.61 (*pseudo*–t, *J* = 6.7 Hz, 1 H), 3.98 (ddd, *J* = 10.1, 5.3, 2.3 Hz, 1 H), 3.35 (dd, *J* = 11.0, 2.2 Hz, 1 H), 3.30 (dd, *J* = 10.6, 7.6 Hz, 1 H); ^{13}C NMR ($CDCl_3$, 150 MHz) d 168.1, 167.0, 165.8, 164.8, 143.6, 134.9, 134.8, 134.4, 134.3, 133.3, 133.1, 131.7, 129.8, 129.8, 129.7, 129.2, 129.0, 128.6, 128.6, 128.3, 128.2, 127.8, 127.0, 123.8, 123.7, 86.8, 83.2, 78.2, 72.4, 69.4, 62.4, 54.0; HRMS (ESI) *m/z* calcd for $C_{53}H_{40}ClKNO_8S$; $[M+K]^+$, 924.1795, found 924.1733.

Preparation of 4-chlorophenyl-3,4-di-*O*-benzoyl-2-deoxy-2-phtalimido-1-thio-β-D-glucopyranoside (**6a**)

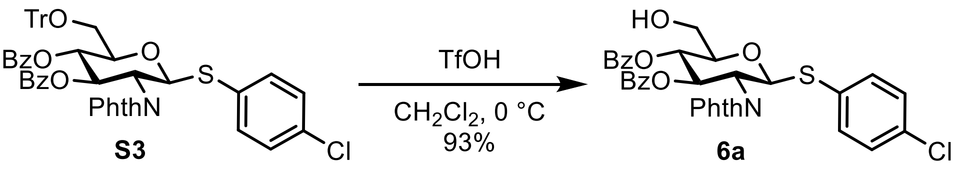

To a stirred solution of **S3** (1.49 mmol, 1.32 g) in CH_2Cl_2 (20 mL) was added trifluoromethanesulfonic acid (TfOH) dropwise (1.63 mmol, 143 µL, 1.1 equiv) at 0 °C. The reaction was kept stirring until the reaction complete (*ca*. 3 hours). The reaction was quenched with saturated aqueous $NaHCO_3$ solution, and the solvent was removed under the reduced pressure. The reaction mixture was dissolved in EtOAc

(50 mL) and washed with H_2O (50 mL × 3). The organic layer was dried over anhydrous Na_2SO_4, filtered and concentrated under reduced pressure to obtain the crude product. The crude product was purified with silica gel chromatography (hexane/EtOAc 3:1) to afford **6a** (1.38 mmol, 889 mg, 93%) as a white solid.

4-Chlorophenyl-3,4-di-*O*-benzoyl-2-deoxy-2-phtalimido-1-thio-β-D-glucopyranoside (**6a**): TLC (eluent: hexane/EtOAc 1:1) R_f = 0.54; ^{1}H NMR ($CDCl_3$, 600 MHz) d 7.95–7.92 (m, 2 H), 7.92–7.87 (m, 1 H), 7.76–7.72 (m, 4 H), 7.72–7.67 (m, 1 H), 7.54–7.49 (m, 1 H), 7.45–7.35 (m, 5 H), 7.32–7.27 (m, 3 H), 7.26–7.24 (m, 1 H), 6.32 (*pseudo*–t, *J* = 9.8 Hz, 1 H), 5.85 (d, *J* = 10.7 Hz, 1 H), 5.49 (*pseudo*–t, *J* = 9.6 Hz, 1 H), 4.56 (*pseudo*–t, *J* = 10.2 Hz, 1 H) 3.95–3.85 (m, 2 H), 3.73 (dd, *J* = 8.0, 4.6 Hz, 1 H); 2.51 (*pseudo*–t, *J* = 7.9 Hz, 1 H); ^{13}C NMR ($CDCl_3$, 150 MHz) d δ 168.1, 166.9, 166.0, 165.7, 135.0, 134.8, 134.5, 134.3, 133.7, 133.4, 131.5, 131.1, 130.0, 129.7, 129.3, 129.3, 128.5, 128.5, 128.5, 128.4, 123.8, 83.1, 78.8, 71.7, 69.7, 61.5, 53.9; HRMS (ESI) *m/z* calcd for $C_{34}H_{26}ClKNO_8S$; $[M+K]^+$, 682.0700, found 682.0688.

Preparation of 4-chlorophenyl-3-*O*-acetyl-4-*O*-benzyl-2-deoxy-2-phtalimido-1-thio-β-D-glucopyranoside (**6b**)

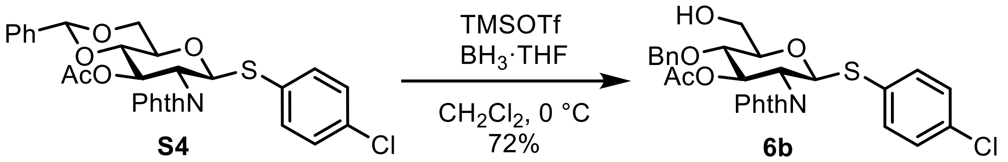

To a stirred solution of **S4**[2] (5.51 mmol, 3.12 g) in CH_2Cl_2 (24 mL) was added BH_3·THF (6.0 mL) at 0 °C. Then, trimethylsilyl trimethylsilyl trifluoromethanesulfonate (TMSOTf) (7.69 mmol, 1.39 mL, 1.4 equiv) was added dropwise. After 3 hours, the completion of the reaction was checked TLC analysis, and the reaction was quenched with saturated aqueous $NaHCO_3$ solution. Then, the solvent was removed under reduced pressure. The mixture was dissolved in EtOAc (100 mL) and washed with H_2O (100 mL × 3). The organic layer was dried over anhydrous Na_2SO_4, filtered and concentrated under reduced pressure to give the crude product. The crude product was purified with silica gel chromatography (hexane/EtOAc 1:1) to obtain **6b** (3.97 mmol, 2.26 g, 72%) as a white solid.

4-Chlorophenyl-3-*O*-acetyl-4-*O*-benzyl-2-deoxy-2-phtalimido-1-thio-β-fD-glucopyranoside (**6b**): TLC (eluent: hexane/EtOAc 1:1) R_f = 0.48; ^{1}H NMR ($CDCl_3$, 600 MHz) d 7.90–7.83 (m, 2 H), 7.77–7.73 (m, 2 H), 7.35–7.31 (m, 4 H), 7.29–7.27 (m, 3 H), 7.25–7.23 (m, 2 H), 5.79 (*pseudo*–t, *J* =9.5 Hz, 1 H), 5.74 (d, *J* = 5.3 Hz, 1 H), 4.67 (d, *J* = 11.4 Hz, 1 H), 4.64 (d, *J* = 11.5 Hz, 1 H), 4.21 (*pseudo*–t, *J* = 10.2 Hz, 1 H), 3.95 (dd, *J* = 11.6, 3.5 Hz, 1 H), 3.80–3.76 (m, 1 H), 3.76 (*pseudo*–t, *J* = 8.0 Hz, 1 H) 3.65 (m, 1 H), 1.83 (dd, *J* = 8.5, 5.5 Hz, 1 H), 1.76 (s, 3 H); ^{13}C NMR ($CDCl_3$, 150 MHz) d ^{13}C NMR (151 MHz, $CDCl_3$) δ 170.1, 167.8, 167.3, 137.6, 134.7, 134.5, 134.4, 134.2, 131.6,

131.1, 129.6, 129.2, 128.5, 123.0, 127.8, 123.7, 123.6, 82.8, 79.5, 75.9, 74.8, 73.9, 61.7, 54.1, 20.5; HRMS (ESI) *m/z* calcd for $C_{29}H_{26}ClKNO_7S$; $[M+K]^+$, 606.0751, found 606.0741.

Preparation of 4-chlorophenyl-3-*O*-benzyl-4,6-*O*-benzylidene-2-deoxy-2-phthalimido-1-thio-β-D-glucopyranoside (**S6**)

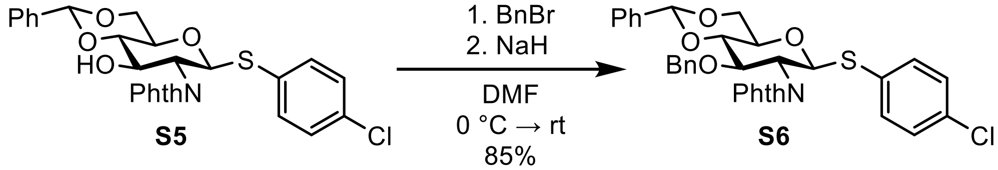

S5 **S6**

To a stirred solution of **S5**[2] (1.33 mmol, 0.696 g) in DMF (15 mL) was added benzyl bromide (BnBr) (4.00 mmol, 476 µL) at 0 °C. Then, 60% sodium hydride (NaH) (160 mg, 4.00 mmol) in DMF (4 mL) was added dropwise. The reaction was kept stirring overnight and the progress of the reaction was checked by TLC analysis. The reaction was quenched with saturated aqueous $NaHCO_3$ solution, and the solution was diluted with in EtOAc (50 mL) and washed with 1 M HCl aqueous solution (50 mL × 2), saturated aqueous $NaHCO_3$ solution (50 mL × 2), and brine (50 mL). The organic layer was dried over anhydrous Na_2SO_4, filtered and concentrated under reduced pressure to give the crude product. The crude product was purified with silica gel chromatography (hexane/EtOAc 5:1) to afford **S6** (1.13 mmol, 0.690 g, 85%) as a white solid.

4-Chlorophenyl-3-*O*-benzyl-4,6-*O*-benzylidene-2-deoxy-2-phthalimido-1-thio-β-D-glucopyranoside (**S6**): TLC (eluent: hexane/EtOAc 5:1) R_f = 0.39; ^{1}H NMR (600 MHz, $CDCl_3$) δ 7.89–7.84 (m, 1 H), 7.77–7.70 (m, 2 H), 7.66–7.60 (m, 1 H), 7.53–7.48 (m, 2 H), 7.44–7.36 (m, 3 H), 7.33–7.28 (m, 2 H), 7.24–7.20 (m, 2 H), 6.98–6.95 (m, 2 H), 6.93–6.88 (m, 1 H), 6.87–6.84 (m, 2 H), 5.63 (s, 1 H), 5.60 (d, *J* = 10.6 Hz, 1 H), 4.75 (d, *J* = 12.3 Hz, 1 H), 4.48 (d, *J* = 12.4 Hz, 1 H), 4.42 (dd, *J* = 10.5, 4.9 Hz, 1 H), 4.40 (*pseudo*-t, *J* = 9.3 Hz, 1 H), 4.24 (*pseudo*-t, *J* = 10.3 Hz, 1 H), 3.83 (*pseudo*-t, *J* = 10.3 Hz, 1 H), 3.77 (*pseudo*-t, *J* = 9.1 Hz, 1 H), 3.70 (td, *J* = 9.7, 4.9 Hz, 1 H); ^{13}C NMR (150 MHz, $CDCl_3$) δ 167.8, 167.2, 137.7, 137.3, 134.7, 134.6, 134.1, 134.0, 131.5, 129.7, 129.1, 128.3, 128.2, 128.1, 127.5, 126.1, 123.6, 123.4, 101.4, 83.8, 82.8, 75.4, 74.3, 70.4, 68.6, 54.7; HRMS (ESI) *m/z* calcd for $C_{34}H_{28}ClKNO_6S$ $[M+Na]^+$, 652.0958; found, 652.0943.

Preparation of 4-chlorophenyl-3,4-di-*O*-benzyl-2-deoxy-2-phthalimido-1-thio-β-D-glucopyranoside (**6c**)

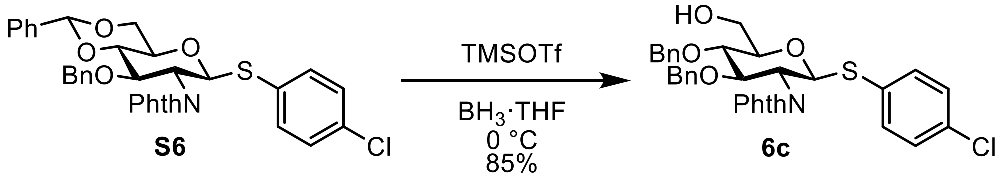

To a stirred solution of **S6** (1.38 mmol, .849 mg) and BH_3·THF (7.0 mL), TMSOTf (0.70 mL) was added dropwise at 0 °C. Then, the mixture was stirred for 4 hours at room temperature. The reaction was quenched with saturated aqueous $NaHCO_3$ solution, and the solution was diluted in EtOAc. The mixture was washed with saturated aqueous $NaHCO_3$ solution (3 times), H_2O (3 times) and brine respectively. The organic layer was dried over anhydrous Na_2SO_4, filtered, and concentrated under reduced pressure. The crude product was purified with silica gel chromatography (hexane/EtOAc 3:1) to give **6c** (1.17 mmol, 730 mg, 85%) as a white solid.

4-Chlorophenyl-3,4-di-*O*-benzyl-2-deoxy-2-phthalimido-1-thio-β-D-glucopyranoside (**6c**): TLC (eluent: hexane/EtOAc 2:1) R_f = 0.50; ^{1}H NMR (600 MHz, $CDCl_3$) δ 7.81–7.60 (m, 4 H), 7.37–7.32 (m, 4 H), 7.31–7.25 (m, 3 H), 7.20–7.18 (m, 2 H), 6.99–6.97 (m, 2 H), 6.89–6.82 (m, 3 H), 5.54 (d, *J* = 10.5 Hz, 1 H), 4.87 (d, *J* = 10.8 Hz, 1 H), 4.79 (d, *J* = 12.3 Hz, 1 H), 4.72 (d, *J* = 10.8 Hz, 1 H), 4.44 (d, *J* = 12.3 Hz, 1 H), 4.37 (dd, *J* = 10.2, 9.0 Hz, 1 H), 4.19 (*pseudo*–t, *J* = 10.5 Hz, 1 H), 3.93 (dd, *J* = 12.3, 2.4 Hz, 1 H), 3.76 (dd, *J* = 12.3, 4.5 Hz, 1 H), 3.72–3.68 (m, 1 H), 3.59 (dd, *J* = 9.9, 2.4 Hz, 1 H), 2.16–2.04 (m, 1 H); ^{13}C NMR (150 MHz, $CDCl_3$) δ 168.2, 167.5, 138.0, 137.9, 134.3, 134.2, 134.1, 133.9, 131.7, 131.5, 130.6, 129.2, 128.7, 128.3, 128.2, 128.1, 127.6, 123.6, 123.6, 83.4, 80.2, 80.0, 79.3, 77.7, 77.5, 77.3, 75.2, 75.1, 61.8, 55.1; HRMS (ESI) *m/z* calcd for $C_{34}H_{30}ClNNaO_6S$ $[M+Na]^+$, 638.1375; found, 638.1357.

Preparation of 4-chlorophenyl-3-*O*-acetyl-4-*O*-benzyl-6-*O*-*tert*-butyldiphenylsilyl-2-deoxy-1-thio-β-D-glucopyranoside (**S7**)

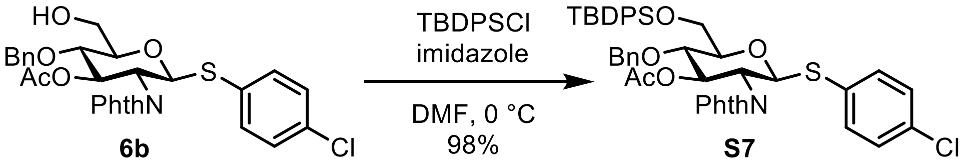

To a stirred solution of **6b** (3.61 mmol, 2.05 g) and imidazole (14.4 mmol, 1.06 g, 4.0 equiv) in DMF (15 mL) was added *tert*-butyldiphenylsilyl chloride (TBDPSCl) (10.8 mmol, 3.16 mL, 3.0 equiv) dropwise at 0 °C. The completion of the reaction was monitored by TLC analysis, and the reaction was quenched with saturated aqueous $NaHCO_3$ solution. The mixture was dissolved in EtOAc (100 mL) and washed with 1 M HCl aqueous solution (100 mL × 2), saturated aqueous $NaHCO_3$ solution (50 mL × 2), and brine (50 mL). The organic layer was dried over anhydrous Na_2SO_4, filtered and

concentrated under reduced pressure to give the crude product. The crude product was purified with silica gel chromatography (hexane/EtOAc 5:1) to afford **S7** (3.52 mmol, 2.84 g, 98%) as a white solid.
4-Chlorophenyl-3-*O*-acetyl-4-*O*-benzyl-6-*O*-*tert*-butyldiphenylsilyl-2-deoxy-1-thio-β-D-glucopyranoside (**S7**): TLC (eluent: hexane/EtOAc 3:1) R_f = 0.47; ^{1}H NMR ($CDCl_3$, 600 MHz) d 7.90–7.85 (m, 2 H), 7.80–7.77 (m, 2 H), 7.76–7.74 (m, 2 H), 7.72–7.69 (m, 2 H), 7.46–7.43 (m, 2 H), 7.40–7.35 (m, 6 H), 7.26-7.23 (m, 3 H), 7.15–7.10 (m, 4 H), 5.80 (*pseudo*–t, *J* = 9.5 Hz, 1 H), 5.71 (d, *J* = 10.4 Hz, 1 H), 4.68 (d, *J* = 11.6 Hz, 1 H), 4.63 (d, *J* = 11.6 Hz, 1 H), 4.26 (*pseudo*–t, *J* = 10.3 Hz, 1 H), 4.05–4.01 (m, 1 H), 3.97 (dd, *J* = 11.7, 2.8 Hz, 1 H), 3.93 (*pseudo*–t, *J* = 9.5 Hz, 1 H), 3.65 (dd, *J* = 10.1, 1.3 Hz, 1 H), 1.76 (s, 3 H), 1.12 (s, 9 H); ^{13}C NMR ($CDCl_3$, 150 MHz) d 170.2, 167.9, 167.4, 137.9, 135.9, 135.6, 134.5, 134.4, 134.3, 134.2, 133.3, 132.8, 131.8, 131.2, 130.0, 129.8, 129.1, 128.5, 127.83, 127.79, 127.76, 127.5, 123.7, 123.6, 82.7, 80.1, 76.2, 74.9, 74.2, 62.5, 54.2, 26.9, 20.6, 19.4; HRMS (ESI) *m/z* calcd for $C_{45}H_{44}ClKNO_7SSi$; $[M+K]^+$, 844.1928, found 844.1914.

Preparation of 4-chlorophenyl-2-amino-4-*O*-benzyl-6-*O*-*tert*-butyldiphenylsilyl-2-deoxy-1-thio-β-D-glucopyranoside (**S8**)

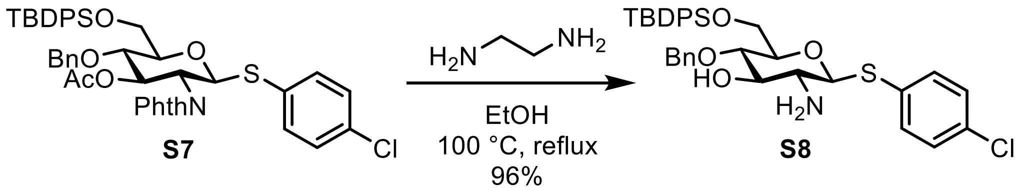

To a stirred solution of **S7** (3.82 mmol, 3.07 g) in ethanol (30 mL) was added ethylenediamine anhydrous (6.4 mL) at room temperature. The reaction temperature was gradually raised from room temperature to 100 °C and kept stirring. The progress of the reaction was checked by TLC analysis, and heating was halted in 3 hours. The solvent was removed under the reduced pressure, and the mixture was purified with silica gel chromatography (hexane/EtOAC 1:3 + 1% Et_3N) to obtain **S8** (3.65 mmol, 2.31 g, 96%) as a white solid.
4-Chlorophenyl-2-amino-4-*O*-benzyl-6-*O*-*tert*-butyldiphenylsilyl-2-deoxy-1-thio-β-D-glucopyranoside (**S8**): TLC (eluent: hexane/EtOAc 1:3) R_f = 0.38; ^{1}H NMR ($CDCl_3$, 600 MHz) d 7.79–7.76 (m, 2 H), 7.73–7.70 (m, 2 H), 7.51–7.48 (m, 2 H), 7.45–7.40 (m, 2 H), 7.37–7.33 (m, 4 H), 7.32–7.27 (m, 3 H), 7.25–7.22 (m, 2 H), 7.17–7.13 (m, 2 H), 4.80 (d, *J* = 11.2 Hz, 1 H), 4.71 (d, *J* = 11.2 Hz, 1 H), 4.40 (d, *J* = 9.9 Hz, 1 H), 4.03–3.99 (m, 1 H), 3.95 (dd, *J* = 11.2, 3.9 Hz, 1 H), 3.64 (*pseudo–t*, *J* = 9.3 Hz, 1 H); 3.48 (*pseudo*–t, *J* = 9.2 Hz, 1 H), 3.41 (dd, *J* = 9.8, 2.5 Hz, 1 H), 2.69 (*pseudo*–t, *J* = 9.6 Hz, 1 H), 1.10 (s, 9 H); ^{13}C NMR ($CDCl_3$, 150 MHz) d 138.3, 135.9, 135.6, 134.0, 133.6, 133.4, 133.0, 131.2, 129.8, 129.7, 129.1, 128.6, 127.9, 127.9, 127.8, 127.7, 89.3, 80.0, 78.3, 77.5, 74.8, 62.9, 56.1, 26.9, 19.4; HRMS (ESI) *m/z* calcd for $C_{35}H_{40}ClNNaO_4SSi$; $[M+Na]^+$, 656.2029, found 656.2006.

Preparation of 4-chlorophenyl-4-*O*-benzyl-6-*O*-*tert*-butyldiphenylsilyl-2,3-*N*,*O*-carbonyl-2-deoxy-1-thio-β-D-glucopyranoside (**S9**)

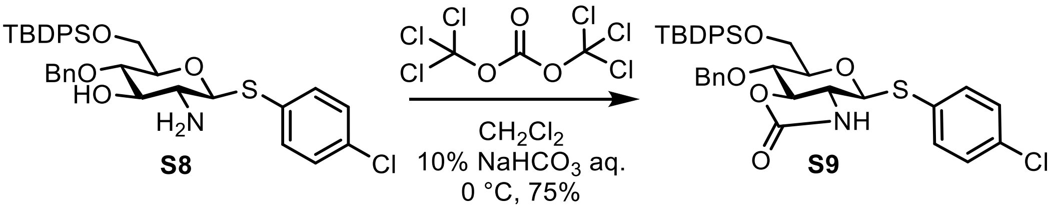

To a stirred solution of **S8** (3.59 mmol, 2.28 g) in CH_2Cl_2 (50 mL) and 10% aqueous $NaHCO_3$ solution was added triphosgene (1.38 mmol, 408 mg, 0.383 equiv) at room temperature. The reaction was kept stirring for overnight. The completion of the reaction was cheeked by TLC analysis, then the solution was extracted with CH_2Cl_2 (50 mL × 3). The organic layer was dried over anhydrous Na_2SO_4, filtered and concentrated under reduced pressure. The crude product was purified with silica gel chromatography (hexane/EtOAc = 5:1) to obtain **S9** (2.69 mmol, 1.78 g, 75%) as a white solid.

4-Chlorophenyl-4-*O*-benzyl-6-*O*-*tert*-butyldiphenylsilyl-2,3-*N*,*O*-carbonyl-2-deoxy-1-thio-β-D-glucopyranoside (**S9**): TLC (eluent: hexane/EtOAc 3:1) R_f = 0.46; ^{1}H NMR ($CDCl_3$, 600 MHz) d 7.75–7.72 (m, 2 H), 7.71–7.68 (m, 2 H), 7.50–7.47 (m, 2 H), 7.45–7.42 (m, 2 H), 7.38–7.33 (m,4 H), 7.30–7.28 (m, 3 H), 7.25–7.18 (m, 4 H), 5.00 (s, 1 H), 4.88 (d, J = 10.5 Hz, 1 H), 4.73 (d, J = 11.2 Hz, 1 H), 4.57 (d, J = 11.0 Hz, 1 H), 4.28 (*pseudo*–t, J = 10.4 Hz, 1 H), 4.04 (*pseudo*–t, J = 9.4 Hz, 1 H), 4.00–3.7 (m, 2 H), 3.56 (dd, J = 8.7, 3.0 Hz, 1 H), 3.44 (*pseudo*–t, J = 10.6 Hz, 1 H) 1.07 (s, 9 H); ^{13}C NMR ($CDCl_3$, 150 MHz) d 159.0, 137.2, 135.7, 135.6, 135.1, 134.6, 133.2, 133.0, 129.8, 129.4, 129.1, 128.5, 128.0, 128.0, 127.8, 127.8, 85.4, 84.2, 81.3, 73.5, 73.2, 62.4, 58.5, 26.8, 19.4; HRMS (ESI) *m/z* calcd for $C_{36}H_{38}ClKNO_5SSi$; $[M+K]^+$, 698.1561, found 698.1538.

Preparation of 4-chlorophenyl-2-acetamido-4-*O*-benzyl-6-*O*-*tert*-butyldiphenylsilyl-2,3-*N*,*O*-carbonyl-2-deoxy-1-thio-β-D-glucopyranoside (**S10**)

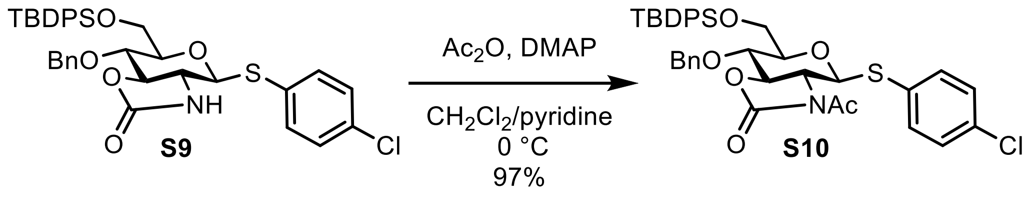

To a stirred solution of **S9** (2.54 mmol, 1.68 g) and 4-dimethylaminopyridine (DMAP) (1.27 mmol, 154 mg, 0.5 equiv) in CH_2Cl_2 (15 mL) and pyridine (3.0 mL) was added acetic anhydride (5.0 mL) at 0 °C. The reaction was monitored by TLC analysis and quenched with methanol after the completion (*ca.* 2 hours). The solvent was removed under reduced pressure. The mixture was diluted with in EtOAc (50 mL) and washed with 1 M HCl aqueous solution (50 mL × 3), saturated aqueous $NaHCO_3$ solution (50 mL × 3), and brine (50 mL). The organic layer was dried over anhydrous Na_2SO_4, filtered

and concentrated under reduced pressure to give the purified product **S10** (2.46 mmol, 1.72 g, 97%) as a white solid.

4-Chlorophenyl-2-acetamido-4-*O*-benzyl-6-*O*-*tert*-butyldiphenylsilyl-2,3-*N*,*O*-carbonyl-2-deoxy-1-thio-β-ᴅ-glucopyranoside (**S10**): TLC (eluent: hexane/EtOAc 3:1) R_f = 0.67; ^{1}H NMR ($CDCl_3$, 600 MHz) d 7.75–7.70 (m, 2 H), 7.68–7.65 (m, 2H), 7.45–7.40 (m, 4 H), 7.39–7.34 (m, 4 H), 7.31–7.28 (m, 3 H), 7.25–7.20 (m, 2 H), 7.16–7.13 (m, 2 H), 4.88 (d, *J* = 11.2 Hz, 1 H), 4.82 (d, *J* = 8.5 Hz, 1 H), 4.60 (d, *J* = 11,5 Hz, 1 H), 4.27 (*pseudo*–t, *J* = l0.6 Hz, 1 H), 4.11-4.95 (m, 2 H), 3.92 (dd, *J* = 11.4, 4.2 Hz, 1 H), 3.88–3.84 (m, 1 H), 3.49 (dd, *J* = 8.2, 2.3 Hz, 1 H), 2.60 (s, 3 H), 1.05 (s, 9 H); ^{13}C NMR ($CDCl_3$, 150 MHz) d 173.2, 154.0, 137.0, 135.8, 135.6, 134.1, 133.6, 133.2, 132.8, 132.7, 129.9, 129.8, 129.0, 128.5, 128.1, 128.0, 127.8, 127.8, 87.0, 82.8, 81.2, 74.0, 73.6, 62.5, 59.8, 26.9, 24.9, 19.3; HRMS (ESI) *m/z* calcd for $C_{38}H_{40}ClKNO_6SSi$; $[M+K]^+$, 740.1666, found 740.1654.

Preparation of 4-chlorophenyl-2-acetamido-4-*O*-benzyl-2,3-*N*,*O*-carbonyl-2-deoxy-1-thio-β-ᴅ-glucopyranoside (**14**)

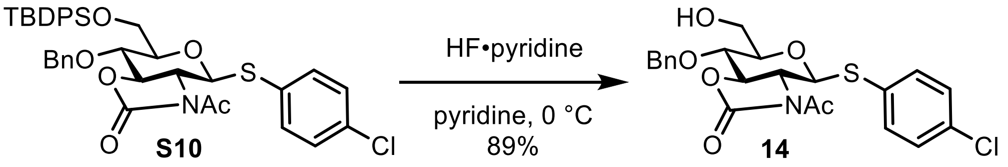

To a stirred solution of **S10** (2.32 mmol, 1.63 g) in pyridine (20 mL) was added HF/pyridine (19.2 mmol, 2.53 mL, 8.3 equiv) dropwise at 0 ºC. The reaction was monitored by TLC analysis and quenched with saturated aqueous $NaHCO_3$ solution when the reaction completed. The mixture was diluted with in EtOAc (50 mL) and washed with 1 M HCl aqueous solution (50 mL × 3), saturated aqueous $NaHCO_3$ solution (50 mL × 3), and brine (50 mL). The organic layer was dried over anhydrous Na_2SO_4, filtered and concentrated under reduced pressure to obtain the crude product. The crude product was purified with silica gel chromatography to afford **14** (2.07 mmol, 963 mg, 89%) as a white solid.

4-Chlorophenyl-2-acetamido-4-*O*-benzyl-2,3-*N*,*O*-carbonyl-2-deoxy-1-thio-β-ᴅ-glucopyranoside (**14**): TLC (eluent: hexane/EtOAc 3:1) R_f = 0.36; ^{1}H NMR ($CDCl_3$, 600 MHz) d 7.43–7.40 (m, 2 H), 7.39–7.35 (m, 2 H), 7.35–7.30 (m, 3 H), 7.30–7.27 (m, 2 H), 4.894 (d, *J* = 11.0 Hz, 1 H), 4.892 (d, *J* = 8.6 Hz, 1 H), 4.63 (d, *J* = 11.4 Hz, 1 H), 4.38 (*pseudo*–t, *J* = 10.7 Hz, 1 H), 4.03 (dd, *J* = 11.3, 8.8 Hz, 1 H), 3.93 (*pseudo*–t, *J* = 9.3 Hz, 1 H), 3.82 (ddd, *J* = 11.5, 6.4, 2.2 Hz, 1 H), 3.72 (ddd, *J* = 13.7, 7.7, 4.5 Hz, 1 H), 3.52–3.46 (m, 1 H), 2.59 (s, 3 H), 1.84 (*pseudo*–t, *J* = 7.0 Hz, 1 H); ^{13}C NMR ($CDCl_3$, 150 MHz) d 173.1, 153.7, 136.9, 134.4, 133.5, 132.1, 129.2, 128.6, 128.3, 128.1, 86.7, 82.5, 80.7, 73.7, 73.5, 61.5, 59.8, 24.8; HRMS (ESI) *m/z* calcd for $C_{22}H_{22}ClKNO_6S$; $[M+K]^+$, 502.0488, found 502.0474.

Preparation of 4-chlorophenyl-3-*O*-acetyl-2azido-4,6-*O*-benzylidene-2-deoxy-1-thio-β-D-glucopyranoside (**S12**)

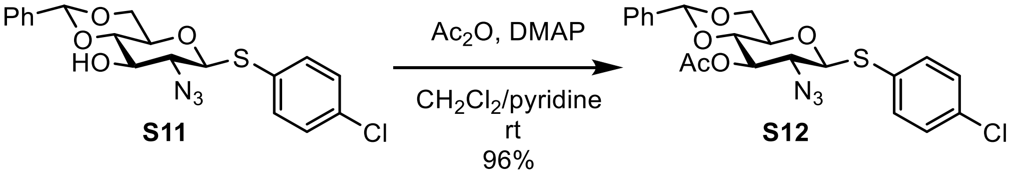

To a stirred solution of **S11**[3] (6.45 mmol, 2.70 g) and DMAP (84 mg) in CH_2Cl_2 (24 mL) and pyridine (7.0 mL), acetic anhydride (4.0 mL) was added dropwise. Then, the mixture was stirred overnight at room temperature. The reaction was quenched with 1 M HCl aqueous solution, and the mixture was diluted in EtOAc, and the organic solution was washed with 1 M HCl aqueous solution (3 times), saturated aqueous $NaHCO_3$ solution (3 times), H_2O (3 times), and brine respectively. The organic layer was dried over anhydrous Na_2SO_4, filtered, and concentrated under reduced pressure. The crude product was purified with silica gel chromatography (hexane/EtOAc 4:1) to afford **S12** (6.20 mmol, 2.89 g, 96%) as a white solid.

4-Chlorophenyl-3-*O*-acetyl-2-azido-4,6-*O*-benzylidene-2-deoxy-1-thio-β-D-glucopyranoside (**S12**): TLC (eluent: hexane/EtOAc 3:1) R_f = 0.50; ^{1}H NMR (600 MHz, $CDCl_3$) δ 7.50–7.48 (m, 2 H), 7.44–7.30 (m, 7 H), 5.45 (s, 1 H), 5.27 (*pseudo*–t, *J* = 9.6 Hz, 1 H), 4.55 (d, *J* = 10.2 Hz, 1 H), 4.32 (dd, *J* = 10.5, 4.8 Hz, 1 H), 3.72 (*pseudo*–t, *J* = 10.2 Hz, 1 H), 3.51 (*pseudo*–t, *J* = 9.6 Hz, 1 H), 3.46 (td, *J* = 9.6, 4.8, Hz, 1 H), 3.40 (*pseudo*–t, *J* = 9.9 Hz, 1 H), 2.10 (s, 3 H); ^{13}C NMR (150 MHz, $CDCl_3$) δ 169.6, 137.0, 135.2, 135.2, 129.5, 129.3, 128.4, 126.3, 101.5, 86.6, 78.3, 77.7, 77.5, 77.3, 73.0, 70.7, 68.3, 63.6, 20.9; HRMS (ESI) *m/z* calcd for $C_{21}H_{20}ClKN_3O_5S$ $[M+K]^+$, 500.0444; found, 500.0425.

Preparation of 4-chlorophenyl-3-*O*-acetyl-2-azido-4-*O*-benzyl-2-deoxy-1-thio-β-D-glucopyranoside (**17a**)

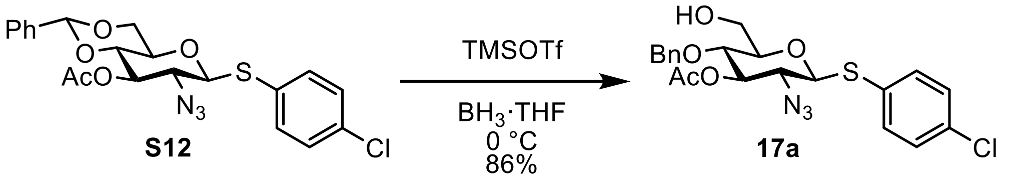

To a stirred solution of **S12** (1.37 mmol, 0.630 g), and $BH_3{\cdot}THF$ (7.0 mL), TMSOTf (0.70 mL) was added dropwise at 0 °C. Then, the mixture was stirred for 4 hours at room temperature. The reaction was quenched with saturated aqueous $NaHCO_3$ solution. The mixture was diluted in EtOAc, and the organic solution was washed with saturated aqueous $NaHCO_3$ solution (3 times), H_2O (3 times) and brine respectively. The organic layer was dried over anhydrous Na_2SO_4, filtered, and concentrated under reduced pressure. The crude product was purified with silica gel chromatography (hexane/EtOAc 5:1) to afford **17a** (1.19 mmol, 0.550 g, 86%) as a white solid.

4-Chlorophenyl-3-*O*-acetyl-2-azido-4-*O*-benzyl-2-deoxy-1-thio-β-D-glucopyranoside (**17a**): TLC

(eluent: hexane/EtOAc 2:1) R_f = 0.47; ^1H NMR (600 MHz, $CDCl_3$) δ 7.49–7.46 (m, 2 H), 7.34–7.28 (m, 5 H), 7.26–7.24 (m, 2 H), 5.15 (*pseudo*–t, *J* = 9.6 Hz, 1 H), 4.61 (d, *J* = 11.4 Hz, 1 H), 4.56 (d, *J* = 11.4 Hz, 1 H), 4.49 (d, *J* = 10.2 Hz, 1 H), 3.89 (ddd, *J* = 12.3, 5.4, 2.4 Hz, 1 H), 3.74–3.70 (m, 1 H), 3.57 (*pseudo*–t, *J* = 9.6 Hz, 1 H), 3.41 (ddd, *J* = 9.9, 3.9, 2.7 Hz, 1 H), 3.26 (*pseudo*–t, *J* = 9.9 Hz, 1 H), 2.00 (s, 3 H), 1.88 (dd, *J* = 8.0, 5.2 Hz, 1 H); ^{13}C NMR (150 MHz, $CDCl_3$) δ 169.9, 137.4, 135.2, 135.0, 129.5, 129.4, 128.7, 128.2, 128.1, 86.1, 79.7, 77.3, 77.1, 76.9, 75.9, 75.1, 74.8, 63.4, 61.6, 20.9; HRMS (ESI) *m/z* calcd for $C_{21}H_{22}ClNaN_3O_5S$ $[M+Na]^+$, 486.0861; found, 486.0842.

Preparation of 4-chlorophenyl-2-azido-3-*O*-benzyl-4,6-*O*-benzylidine-2-deoxy-1-thio-β-ᴅ-glucopyranoside (**S13**)

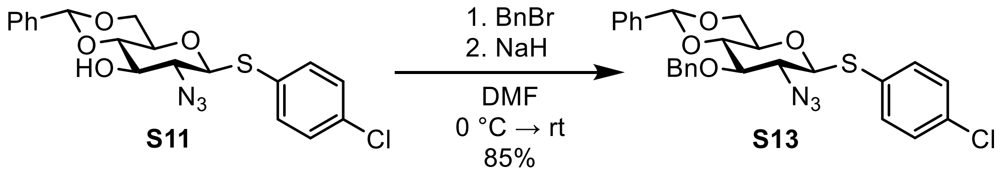

To a stirred solution of **S11**[3] (2.40 mmol, 1.01 g) in DMF (20 mL) was added 60% NaH (4.80 mmol, 192 mg) at 0 °C. Then, BnBr (3.60 mmol, 450 μL) was added dropwise, and the reaction was kept stirring overnight. The progress of the reaction was monitored by TLC analysis, and the reaction was quenched with saturated aqueous $NaHCO_3$ solution. The solution was diluted with in EtOAc (100 mL) and washed with 1 M HCl aqueous solution (50 mL × 2), saturated aqueous $NaHCO_3$ solution (50 mL × 2), and brine (50 mL). The organic layer was dried over anhydrous Na_2SO_4, filtered and concentrated under reduced pressure to give the crude product. The crude product was purified with silica gel chromatography (hexane/EtOAc 8:1) to afford **S13** (2.06 mmol, 1.05 g, 86%) as a white solid.

4-Chlorophenyl-2-azido-3-*O*-benzyl-4,6-*O*-benzylidine-2-deoxy-1-thio-β-ᴅ-glucopyranoside (**S13**): TLC (eluent: hexane/EtOAc 8:1) R_f = 0.46; ^1H NMR (600 MHz, $CDCl_3$) δ 7.51–7.46 (m, 4 H), 7.40–7.37 (m, 3 H), 7.36–7.29 (m, 7 H), 5.57 (s, 1 H), 4.91 (d, *J* = 11.0 Hz, 1 H), 4.78 (d, *J* = 11.0 Hz, 1 H), 4.44 (d, *J* = 10.1 Hz, 1 H), 4.38 (dd, J = 10.6, 5.0 Hz, 1 H), 3.77 (*pseudo*-t, *J* = 10.3 Hz, 1 H), 3.66 (*pseudo*-t, *J* = 9.1 Hz, 1 H), 3.63 (*pseudo*-t, *J* = 9.2 Hz, 1 H), 3.48–3.42 (m, 1 H), 3.33 (*pseudo*-t, *J* = 9.3 Hz, 1 H); ^{13}C NMR (150 MHz, $CDCl_3$) δ 137.5, 137.0, 135.4, 135.3, 129.4, 129.2, 129.0, 128.5, 128.4, 128.1, 126.0, 101.3, 86.5, 81.3, 80.9, 76.9, 75.2, 70.6, 68.5, 64.7; HRMS (ESI) *m/z* calcd for $C_{26}H_{24}ClKN_3O_6S$ $[M+Na]^+$, 548.0808; found, 548.0817.

Preparation of 4-chlorophenyl-2-azido-3,4-di-*O*-benzyl-2-deoxy-1-thio-β-D-glucopyranoside (**17b**)

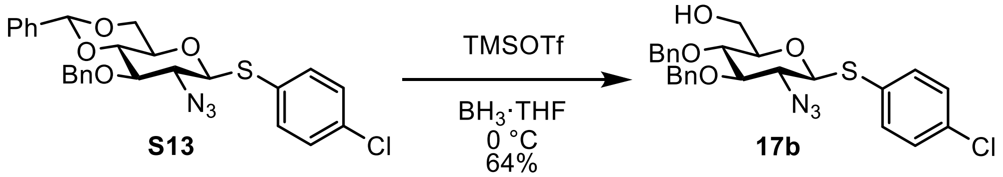

To a stirred solution of **S13** (2.31 mmol, 1.18 g), and BH_3·THF (12 mL), TMSOTf (1.2 mL) was added dropwise at 0 °C. Then, the mixture was stirred for 6 hours at room temperature. The reaction was quenched with saturated aqueous $NaHCO_3$ solution, and the reaction mixture diluted with in EtOAc. The solution was washed with saturated aqueous $NaHCO_3$ solution (3 times), H_2O (3 times) and brine respectively. The organic layer was dried over anhydrous Na_2SO_4, filtered, and concentrated under reduced pressure. The crude product was purified with silica gel chromatography (hexane/EtOAc 5:1) to obtain **1d** (1.47 mmol, 0.750 g, 64%) as a white solid.

4-Chlorophenyl-2-azido-3,4-di-*O*-benzyl-2-deoxy-1-thio-β-D-glucopyranoside (**17b**): TLC (eluent: hexane/EtOAc 3:1) R_f = 0.40; ^{1}H NMR (600 MHz, $CDCl_3$) δ 7.48–7.46 (m, 2 H), 7.36–7.27 (m, 12 H), 4.87 (d, *J* = 10.6 Hz, 1 H), 4.86 (d, *J* = 10.6 Hz, 1 H), 4.84–4.82 (m, 1 H), 4.64 (d, *J* = 11.1 Hz, 1 H), 4.41 (d, *J* = 10.2 Hz, 1 H), 3.87 (dd, *J* = 6.0, 2.7 Hz, 1 H), 3.71–3.67 (m, 1 H), 3.55–3.50 (m, 2 H), 3.36 (dd, *J* = 4.8, 2.7 Hz, 1 H), 3.30 (ddd, *J* = 10.2, 6.6, 2.7 Hz, 1 H), 1.82 (*pseudo*–t, *J* = 6.9 Hz, 1 H); ^{13}C NMR (150 MHz, $CDCl_3$) δ 137.6, 137.5, 135.1, 134f.9, 129.6, 129.4, 128.69, 128.65, 128.32, 128.21, 128.19, 128.12, 86.1, 84.9, 79.8, 76.0, 75.2, 65.3, 61.9; HRMS (ESI) *m/z* calcd for $C_{26}H_{26}ClNaN_3O_4S$ $[M+Na]^+$, 534.1225; found, 534.1211.

3. General procedure for cyclic oligoglucosamine synthesis with phthalimide group

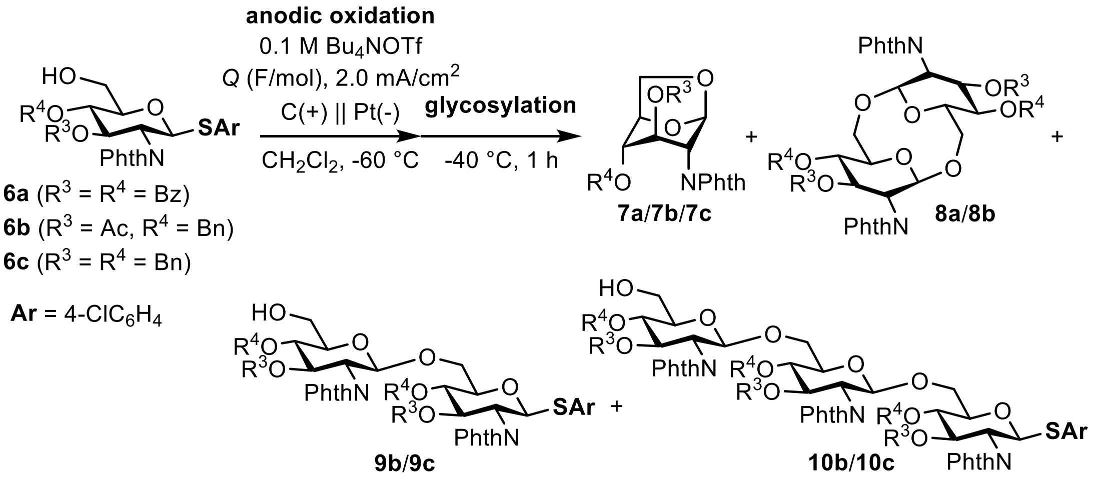

The electrochemical one–pot synthesis of cyclic oligosaccharides **8** and linear oligosaccharides **9** and **10** was conducted with an H–type divided cell (4G glass filter). The cell had a carbon felt anode (Nippon Carbon JF–20–p7) and platinum square plate (20 mm × 20 mm). Building block **6** (0.400 mmol), Bu_4NOTf (1.00 mmol), and CH_2Cl_2 (10 mL) were added to the anodic chamber. TfOH (0.400 mmol), Bu_4NOTf (1.00 mmol), and CH_2Cl_2 (10 mL) were added to the cathodic chamber. As the initiation phase of reaction, the constant current (8.0 mA (current density: 2.0 mA/cm^2), (electrode distance: 4.5 cm)) was employed at –60 °C with magnetic stirring until electricity was consumed. After the electrolysis, the reaction temperature was raised from –60 °C to –40 °C as glycosylation phase. After glycosylation, Et_3N (0.5 mL) was added to both of the chambers. The mixture was collected, and the solvent was removed under reduced pressure. The mixture was dissolved in EtOAc and washed with H_2O to remove electrolyte. The solution was dried over anhydrous Na_2SO_4, filtered, and removed under reduced pressure. The crude product was purified with preparative GPC to afford 1,6-anhydrosugar **7**,[4-6] cyclic oligosaccharides **8**[7] and linear oligosaccharides **9** and **10**.

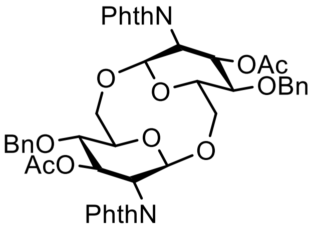

8b

Building block **6b** (226 mg, 0.400 mmol) afforded **8b** as in 7% isolated yield (11 mg, 0.0130 mmol). Cyclobis[(1→6)-3-*O*-acetyl-4-*O*-benzyl-2-deoxy-2-phthalimido-β-D-glucopyranosyl] (**8b**): TLC (hexane/EtOAc 1:1) R_f = 0.25; ^{1}H NMR (600 MHz, $CDCl_3$) δ 7.87–7.86 (m, 2 H), 7.74–7.72 (m, 2 H), 7.39–7.38 (m, 1 H), 7.32–7.26 (m, 4 H), 5.70 (dd, *J* = 10.8, 8.7 Hz, 1 H), 4.93 (d, *J* = 1.8 Hz, 1 H),

4.78 (d, J = 11.7 Hz, 1 H), 4.70 (d, J = 11.7 Hz, 1 H), 4.48–4.45 (m, 2 H), 4.16–4.14 (m, 1 H), 3.88 (dd, J = 12.0, 2.7 Hz, 1 H), 3.80 (d, J = 11.7 Hz, 1 H), 1.90 (s, 3 H); ^{13}C NMR (150 MHz, $CDCl_3$) δ 170.5, 167.7, 138.3, 134.4, 131.7, 129.4, 128.5, 127.9, 123.7, 100.1, 77.4, 75.0, 73.4, 71.8, 68.3, 57.7, 20.9; HRMS (ESI) *m/z* calcd for $C_{46}H_{42}KN_2O_{14}$ $[M+K]^+$, 885.2268; found, 885.2217.

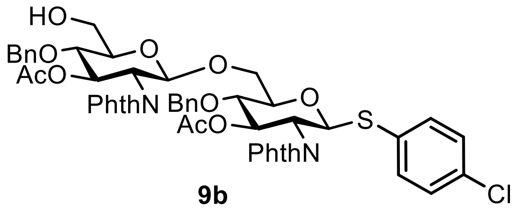

9b

Building block **6b** (226 mg, 0.400 mmol) afforded **9b** as in 13% isolated yield (25 mg, 0.0253 mmol). 4-Chlorophenyl-(3-*O*-acetyl-4-*O*-benzyl-2-deoxy-2-phthalimido-β-D-glucopyranosyl)-(1→6)-3-*O*-acetyl-4-*O*-benzyl-2-deoxy-2-phthalimido-1-thio-β-D-glucopyranoside (**9b**): TLC (hexane/EtOAc 1:1) R_f = 0.33; ^{1}H NMR (600 MHz, $CDCl_3$) δ 7.81–7.70 (m, 7 H), 7.61–7.58 (m, 2 H), 7.34–7.30 (m, 3 H), 7.29–7.27 (m, 4 H), 7.25–7.20 (m, 4 H), 7.02–7.01 (m, 2 H), 5.79 (dd, J = 10.5, 9.0 Hz, 1 H), 5.66 (dd, J = 10.2, 9.0 Hz, 1 H), 5.56 (d, J = 10.5 Hz, 1 H), 5.53 (d, J = 8.4 Hz, 1 H), 4.69 (d, J = 11.4 Hz, 1 H), 4.61 (d, J = 11.5 Hz, 1 H), 4.36 (d, J = 11.5 Hz, 1 H), 4.32 (d, J = 11.3 Hz, 1 H), 4.28 (dd, J = 10.8, 8.4 Hz, 1 H), 4.10 (*pseudo*–t, J = 10.5 Hz, 1 H), 4.05 (dd, J = 11.1, 1.5 Hz, 1 H), 3.95 (dd, J = 12.2, 2.6 Hz, 1 H), 3.84–3.78 (m, 2 H), 3.76 (dd, J = 11.2, 4.9 Hz, 1 H), 3.67–3.62 (m, 2 H), 3.52 (*pseudo*–t, J = 9.6 Hz, 1 H), 2.12–2.05 (m, 1 H), 1.79 (s, 3 H), 1.66 (s, 3 H); ^{13}C NMR (150 MHz, $CDCl_3$) δ 170.3, 170.1, 167.8, 167.3, 137.8, 137.5, 134.6, 134.5, 134.4, 134.3, 131.7, 131.2, 129.8, 129.2, 128.6, 128.5, 128.1, 127.9, 127.9, 127.5, 123.8, 123.7, 123.6, 123.6, 107.4, 98.3, 82.6, 78.5, 77.3, 77.1, 76.9, 76.6, 76.4, 75.4, 74.8, 74.7, 73.9, 73.2, 68.5, 61.8, 55.2, 53.9, 29.8, 20.7, 20.5; HRMS (ESI) *m/z* calcd for $C_{52}H_{47}ClKN_2O_{14}S$ $[M+K]^+$, 1029.2068; found, 1029.2040.

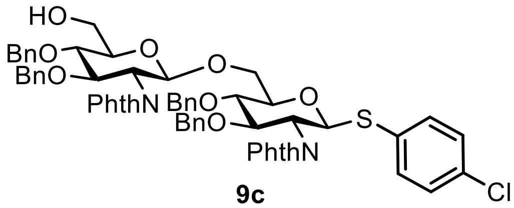

9c

Building block **6c** (246 mg, 0.400 mmol) afforded **9c** in 4% isolated yield (8.0 mg, 6.84 µmol)). 4-Chlorophenyl-(3,4-di-*O*-benzyl-2-deoxy-2-phthalimido-β-D-glucopyranosyl)-(1→6)-3,4-di-*O*-benzyl-2-deoxy-2-phthalimido-1-thio-β-D-glucopyranoside (**9c**): TLC (hexane/EtOAc 2:1) R_f = 0.23. ^{1}H NMR (600 MHz, $CDCl_3$) δ 7.76–7.62 (m, 4H), 7.56–7.48 (m, 3H), 7.36–7.33 (m, 4H), 7.32–7.28

(m, 1H), 7.24–7.17 (m, 6H), 7.09–7.07 (m, 2H), 7.00–6.99 (m, 2 H), 6.89–6.84 (m, 5H), 6.84–6.78 (m, 5H), 5.31 (d, *J* = 10.5 Hz, 1 H), 5.28 (d, *J* = 8.4 Hz, 1 H), 4.89 (d, *J* = 10.8 Hz, 1 H), 4.81 (d, *J* = 12.3 Hz, 1 H), 4.74 (d, *J* = 11.1 Hz, 1 H), 4.64 (d, *J* = 12.3 Hz, 1 H), 4.52 (d, *J* = 10.8 Hz, 1 H), 4.45 (d, *J* = 12.3 Hz, 1 H), 4.37–4.33 (m, 2 H), 4.28 (d, *J* = 12.3 Hz, 1 H), 4.21 (dd, *J* = 10.8, 8.7 Hz, 2 H), 4.03 (*pseudo*–t, *J* = 10.5 Hz, 1 H), 3.99 (dd, *J* = 10.8, 1.5 Hz, 1 H), 3.91 (d, *J* = 10.2 Hz, 1 H), 3.78–3.72 (m, 2 H), 3.67 (dd, *J* = 11.1, 5.1 Hz, 1 H), 3.56–3.51 (m, 2 H), 3.44–3.40 (m, 1 H), 2.09–2.07 (m, 1 H); ^{13}C NMR (150 MHz, $CDCl_3$) δ 167.23, 167.21, 138.0, 137.9, 137.7, 137.5, 134.3, 134.0, 133.98, 133.97, 133.95, 133.9, 131.5, 131.49, 130.2, 129.1, 128.7, 128.6, 128.5, 128.4, 128.2, 128.15, 128.1, 128.07, 128.04, 128.01, 127.96, 127.5, 123.5, 123.48, 123.45, 123.4, 98.4, 82.9, 80.1, 79.6, 79.2, 79.1, 78.6, 75.5, 75.2, 75.0, 68.2, 62.0, 55.8, 54.6, 29.8; HRMS (ESI) *m/z* calcd for $C_{62}H_{55}ClNaN_2O_{12}S$ $[M+Na]^+$, 1109.3056; found, 1109.3041.

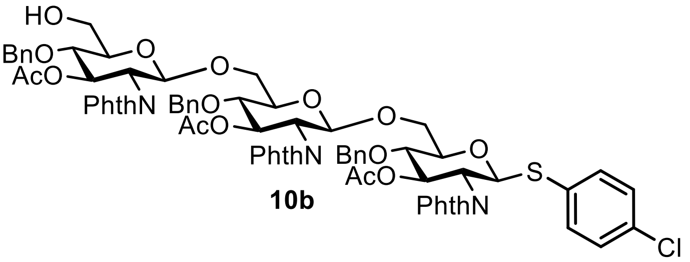

10b

Building block **6b** (226 mg, 0.400 mmol) afforded **10b** in 6% isolated yield (11 mg, 7.80 μmol). 4-Chlorophenyl-(3-*O*-acetyl-4-*O*-benzyl-2-deoxy-2-phthalimido-β-ᴅ-glucopyranosyl)-(1→6)-(3-*O*-acetyl-4-*O*-benzyl-2-deoxy-2-phthalimido-β-ᴅ-glucopyranosyl)-(1→6)-3-*O*-acetyl-4-*O*-benzyl-2-deoxy-2-phthalimido-1-thio-β-ᴅ-glucopyranoside (**10b**): TLC (hexane/EtOAc 1:1) R_f = 0.23; ^{1}H NMR (600 MHz, $CDCl_3$) δ 7.85–7.80 (m, 2 H), 7.76–7.68 (m, 6 H), 7.62–7.57 (m, 4 H), 7.31–7.26 (m, 7 H), 7.24–7.22 (m, 2 H), 7.21–7.18 (m, 6 H), 7.02–6.96 (m, 4 H), 5.78 (dd, *J* = 10.8, 9.0 Hz, 1 H), 5.66–5.62 (m, 2 H), 5.55 (d, *J* = 8.4 Hz, 1 H), 5.54 (d, *J* = 10.5 Hz, 1 H), 5.45 (d, *J* = 8.4 Hz, 1 H), 4.67 (d, *J* = 11.7 Hz, 1 H), 4.61 (d, *J* = 11.7 Hz, 1 H), 4.36–4.30 (m, 3 H), 4.28 (d, *J* = 11.2 Hz, 1 H), 4.24 (d, *J* = 11.3 Hz, 1 H), 4.21 (dd, *J* = 10.7, 8.5 Hz, 1 H), 4.13–4.09 (m, 1 H), 4.06–4.01 (m, 2 H), 3.96 (ddd, *J* = 12.1, 4.9, 2.2 Hz, 1 H), 3.87 (dd, *J* = 11.4, 4.2 Hz, 1 H), 3.80–3.75 (m, 2 H), 3.70 (dd, *J* = 11.4, 4.8 Hz, 1 H), 3.67–3.64 (m, 2 H), 3.63–3.57 (m, 2 H), 3.45 (*pseudo*–t, *J* = 9.6 Hz, 1 H), 2.32 (dd, *J* = 8.5, 5.2 Hz, 1 H), 1.76 (s, 3 H), 1.70 (s, 3 H), 1.62 (s, 3 H); ^{13}C NMR (150 MHz, $CDCl_3$) δ 170.3, 170.1, 167.9, 167.3, 137.9, 137.6, 134.9, 134.7, 134.5, 134.3, 134.2, 134.1, 131.8, 131.2, 129.3, 128.6, 128.5, 128.4, 128.4, 127.9, 127.8, 127.6, 127.5, 123.8, 123.7, 123.6, 98.2, 82.0, 78.3, 76.7, 76.5, 76.4, 75.5, 74.8, 74.7, 74.66, 74.65, 73.8, 73.3, 73.25, 73.2, 68.2, 68.16, 61.6, 60.5, 55.3, 55.0, 31.7, 22.7, 21.2, 20.7, 20.6, 20.5, 14.3, 14.2; HRMS (ESI) *m/z* calculated for $C_{75}H_{68}ClNaN_3O_{21}S$ $[M+Na]^+$, 1436.3647; found, 1436.3628.

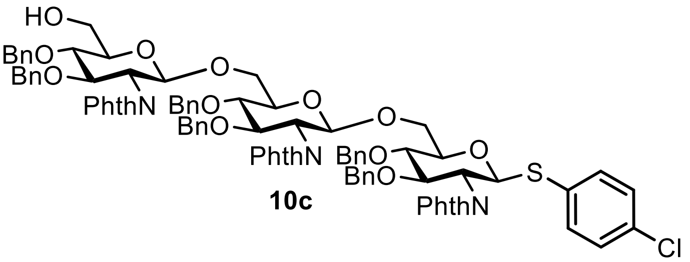

10c

Building block **6c** (246 mg, 0.40 mmol) afforded **10c** in 2% isolated yield (5.0 mg, 3.21 μmol)).
4-Chlorophenyl-(3,4-di-*O*-benzyl-2-deoxy-2-phthalimido-β-ᴅ-glucopyranosyl)-(1→6)-(3,4-di-*O*-benzyl-2-deoxy-2-phthalimido-β-ᴅ-glucopyranosyl)-(1→6)-3,4-di-*O*-benzyl-2-deoxy-2-phthalimido-1-thio-β-ᴅ-glucopyranoside (**10c**): TLC (hexane/EtOAc 1:1) R_f = 0.50; ^{1}H NMR (600 MHz, $CDCl_3$) δ7.78–7.74 (m, 1 H), 7.72–7.62 (m, 5 H), 7.56–7.54 (m, 1 H), 7.53–7.48 (m, 2 H), 7.34–7.32 (m, 4 H), 7.30–7.26 (m, 4 H), 7.23–7.20 (m, 6 H), 7.20–7.17 (m, 4 H), 7.10–7.08 (m, 2 H), 7.07–7.04 (m, 2 H), 6.97–6.95 (m, 2 H), 6.87–6.85 (m, 2 H), 6.93–6.90 (m, 2 H), 6.97–6.77 (m, 9 H), 5.32 (d, *J* = 8.4 Hz, 1 H), 5.25 (d, *J* = 10.4 Hz, 1 H), 5.16 (d, *J* = 8.2 Hz, 1 H), 4.85 (d, *J* = 10.9 Hz, 1 H), 4.77 (d, *J* = 12.1 Hz, 1 H), 4.72 (d, *J* = 11.0 Hz, 1 H), 4.66 (d, *J* = 12.2 Hz, 1 H), 4.59 (d, *J* = 12.1 Hz, 1 H), 4.47 (d, *J* = 10.6 Hz, 1 H), 4.44–4.38 (m, 2 H), 4.37 (d, *J* = 12.0 Hz, 1 H), 4.35 (dd, *J* = 10.7, 8.6 Hz, 1 H), 4.33–4.30 (m, 2 H), 4.28–4.24 (m, 3 H), 4.21–4.12 (m, 4 H), 4.06 (dd, *J* = 10.9, 1.5 Hz, 1 H), 3.98–3.91 (m, 3 H), 3.78 (dd, *J* = 11.3, 4.5 Hz, 1 H), 3.72 (*pseudo*-t, *J* = 9.1 Hz, 1 H), 3.60–3.55 (m, 2 H), 3.50 (dd, *J* = 10.0, 3.8 Hz, 1 H), 3.42 (dd, *J* = 10.3, 3.1 Hz, 1 H), 3.37 (*pseudo*-t, *J* = 9.6 Hz, 1 H), 2.45–2.37 (m, 1 H); ^{13}C NMR (150 MHz, $CDCl_3$) δ 168.2, 168.0, 137.6, 137.4, 134.8, 134.6, 134.3, 134.2, 133.9, 131.8, 131.4, 129.2, 129.14, 129.1, 128.8, 128.7, 128.6, 128.59, 128.57, 128.5, 128.4, 128.3, 128.25, 128.2, 128.15, 128.13, 128.10, 128.09, 128.05, 128.04, 128.02, 127.99, 127.93, 127.91, 127.9, 127.6, 127.4, 127.36, 123.9, 123.6, 123.4, 102.2, 101.1, 85.4, 79.0, 76.5, 75.1, 74.8, 72.5, 71.8, 70.4, 70.3, 68.8, 68.2, 64.7, 57.8, 51.9, 29.8, 26.5; HRMS (ESI) *m/z* calcd for $C_{90}H_{80}ClKN_3O_{18}S$ $[M+K]^+$, 1596.4478; found, 1596.4441.

4. General procedure for cyclic oligoglucosamine with a 2,3-oxazolidinone group

Synthesis of cyclobis[(1→6)-2-acetamido-4-*O*-benzyl-2,3-*N*,*O*-carbonyl-2-deoxy-β-ᴅ-glucopyranosyl] (**16**)

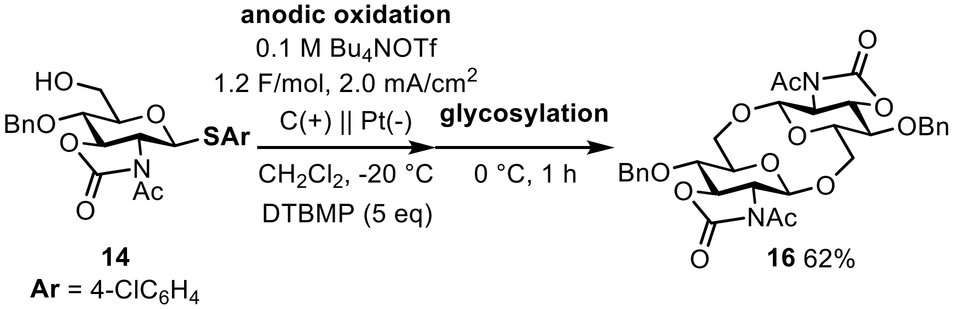

The electrochemical one–pot synthesis of cyclic disaccharide **16** was conducted with an H–type divided cell (4G glass filter). The cell had a carbon felt anode (Nippon Carbon JF–20–p7) and platinum square plate (20 mm × 20 mm). Building block **14** (0.400 mmol, 186 mg), Bu_4NOTf (1.00 mmol, 392 mg), 2,6-di-*tert*-butyl-4-methylpyridine (DTBMP) (2.0 mmol, 411 mg), and CH_2Cl_2 (10 mL) were added to the anodic chamber. TfOH (0.400 mmol, 35.2 μL), Bu_4NOTf (1.00 mmol, 392 mg), and CH_2Cl_2 (10 mL) were added to the cathodic chamber. As the initiation phase of reaction, the constant current (8.0 mA (current density: 2.0 mA/cm^2), 18~22 V (electrode distance: 4.5 cm)) was employed at –20 °C with magnetic stirring until 1.2 F/mol of electricity was consumed. After the electrolysis, the reaction temperature was raised from –20 °C to 0 °C as glycosylation phase. After glycosylation, Et_3N (0.5 mL) was added to both of the chambers. The solution was collected, and the solvent was removed under reduced pressure. The mixture was dissolved in EtOAc and washed with H_2O to remove electrolyte. It was further washed with 1 N HCl (aq) to remove excessive DTBMP. The solution was dried over anhydrous Na_2SO_4, and the solvent was removed under reduced pressure. The crude product was purified with preparative GPC to afford cyclic oligosaccharide **16** (0.125 mmol, 79.7 mg, 62%) as a white solid.

Cyclobis[(1→6)-2-acetamido-4-*O*-benzyl-2,3-*N*,*O*-carbonyl-2-deoxy-β-ᴅ-glucopyranosyl] (**16**): TLC (eluent: hexane/EtOAc 2:1) R_f = 0.31; ^{1}H NMR ($CDCl_3$, 600 MHz) d 7.38–7.35 (m, 4 H), 7.33–7.30 (m, 1 H), 5.24 (d, *J* = 5.7 Hz, 1 H), 4.89 (d, *J* = 11.4 Hz, 1 H) 4.62 (d, *J* = 11.2 Hz, 1 H), 4.31 (dd, *J* = 12.5, 6.6 Hz, 1 H), 4.18 (dd, *J* = 9.7, 4.3 Hz, 1 H), 4.16–4.13 (m, 1 H), 4.01 (dd, *J* = 10.9, 2.5 Hz, 1 H), 3.94 (dd, *J* = 12.5, 5.8 Hz, 1 H), 3.59 (dd, *J* = 11.0, 1.5 Hz, 1H), 2.53 (s, 3 H); ^{13}C NMR ($CDCl_3$, 150 MHz) d 170.6, 153.7, 137.3, 128.5, 128.0, 127.9, 97.0, 81.7, 73.1, 63.8, 62.0, 24.5; HRMS (ESI) *m/z* calcd for $C_{32}H_{34}KN_2O_{12}$; [M+K]$^+$, 677.1744, found 677.1735.

5. General procedure for cyclic oligoglucosamines with an azido group

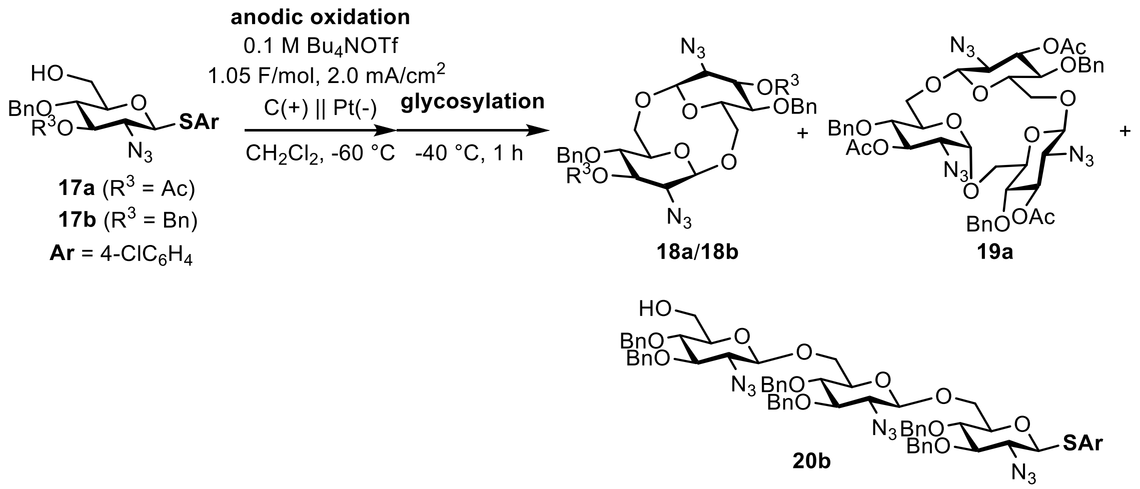

The electrochemical polymerization synthesis of cyclic oligosaccharides **18** and **19** and linear oligosaccharide **20** was carried out by an H–type divided cell (4G glass filter). The cell had a carbon felt anode (Nippon Carbon JF–20–P7) and platinum square plate (20 mm × 20 mm). Building block **17** (0.40 mmol), Bu_4NOTf (1.00 mmol), and CH_2Cl_2 (10 mL) were added to the anodic chamber. TfOH (0.4 mmol), Bu_4NOTf (1.00 mmol), and CH_2Cl_2 (10 mL) were added to the cathodic chamber. The constant current (8 mA (current density: 2.0 mA/cm^2), (electrode distance: 4.5 cm)) was employed at –60 °C with magnetic stirring until the electricity was consumed. After the electrolysis, the reaction was kept stirring at –40 °C for 1 h. After that, Et_3N (0.3 mL) was added to both chambers. The solution in both chambers was collected in eggplant flask, and the solvent was removed under reduced pressure. The mixture was dissolved in EtOAc and washed with H_2O to remove electrolyte. The solution was dried over anhydrous Na_2SO_4, filtered, and the solution was removed under reduced pressure. The crude product was purified with preparative GPC to afford cyclic oligosaccharides **18** and **19** and linear trisaccharide **20b**.

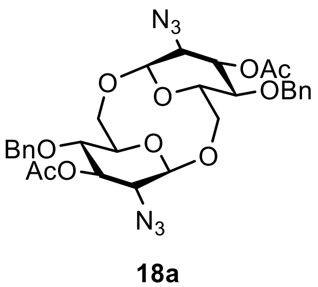

18a

Building block **17a** (185 mg, 0.40 mmol) afforded **18a** in 49% isolated yield (63 mg, 0.987mmol). Cyclobis[(1→6)-3-*O*-acetyl-2-azido-4-*O*-benzyl-2-deoxy-2-β-D-glucopyranosyl] (**18a**): TLC (hexane/EtOAc 3:1) R_f = 0.28; ^{1}H NMR (600 MHz, $CDCl_3$) δ 7.35–7.29 (m, 5 H), 5.05 (dd, J = 9.0,

3.6 Hz, 1 H), 4.72 (d, J = 1.8 Hz, 1 H), 4.69 (d, J = 11.6 Hz, 1 H), 4.61 (d, J = 11.5 Hz, 1 H), 4.44 (dd, J = 10.2, 9.6 Hz, 1 H), 4.11 (dd, J = 12.3, 1.2 Hz, 1 H), 3.82–3.79 (m, 1 H), 3.71 (dd, J = 12.6, 1.2 Hz, 1 H), 3.65 (dd, J = 3.6, 1.8 Hz, 1 H), 1.96 (s, 3 H); ^{13}C NMR (150 MHz, $CDCl_3$) δ 170.2, 138.1, 128.6, 128.2, 128.0, 100.3, 75.7, 74.7, 74.5, 73.7, 70.9, 65.3, 21.0; HRMS (ESI) *m/z* calcd for $C_{30}H_{34}KN_6O_{10}$ $[M+K]^+$, 677.1968; found, 677.1933.

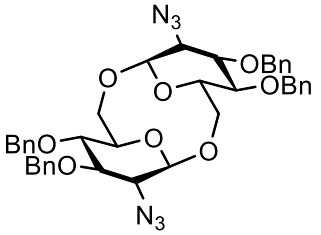

18b

Building block **17b** (205 mg, 0.40 mmol) afforded **18b** in 14% isolated yield (21 mg, 0.0281 mmol). Cyclobis[(1→6)-2-azido-3,4-di-*O*-benzyl-2-deoxy-2-β-D-glucopyranosyl] (**18b**): TLC (hexane/EtOAc 3:1) R_f = 0.63; ^{1}H NMR (600 MHz, $CDCl_3$) δ 7.36–7.28 (m, 10 H), 4.86 (d, J = 11.3 Hz, 1 H), 4.84 (d, J = 11.0 Hz, 1 H), 4.78 (d, J = 11.1 Hz, 1 H), 4.67 (d, J = 11.4 Hz, 1 H), 4.58–4.57 (m, 1 H), 4.20 (*pseudo*–t, J = 9.6 Hz, 1 H), 3.92 (dd, J = 12.0, 2.4 Hz, 1 H), 3.76 (dd, J = 9.6, 1.8 Hz, 1 H), 3.67–3.62 (m, 2 H), 3.50 (dd, J = 6.6, 1.2 Hz, 1 H), 1.43 (dd, J = 15.0, 7.5 Hz, 2 H); ^{13}C NMR (150 MHz, $CDCl_3$) δ 138.2, 137.8, 128.54, 128.49, 128.13, 128.09, 127.98, 127.97, 100.9, 81.7, 77.0, 75.0, 74.5, 74.2, 69.2, 67.7, 58.9, 24.0, 19.7, 13.7; HRMS (ESI) *m/z* calcd for $C_{40}H_{42}KN_6O_8$ $[M+K]^+$, 773.2696; found, 773.2650.

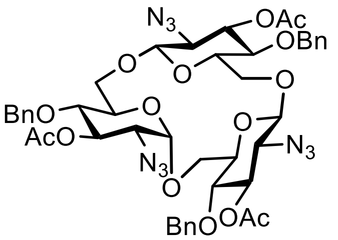

19a

Building block **17a** (185 mg, 0.40 mmol) afforded **19a** in 16% isolated yield (19 mg, 0.0208 mmol). Cyclotris[(1→6)-3-*O*-acetyl-2-azido-4-*O*-benzyl-2-deoxy-2-α,β,β-D-glucopyranosyl] (**19a**): TLC (hexane/EtOAc 2:1) R_f = 0.32; ^{1}H NMR (600 MHz, $CDCl_3$) δ 7.37–7.28 (m, 10 H), 7.28–7.25 (m, 2 H), 7.22–7.21 (m, 3 H), 5.61 (dd, J = 10.8, 9.0 Hz, 1 H), 5.10 (dd, J = 10.8, 9.6 Hz, 1 H), 4.95 (*pseudo*–t, J = 10.2 Hz, 1 H), 4.80 (d, J = 3.6 Hz, 1 H), 4.72 (d, J = 11.4 Hz, 1 H), 4.63 (d, J = 11.4 Hz, 1 H), 4.61 (d, J = 11.4 Hz, 2 H), 4.53 (d, J = 12.0 Hz, 1 H), 4.52 (d, J = 7.8 Hz, 1 H), 4.51 (d, J = 11.4 Hz, 1 H), 4.33 (dd, J = 12.6, 1.8 Hz, 1 H), 4.22 (d, J = 7.8 Hz, 1 H), 4.03 (dd, J = 12.6, 8.4 Hz, 1 H), 3.91–

3.89 (m, 1 H), 3.81–3.77 (m, 3 H), 3.75 (*pseudo*-t, J = 9.6 Hz, 1 H), 3.70 (ddd, J = 9.0, 6.6, 1.2 Hz, 1 H), 3.45 (dd, J = 10.2, 7.8 Hz, 1 H), 3.44 (dd, J = 10.3, 6.0 Hz, 1H), 3.29 (*pseudo*–t, J = 9.6 Hz, 1H), 3.18–3.15 (m, 1 H), 3.15 (*pseudo*-t, J = 9.6 Hz, 1 H), 3.06 (dd, J = 10.8, 3.6 Hz, 1 H), 2.08 (s, 3 H), 2.06 (s, 3 H), 1.96 (s, 3 H); ^{13}C NMR (150 MHz, $CDCl_3$) δ 170.0, 169.7, 137.9, 137.2, 137.15, 128.8, 128.7, 128.5, 128.48, 128.3, 128.0, 127.9, 127.74, 127.7, 105.7, 101.3, 97.9, 77.7, 76.4, 75.3, 75.28, 74.9, 74.3, 74.2, 74.0, 73.9, 73.5, 71.8, 71.6, 69.6, 68.8, 64.9, 63.7, 61.2, 21.1, 21.0, 20.96, 14.3; HRMS (ESI) *m/z* calcd for $C_{45}H_{51}KN_9O_{15}$ $[M+K]^+$, 996.3136; found, 996.3195.

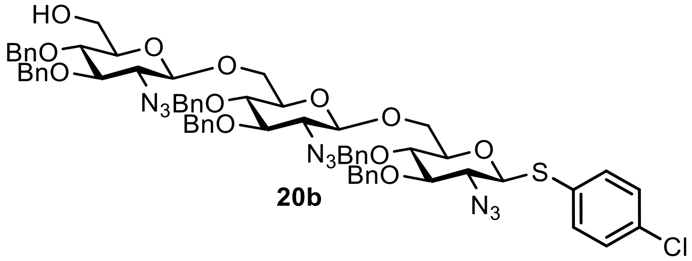

20b

Building block **17b** (205 mg, 0.40 mmol) afforded **20b** in 13% isolated yield (21 mg, 0.0171 mmol). 4-Chlorophenyl-(2-azido-2-deoxy-3,4-di-*O*-benzyl-β-D-glucopyranosyl)-(1→6)-(2-azido-2-deoxy-3,4-di-*O*-benzyl-β-D-glucopyranosyl)-(1→6)-2-azido-2-deoxy-3,4-di-*O*-benzyl-1-thio-β-D-glucopyranoside (**20b**): TLC (hexane/EtOAc 2:1) R_f = 0.40; ^{1}H NMR (600 MHz, $CDCl_3$) δ 7.55–7.53 (m, 2 H), 7.37–7.34 (m, 5 H), 7.33–7.30 (m, 15 H), 7.29–7.26 (m, 12 H), 4.64–4.61 (m, 3 H), 4.42 (d, J = 10.2 Hz, 1 H), 4.29–4.26 (m, 2 H), 4.16 (dd, J = 11.4, 1.8 Hz, 1 H), 4.04 (dd, J = 11.4, 1.5 Hz, 1 H), 3.79 (dd, J = 11.4, 5.1 Hz, 1 H), 3.66–3.62 (m, 2 H), 3.54–3.45 (m, 4 H), 3.43–3.40 (m, 1 H), 3.38–3.36 (m, 1 H), 3.31 (dd, J = 10.2, 9.3 Hz, 1 H), 3.21 (*pseudo*–t, J = 8.7 Hz, 4 H), 1.90 (dd, J = 7.7, 6.0 Hz, 1H);; ^{13}C NMR (150 MHz, $CDCl_3$) δ 137.9, 137.82, 137.79, 137.6, 135.0, 129.3, 128.82, 128.77, 128.66, 128.62, 128.61, 128.54, 128.49, 128.3, 128.21, 128.19, 128.18, 128.15, 128.13, 128.09, 128.08, 128.05, 128.02, 127.99, 127.98, 129.92, 127.9, 127.7, 102.6, 102.4, 86.0, 85.0, 83.2, 82.9, 78.9, 77.8, 77.6, 75.9, 75.8, 75.5, 75.4, 75.13, 75.10, 75.09, 74.8, 66.5, 66.4, 65.1, 61.7, 58.9, 24.0, 19.8, 13.7; HRMS (ESI) *m/z* calcd for $C_{66}H_{68}ClNaN_9O_{12}S$ $[M+Na]^+$, 1268.4289; found, 1268.4249.

6. Molecular orbital calculations for the anhydrosugar 15

The molecular orbital calculations were carried out with 1,6-anhydro-2-acetamido-4-*O*-benzyl-2,3-*N*,*O*-carbonyl-2-deoxy-β-D-glucopyranoside (**15**) at the B3LYP/6-31G(d) level using the Gaussian 16, Revision C.02.[8] Geometries were fully optimized. All the optimized structures were local minima according to the vibration analysis. Cartesian coordinates of computationally characterized species are as follows:

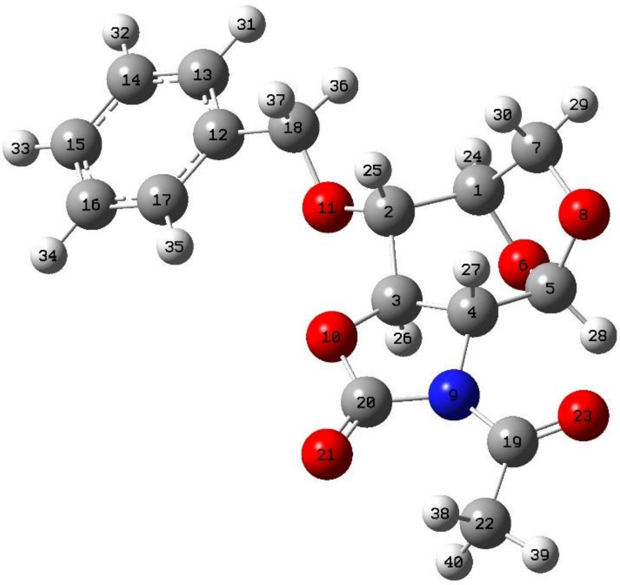

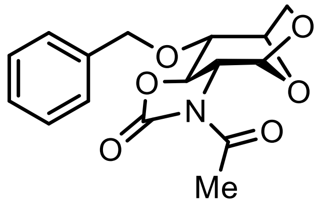

15

(white H, black C, blue N, red O)

center	atomic	atomic		coordinates (Å)	
number	number	type	X	Y	Z
1	6	0	-0.16492	2.131337	0.2187
2	6	0	0.274667	0.754509	-0.42251
3	6	0	-0.7847	-0.2157	0.051899
4	6	0	-2.17365	0.346913	-0.18699
5	6	0	-2.30435	1.679611	0.613134
6	8	0	-1.08166	1.900996	1.306095
7	6	0	-1.02417	3.036293	-0.6985
8	8	0	-2.37958	2.739594	-0.32505
9	7	0	-2.93857	-0.87696	0.077304
10	8	0	-0.86217	-1.52716	-0.54015
11	8	0	1.526591	0.308336	0.050603
12	6	0	3.895483	0.079124	-0.22469
13	6	0	5.10813	0.755448	-0.05983
14	6	0	6.269702	0.058067	0.277473

15	6	0	6.224147	-1.32373	0.464531
16	6	0	5.013341	-2.00364	0.310461
17	6	0	3.855851	-1.30808	-0.03621
18	6	0	2.654427	0.828476	-0.64793
19	6	0	-4.33893	-0.85859	0.207606
20	6	0	-2.13822	-1.97578	-0.3258
21	8	0	-2.47812	-3.11809	-0.48533
22	6	0	-5.05385	-2.18061	0.34572
23	8	0	-4.90638	0.219384	0.233441
24	1	0	0.711775	2.634626	0.628949
25	1	0	0.285138	0.831605	-1.52156
26	1	0	-0.63025	-0.35481	1.125877
27	1	0	-2.31666	0.608144	-1.2439
28	1	0	-3.14116	1.728496	1.304974
29	1	0	-0.83681	4.09738	-0.5056
30	1	0	-0.86848	2.837402	-1.76574
31	1	0	5.146116	1.834702	-0.19313
32	1	0	7.205483	0.596154	0.403168
33	1	0	7.125442	-1.86806	0.733453
34	1	0	4.970403	-3.07924	0.460004
35	1	0	2.911867	-1.83238	-0.14819
36	1	0	2.778026	1.905583	-0.45278
37	1	0	2.487933	0.714053	-1.73309
38	1	0	-4.98025	-2.75917	-0.57938
39	1	0	-6.09876	-1.96667	0.57474
40	1	0	-4.60718	-2.79555	1.131953

7. References

1. Sugiyama, S.; Diakur, J. M. *Org. Lett*. **2000**, *2*, 2713.
2. Yano, K.; Itoh, T.; Nokami, T. *Carbohydr. Res.* **2020**, *492*, 109018.
3. Rahman, A. M.; Takahashi, S.; Sasaki, N.; Itoh, T.; Ohnuma, T.; Nokami, T. *Electrochemistry* **2023**, *91*, 112013.
4. Chun, Y.; Yan, S.; Li, X.; Ding, N.; Zhang, W.; Wang, P.; Li, M.; Li, Y. *Tetrahedron Lett.* **2011**, *52*, 6196.
5. Nokami, T.; Hayashi, R.; Saigusa, Y.; Shimizu, A.; Liu, C.-Y.; Mong. T. K.; Yoshida, J. *Org. Lett.* **2013**, *15*, 4520.
6. Garcia, J.; Vilarrasa, J.; Bordas, X.; Banaszek, A. *Tetrahedron Lett*. **1986**, *27*, 639.
7. Gening, M. L.; Titov, D. V.; Grachev, A. A.; Gerbst, A. G.; Yudina, O. N.; Shashkov, A. S.; Chizhov, A. O. Tsvetkov, Y. E.; Nifantiev, N. E. *Eur. J. Org. Chem*. **2010**, *13*, 2465.
8. Gaussian 16, Revision C.02, Frisch, M. J.; Trucks, G. W.; Schlegel, H. B.; Scuseria, G. E.; Robb, M. A.; Cheeseman, J. R.; Scalmani, G.; Barone, V.; Petersson, G. A.; Nakatsuji, H.; Li, X.; Caricato, M.; Marenich, A. V.; Bloino, J.; Janesko, B. G.; Gomperts, R.; Mennucci, B.; Hratchian, H. P.; Ortiz, J. V.; Izmaylov, A. F.; Sonnenberg, J. L.; Williams-Young, D.; Ding, F.; Lipparini, F.; Egidi, F.; Goings, J.; Peng, B.; Petrone, A.; Henderson, T.; Ranasinghe, D.; Zakrzewski, V. G.; Gao, J.; Rega, N.; Zheng, G.; Liang, W.; Hada, M.; Ehara, M.; Toyota, K.; Fukuda, R.; Hasegawa, J.; Ishida, M.; Nakajima, T.; Honda, Y.; Kitao, O.; Nakai, H.; Vreven, T.; Throssell, K.; Montgomery, Jr., J. A.; Peralta, J. E.; Ogliaro, F.; Bearpark, M. J.; Heyd, J. J.; Brothers, E. N.; Kudin, K. N.; Staroverov, V. N.; Keith, T. A.; Kobayashi, R.; Normand, J.; Raghavachari, K.; Rendell, A. P.; Burant, J. C.; Iyengar, S. S.; Tomasi, J.; Cossi, M.; Millam, J. M.; Klene, M.; Adamo, C.; Cammi, R.; Ochterski, J. W.; Martin, R. L.; Morokuma, K.; Farkas, O.; Foresman, J. B. and Fox, D. J., Gaussian, Inc., Wallingford CT, **2016**.

8· ^{1}H and ^{13}C NMR spectra of monosaccharides and oligosaccharides

^{1}H NMR

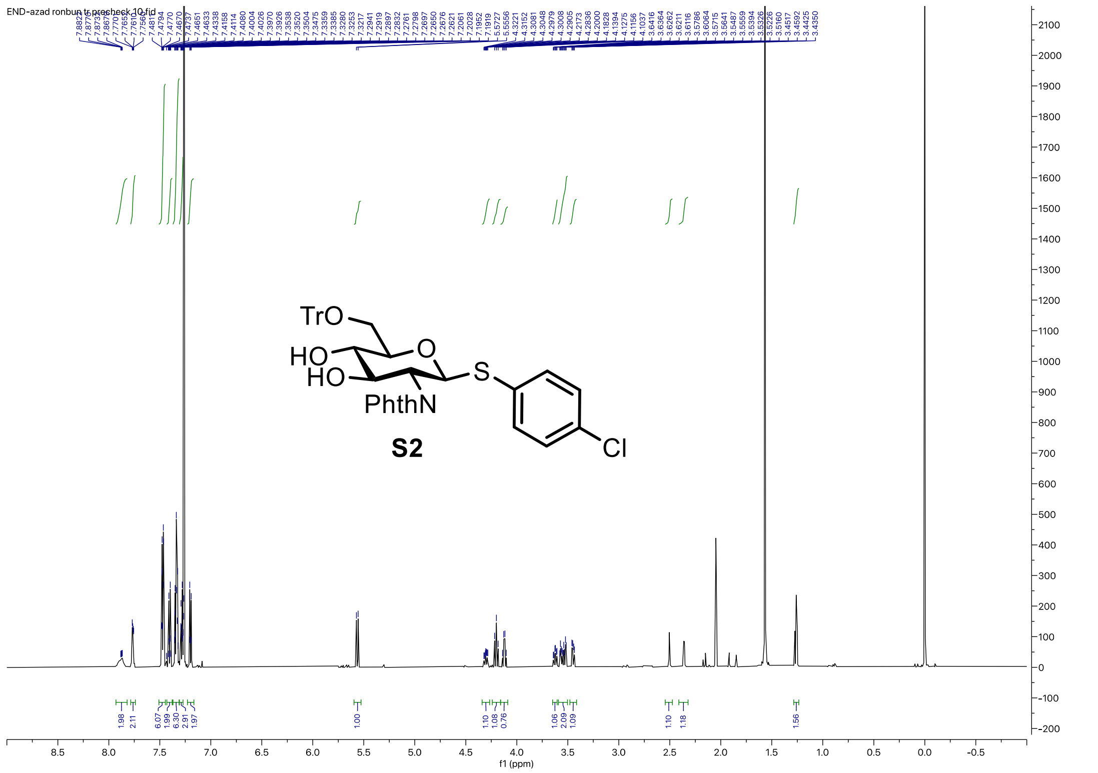

^{13}C NMR

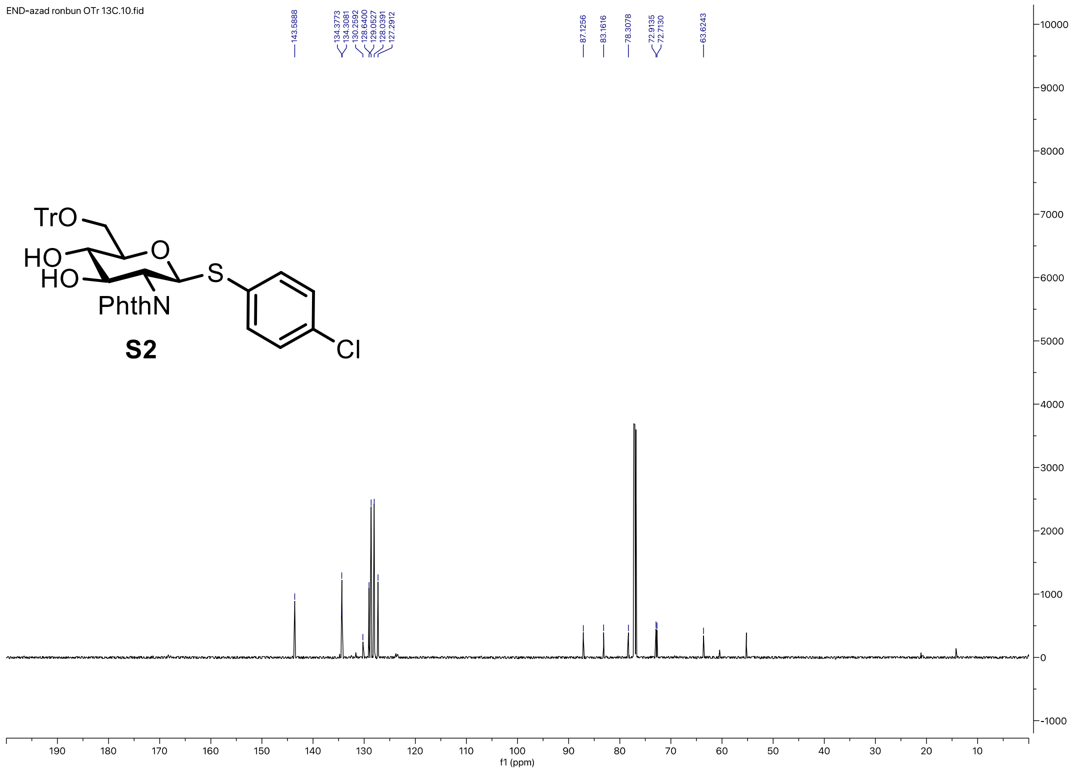

^{1}H NMR

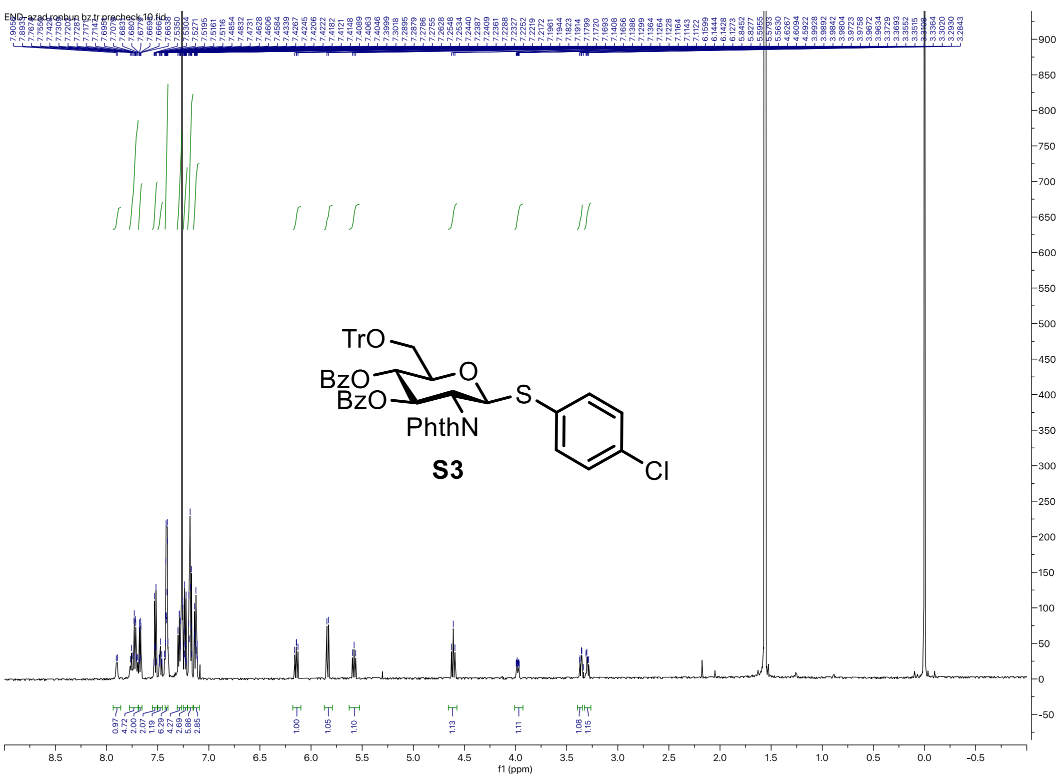

^{13}C NMR

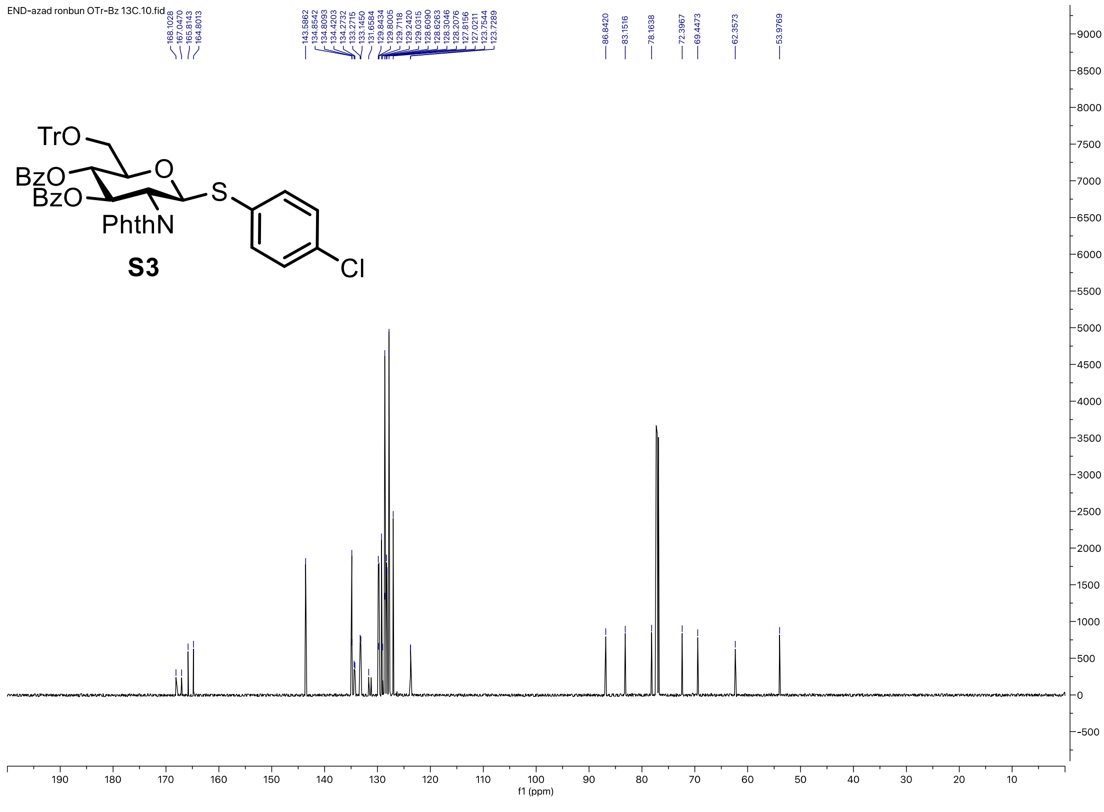

^{1}H NMR

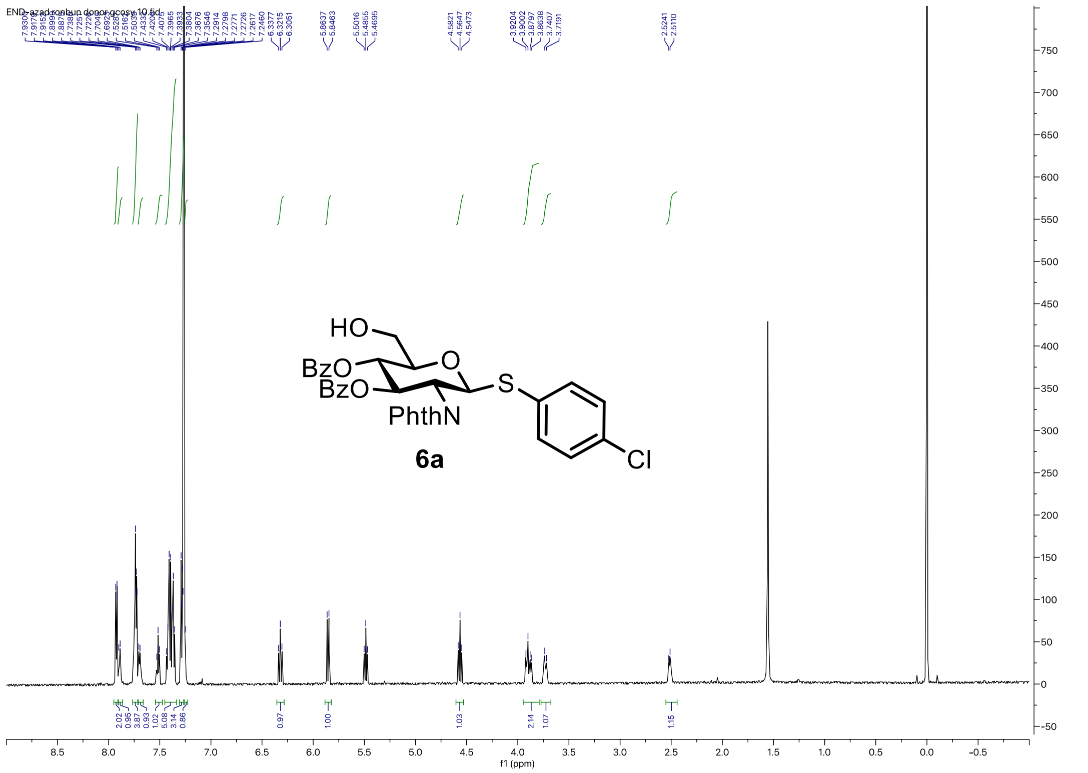

^{13}C NMR

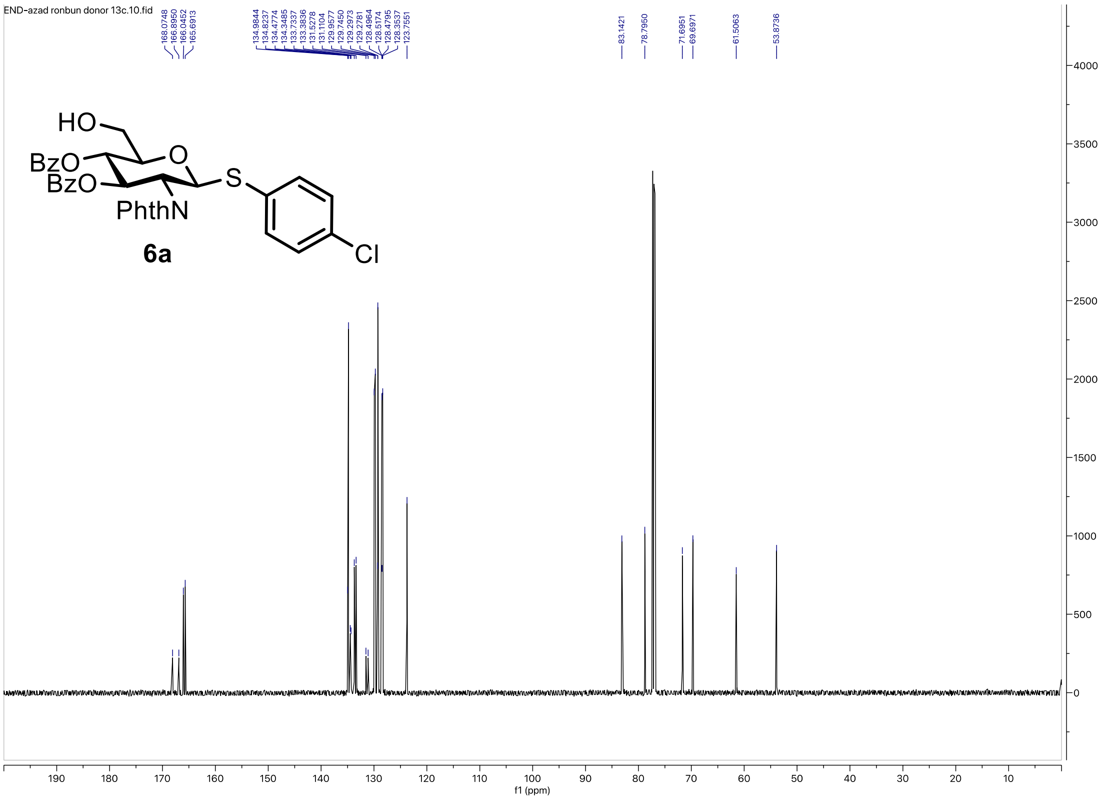

^{1}H NMR

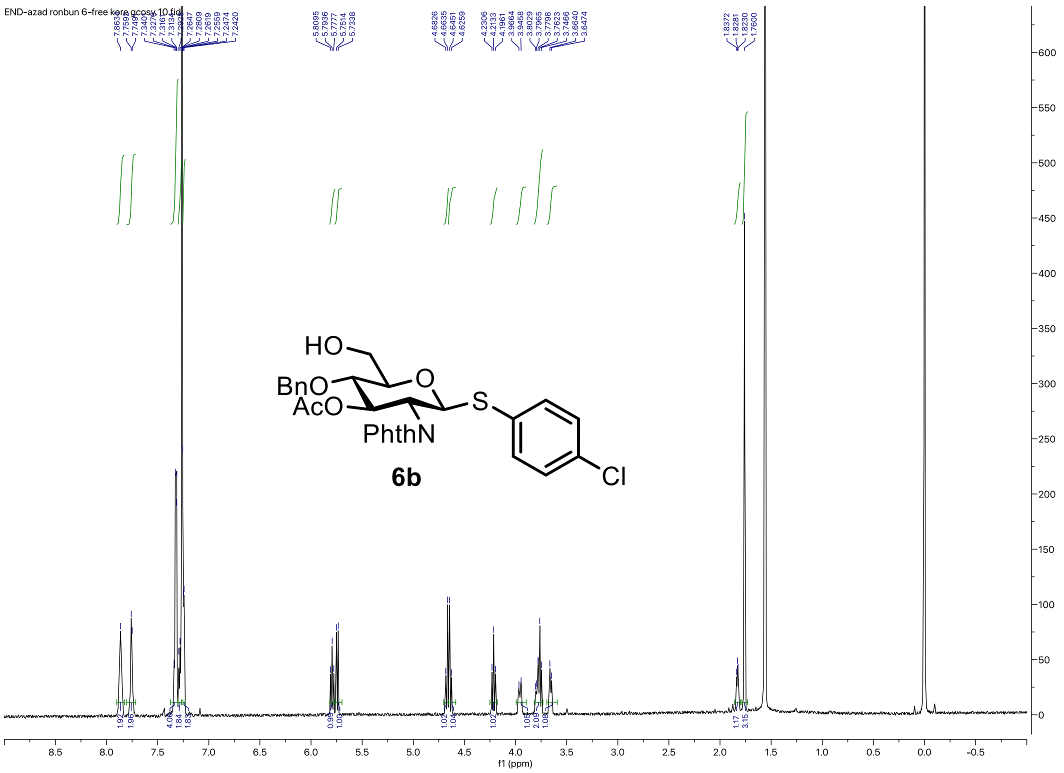

^{13}C NMR

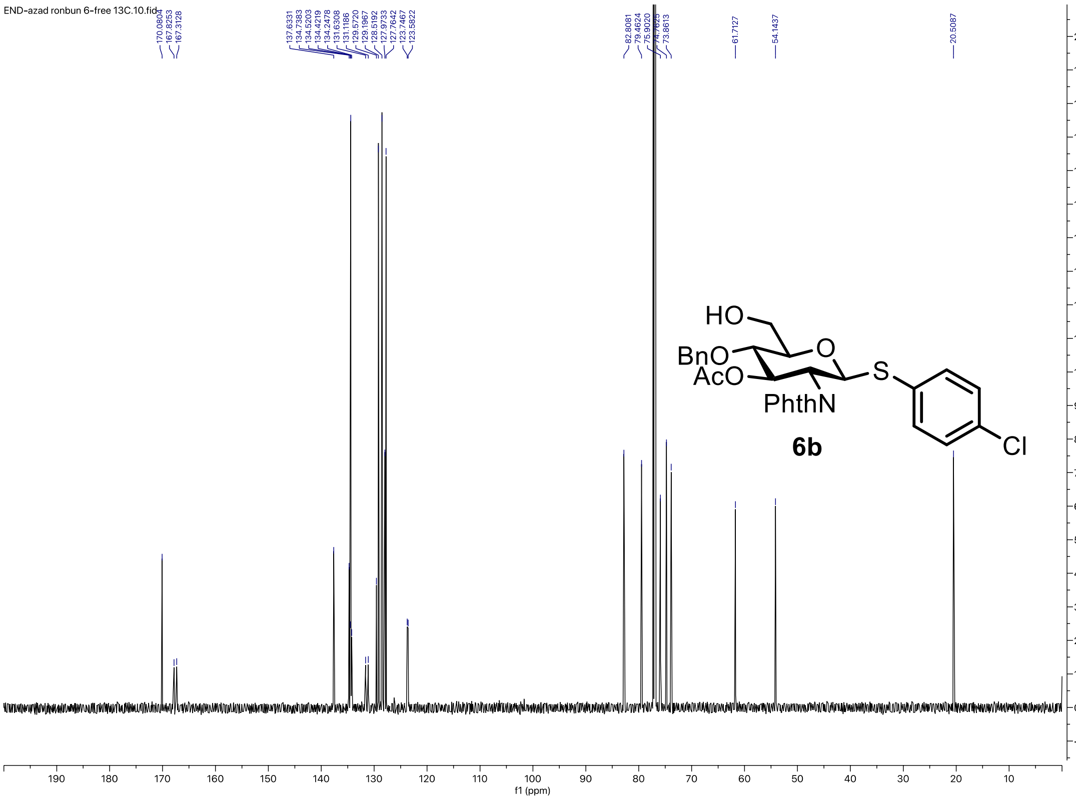

^{1}H NMR

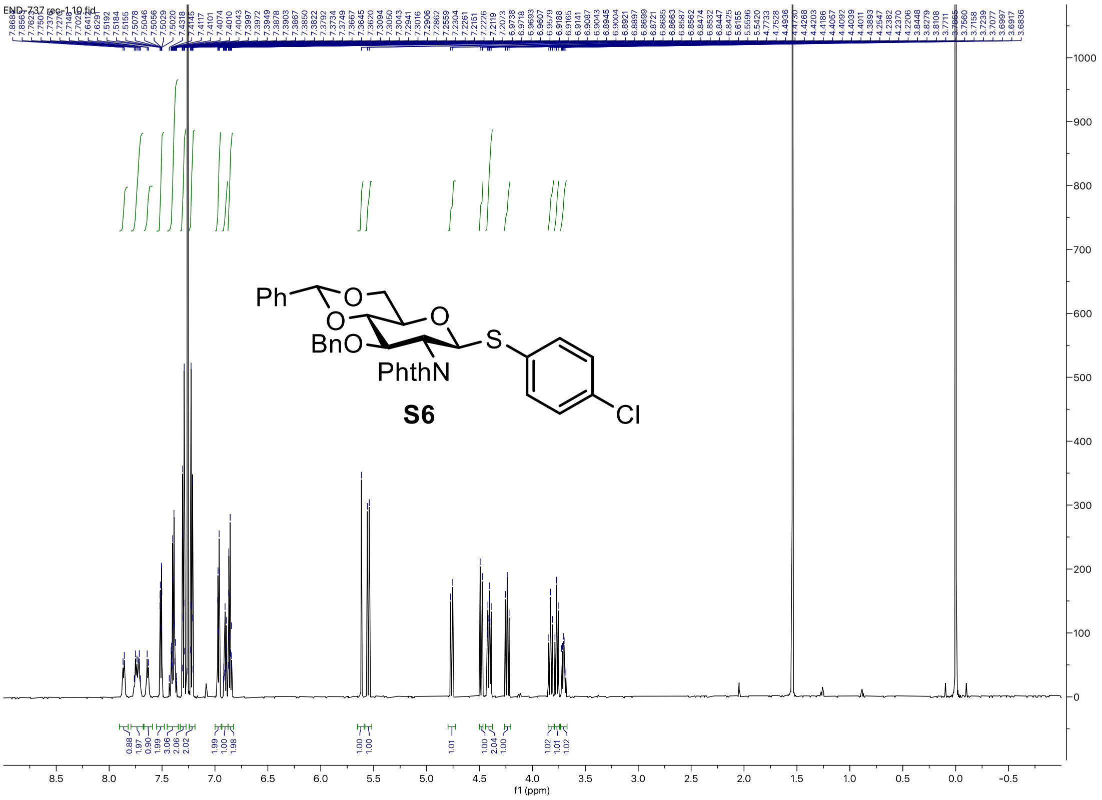

^{13}C NMR

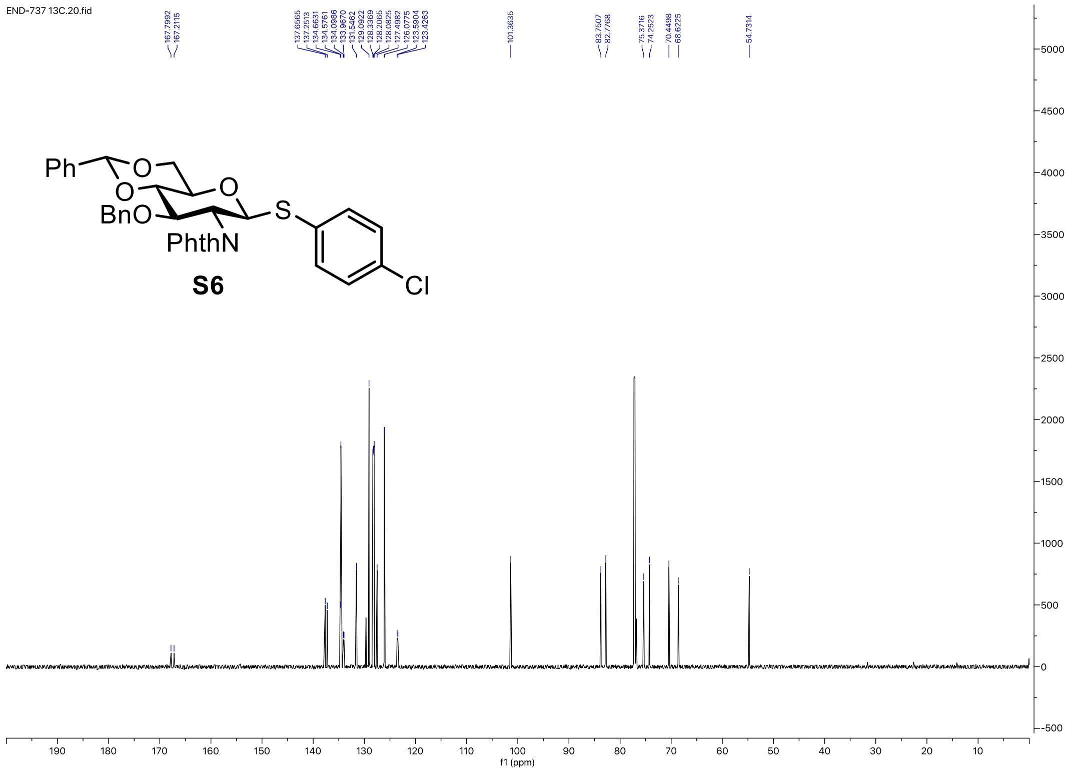

^{1}H NMR

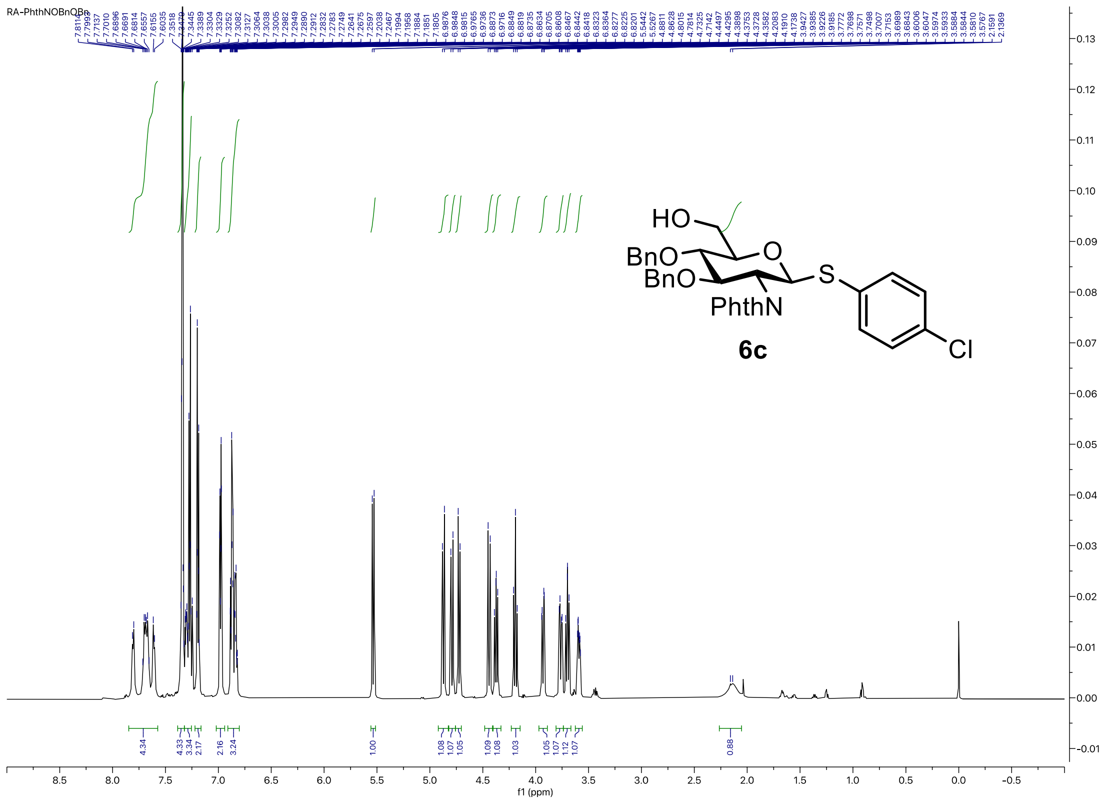

^{13}C NMR

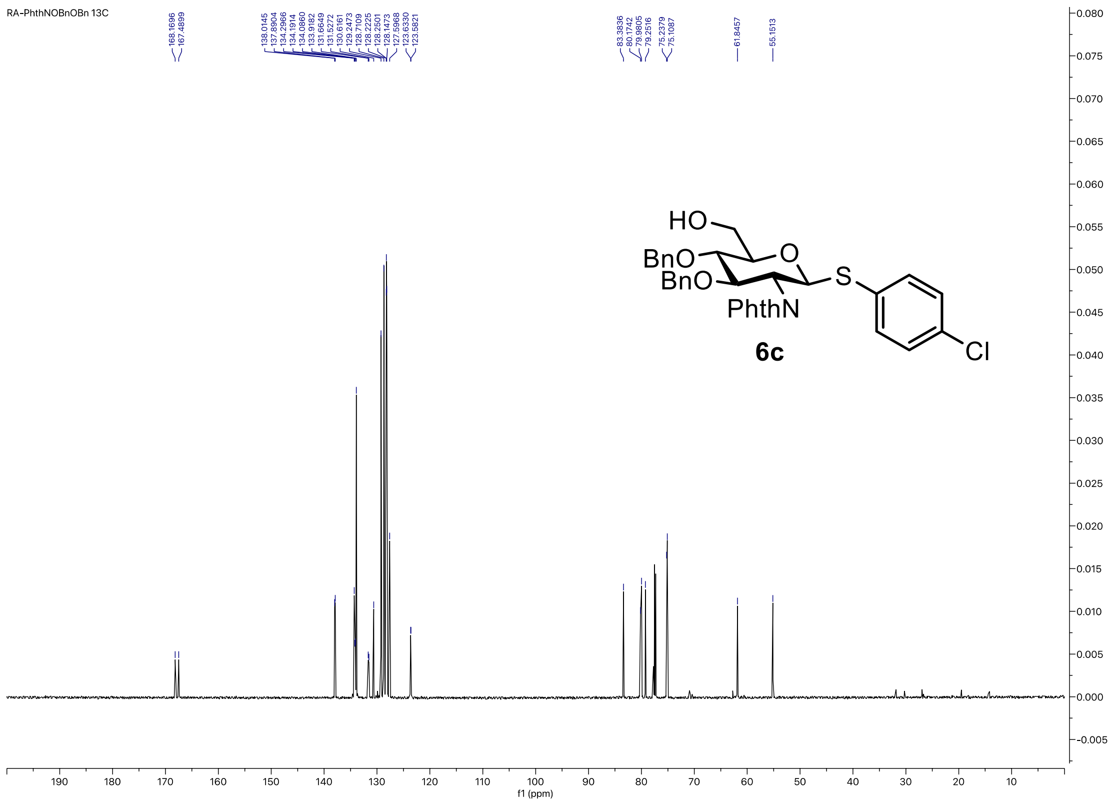

^{1}H NMR

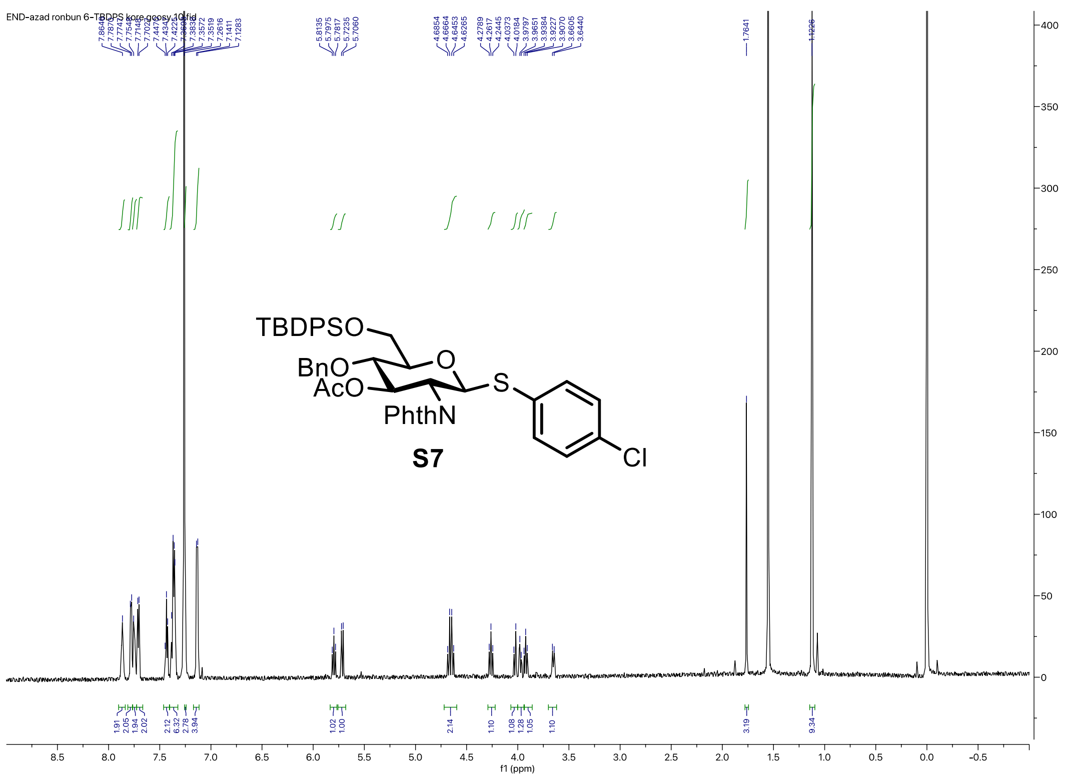

^{13}C NMR

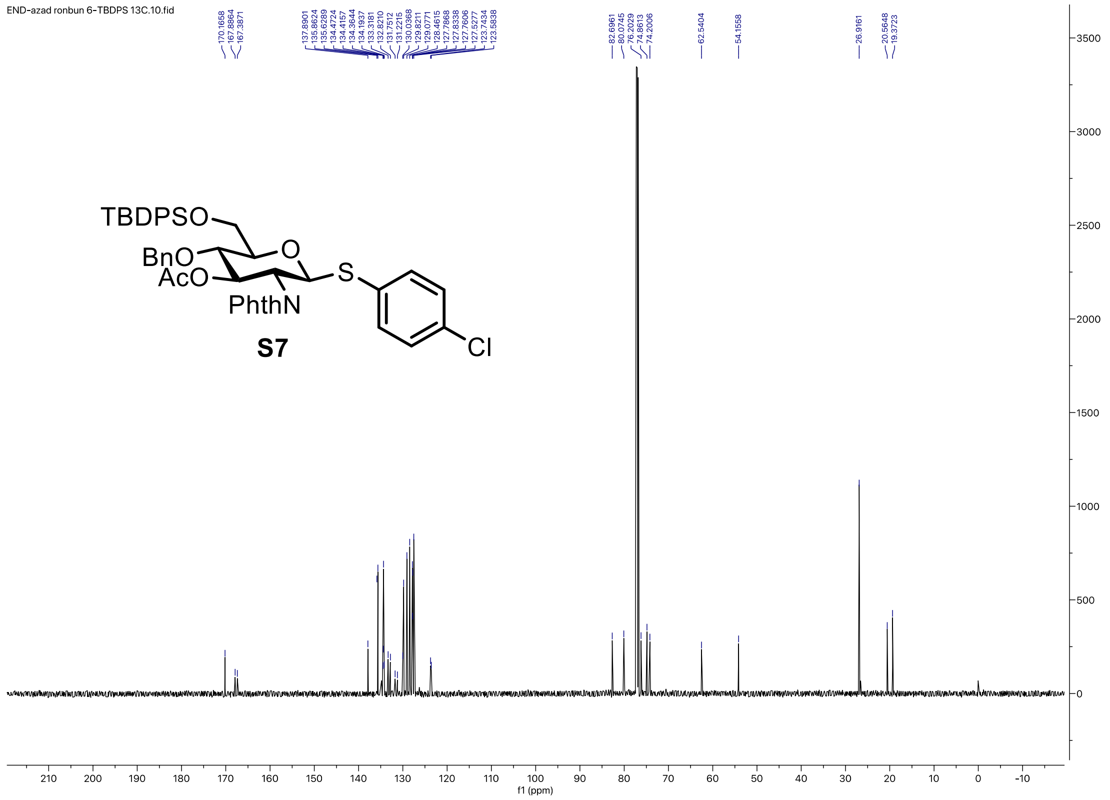

^{1}H NMR

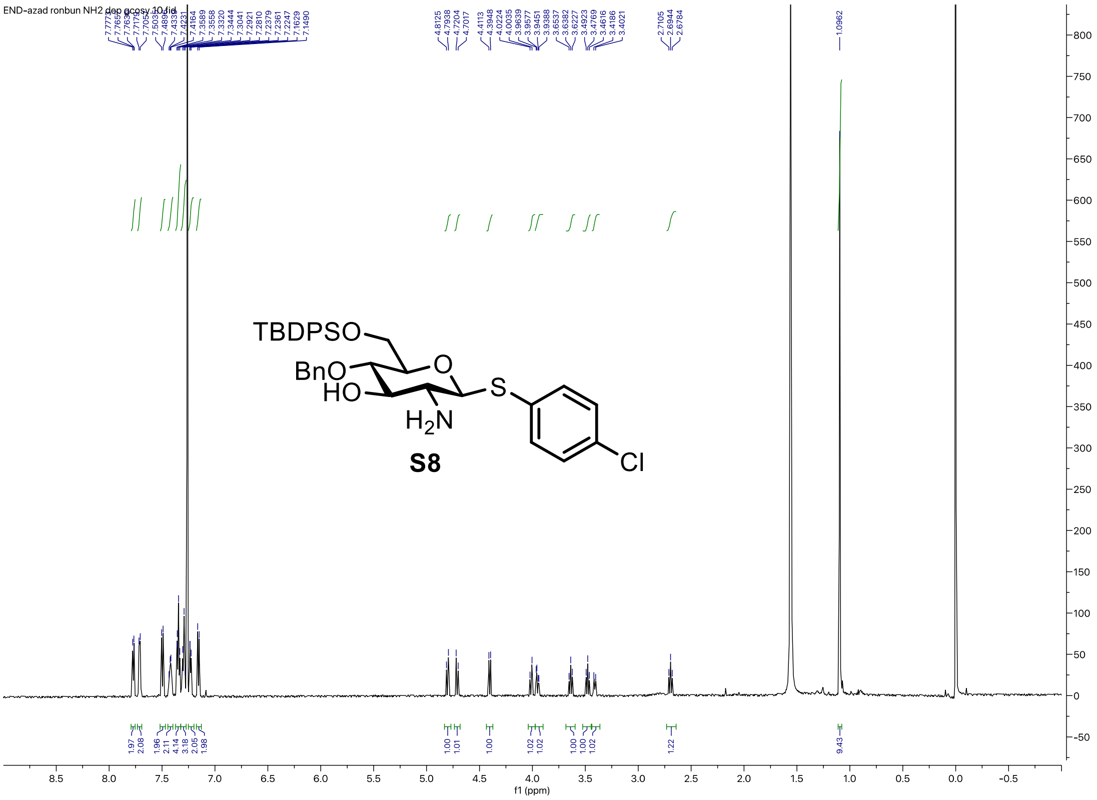

^{13}C NMR

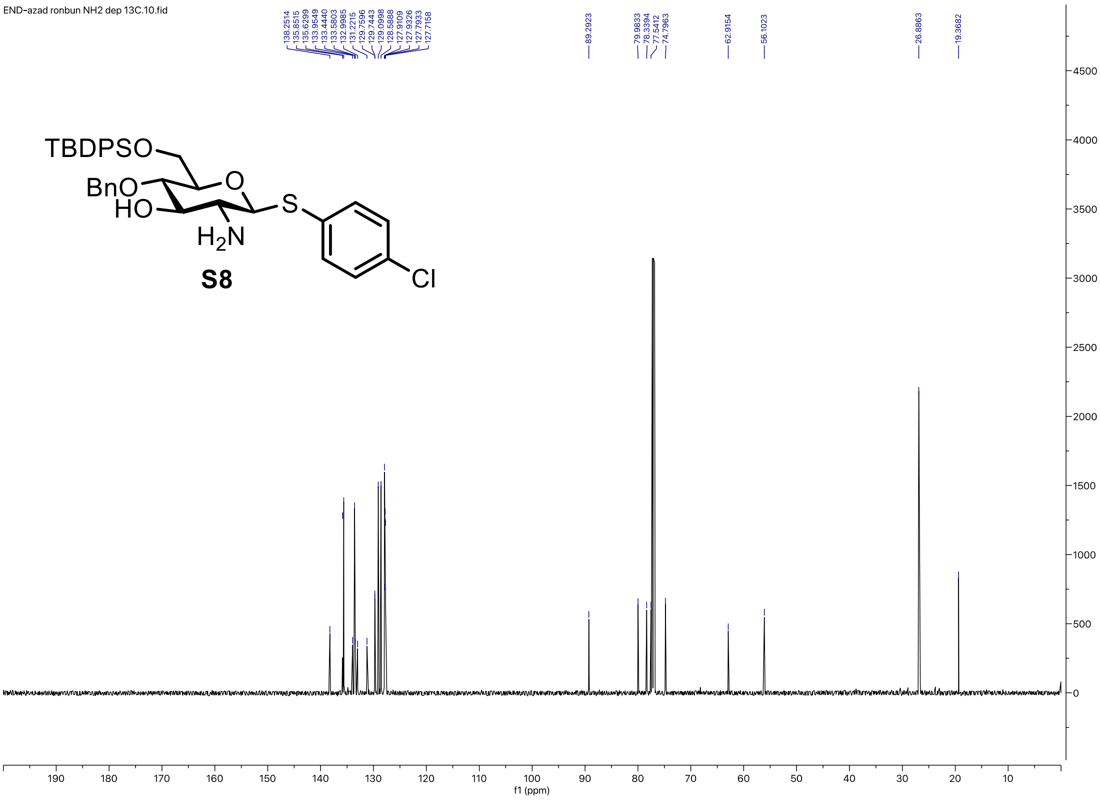

^{1}H NMR

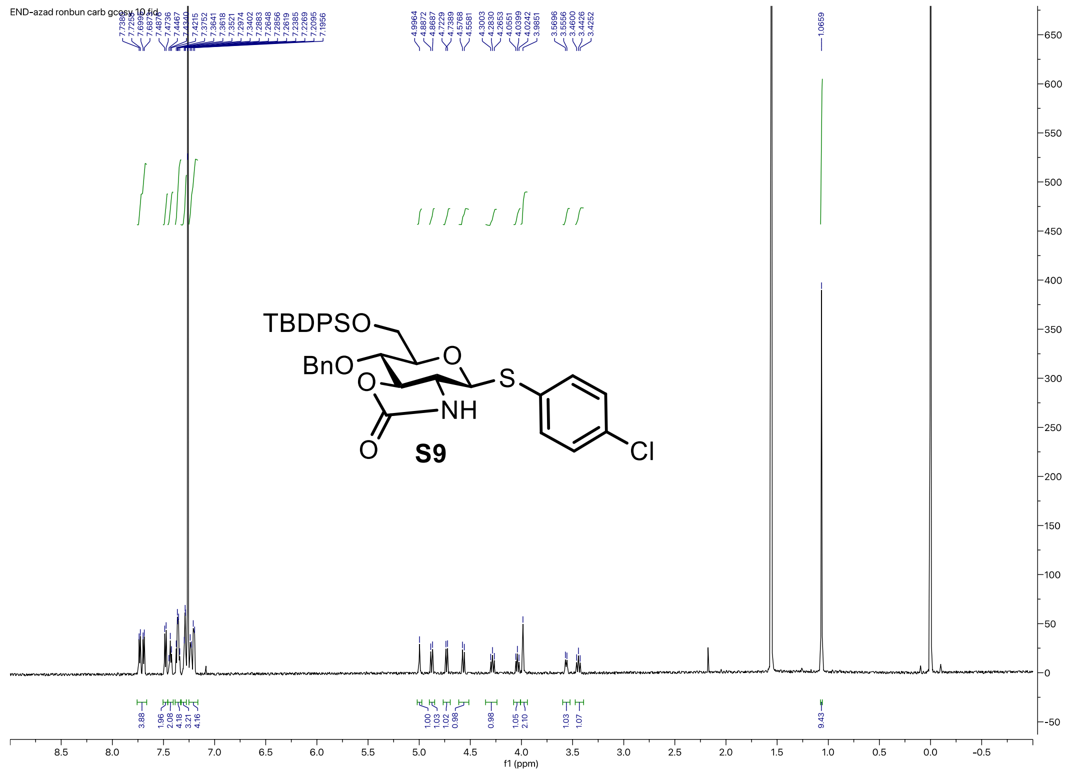

^{13}C NMR

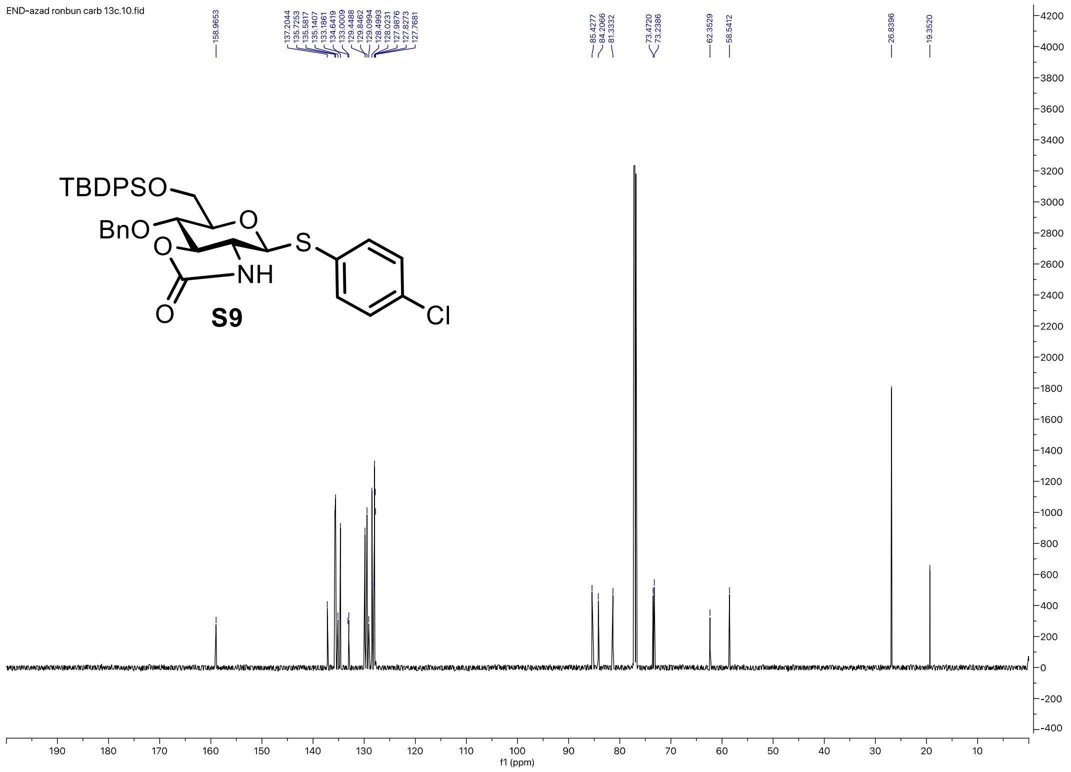

^{1}H NMR

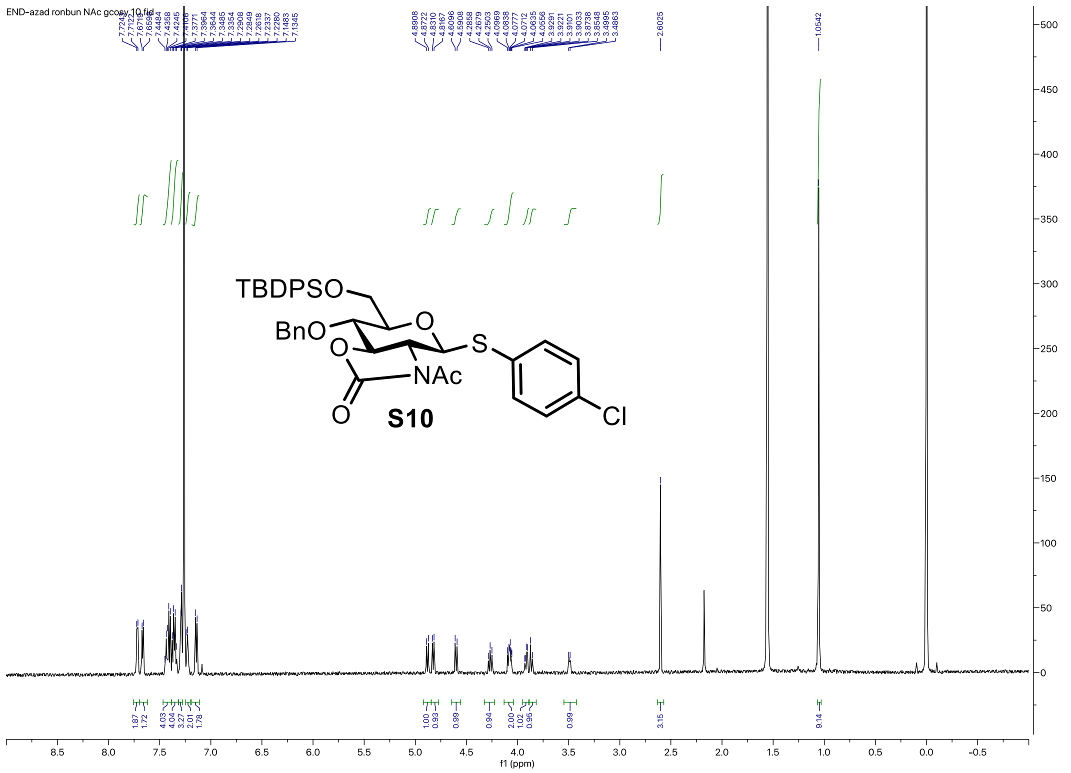

^{13}C NMR

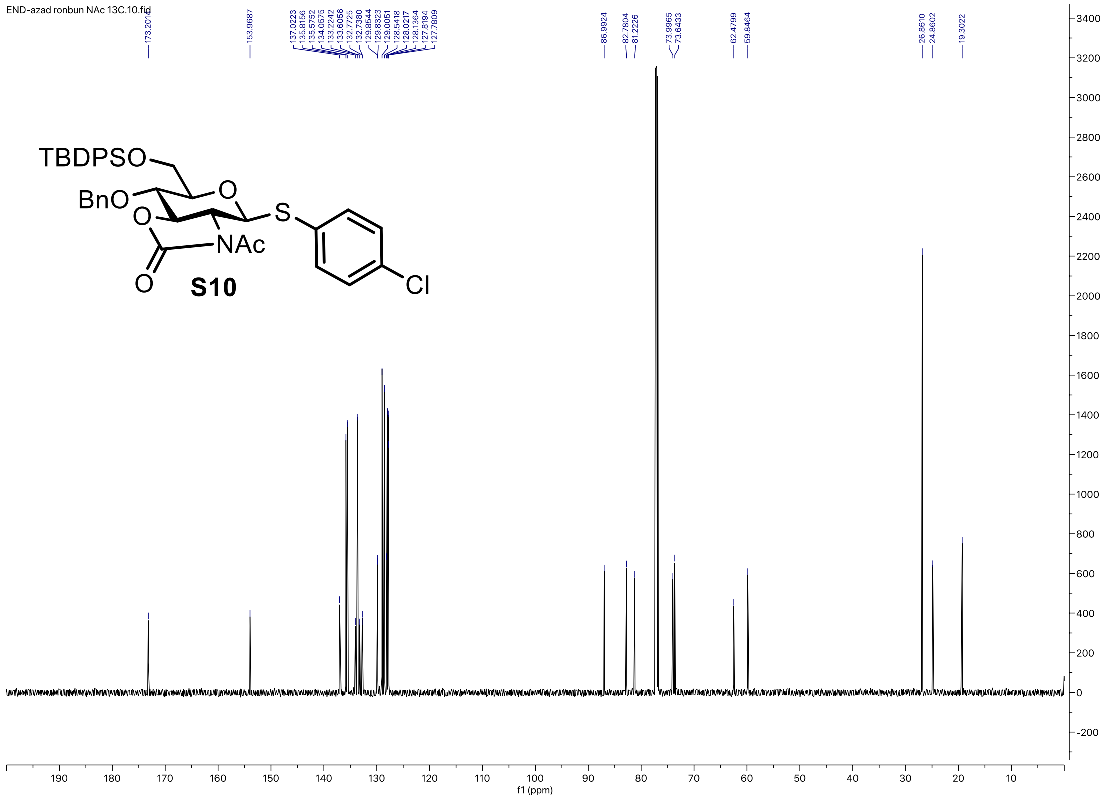

^{1}H NMR

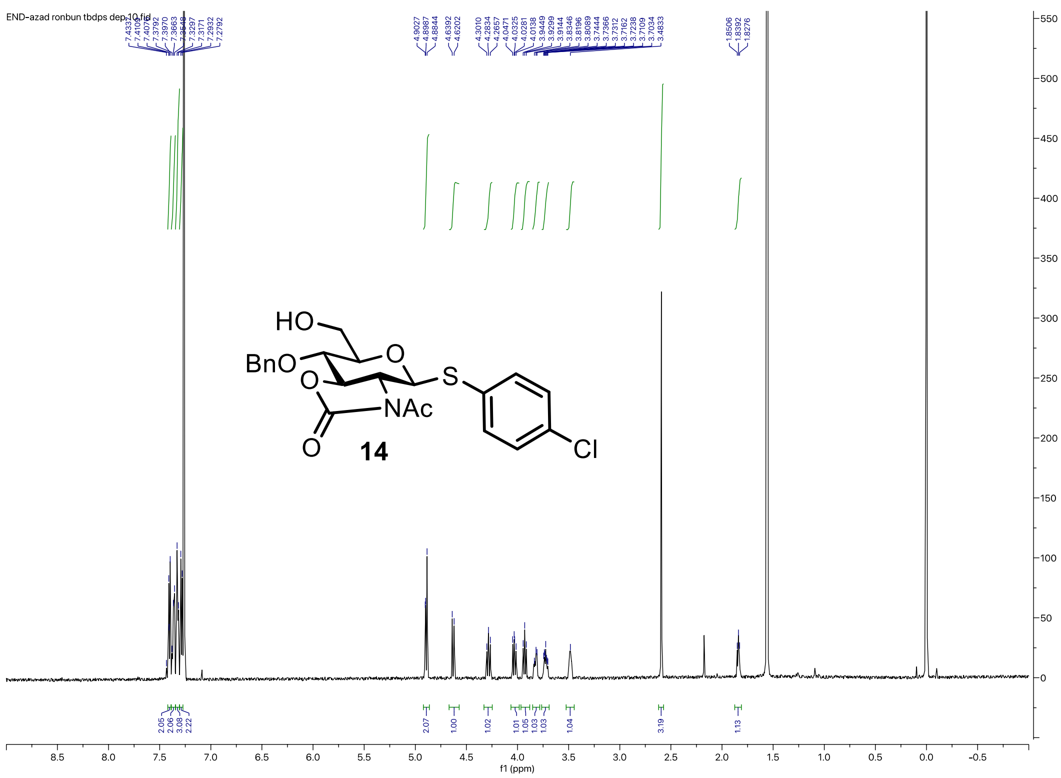

^{13}C NMR

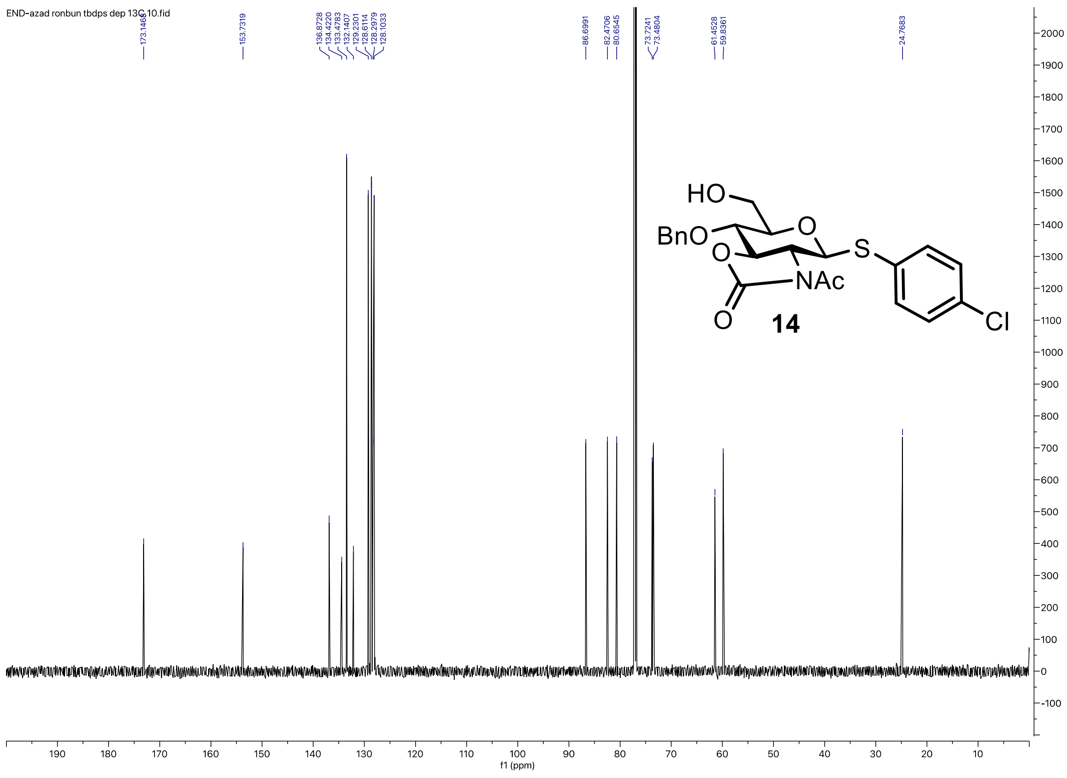

^{1}H NMR

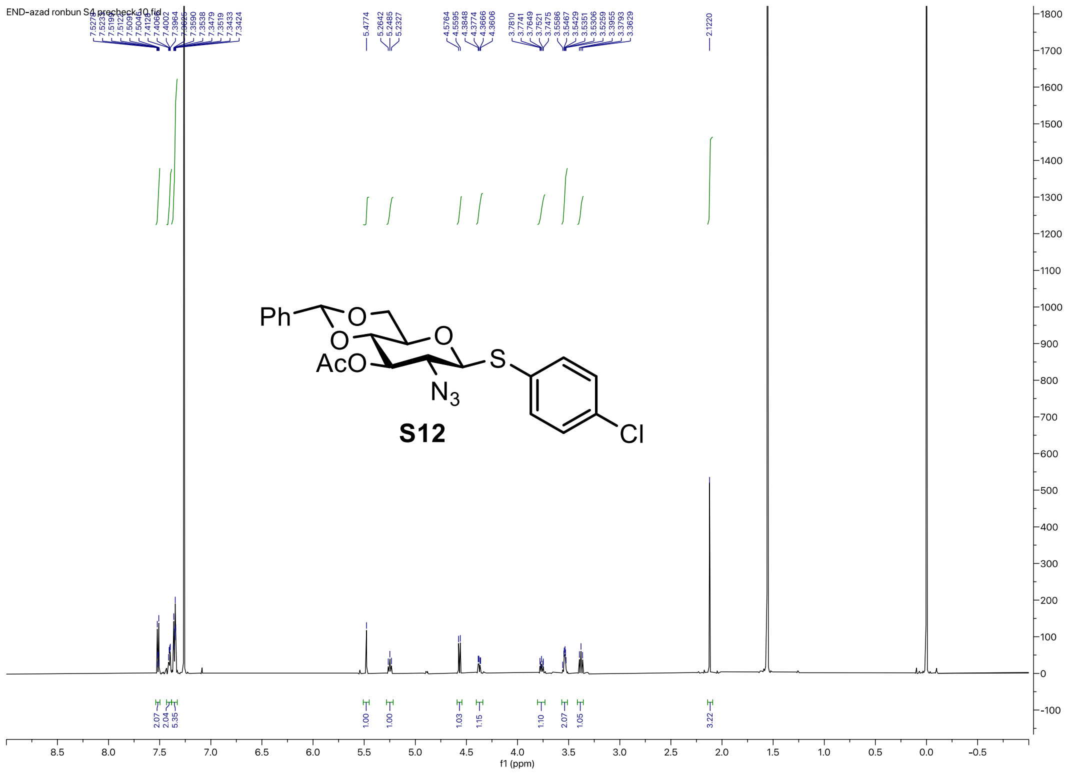

^{13}C NMR

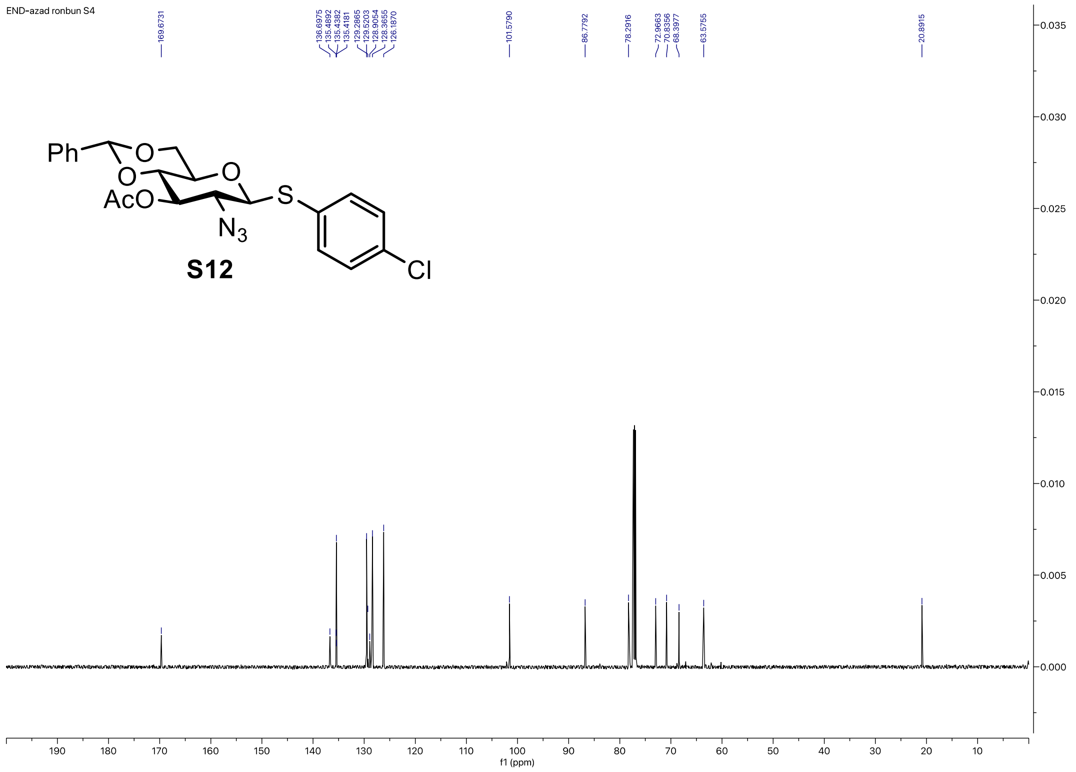

^{1}H NMR

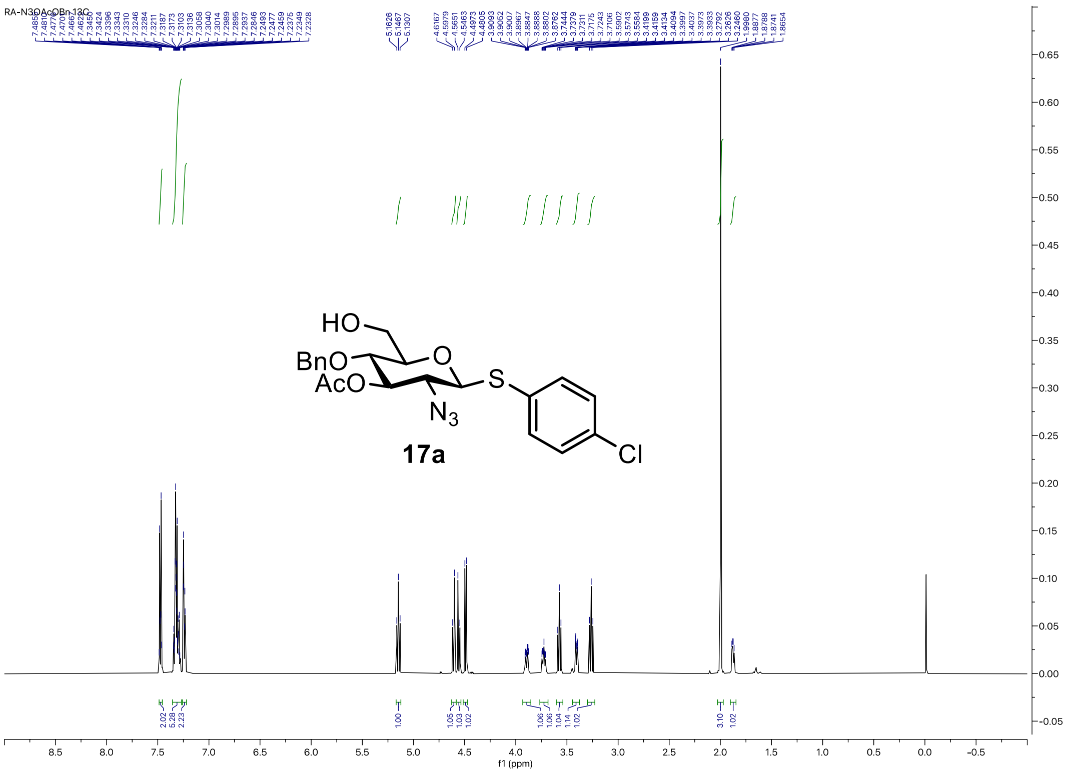

^{13}C NMR

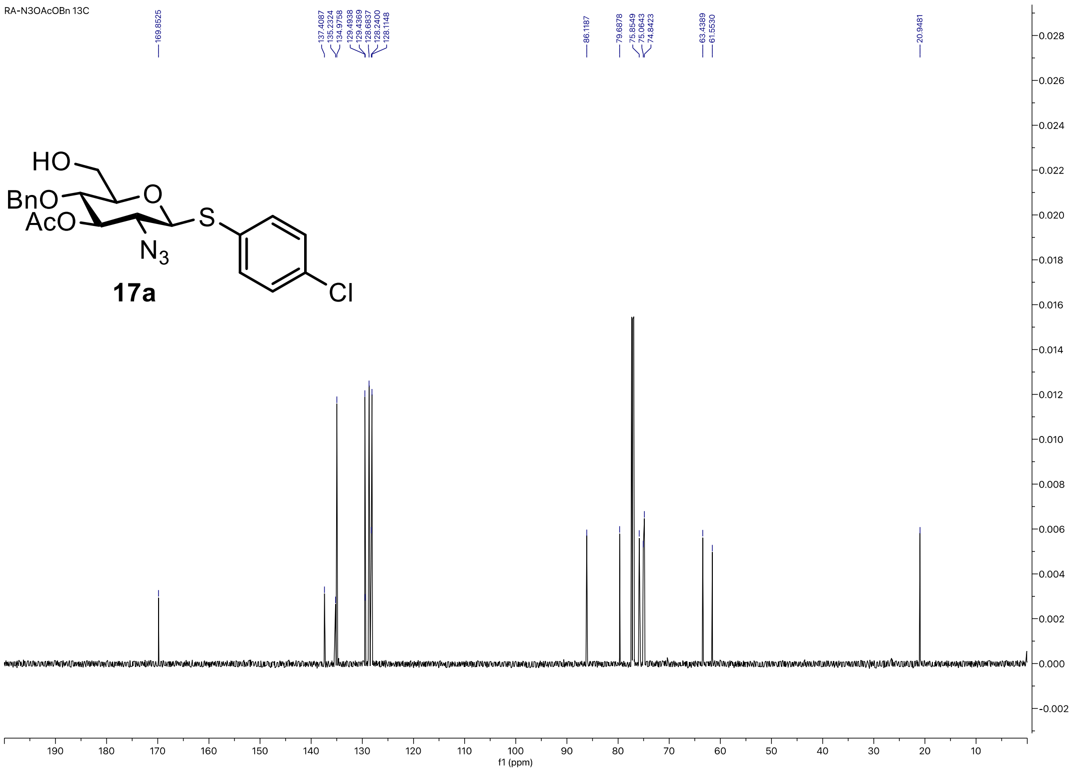

^{1}H NMR

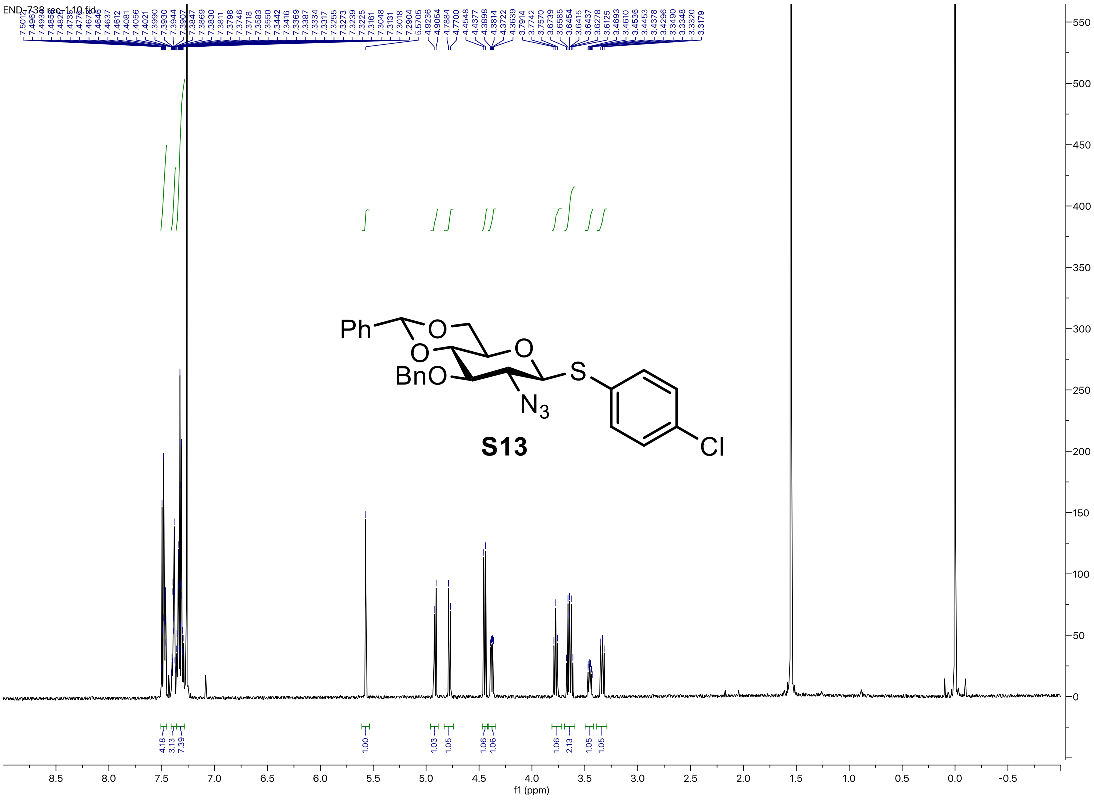

^{13}C NMR

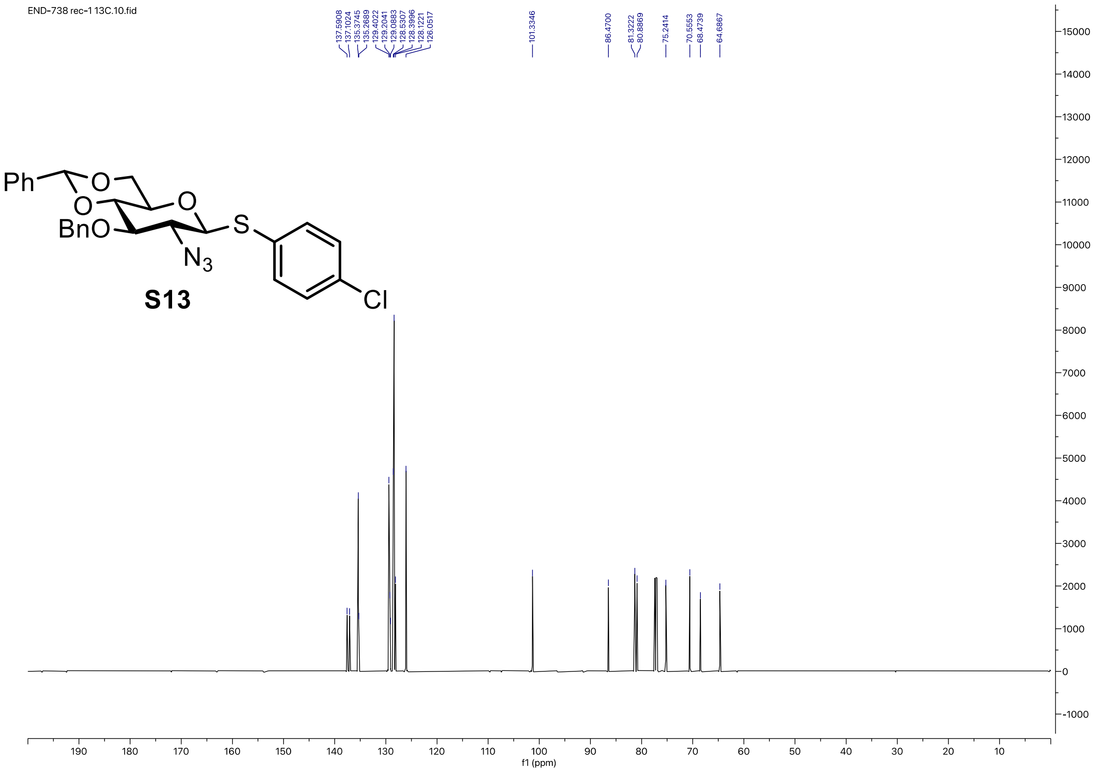

^{1}H NMR

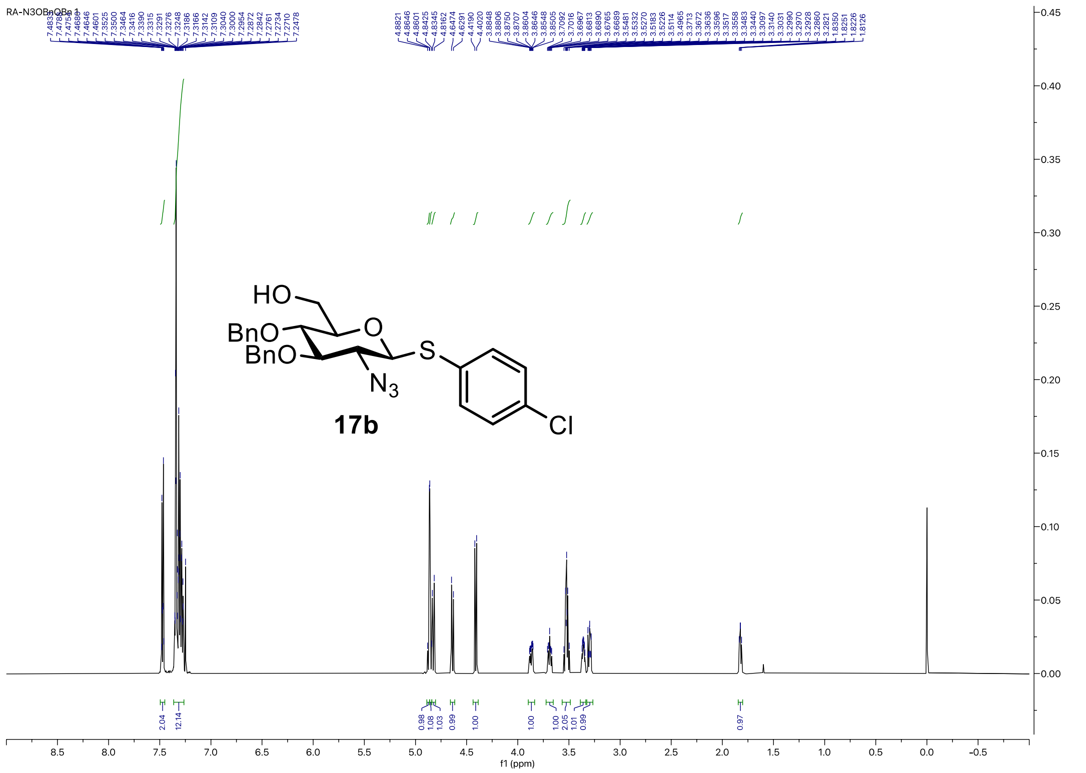

^{13}C NMR

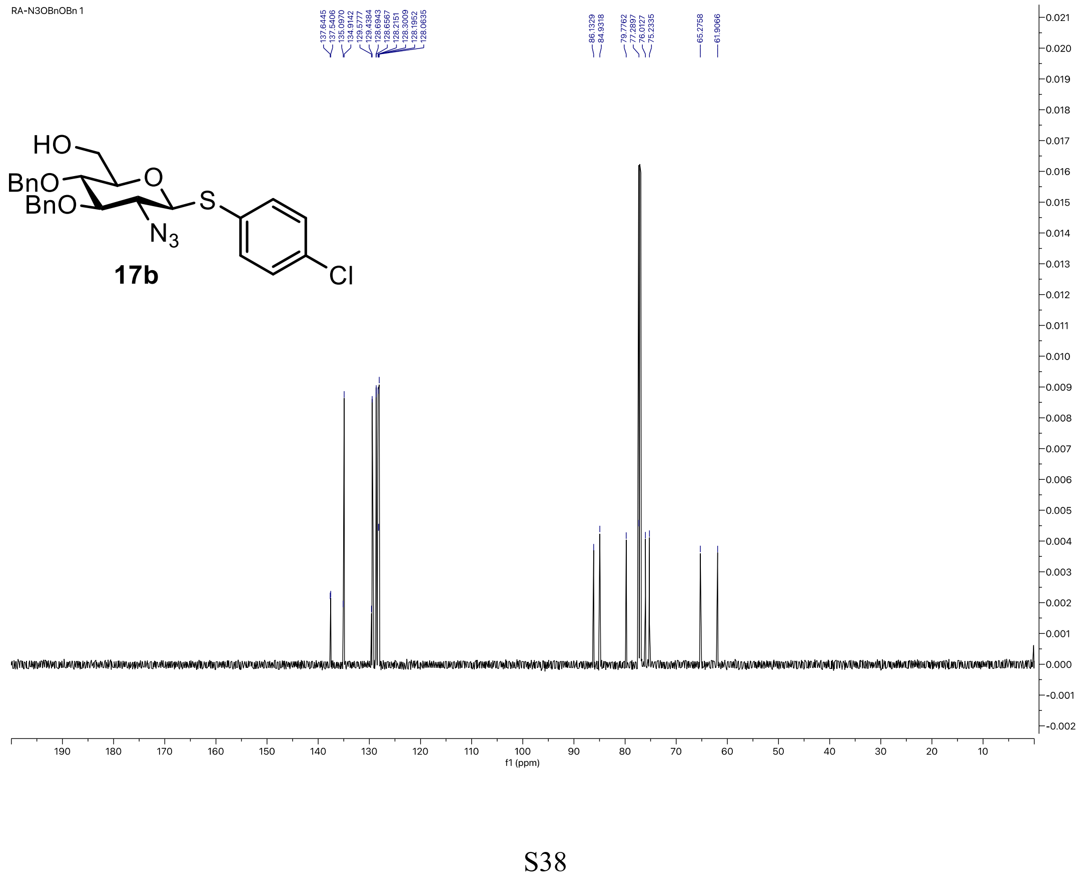

^{1}H NMR

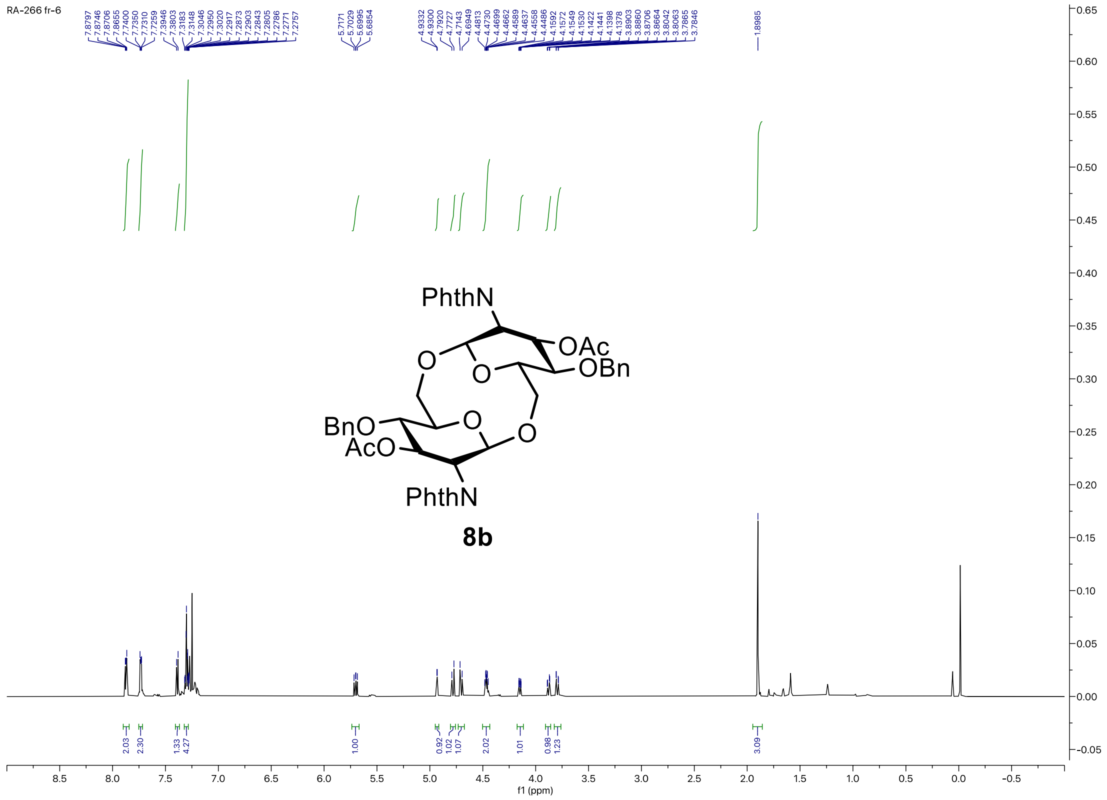

^{13}C NMR

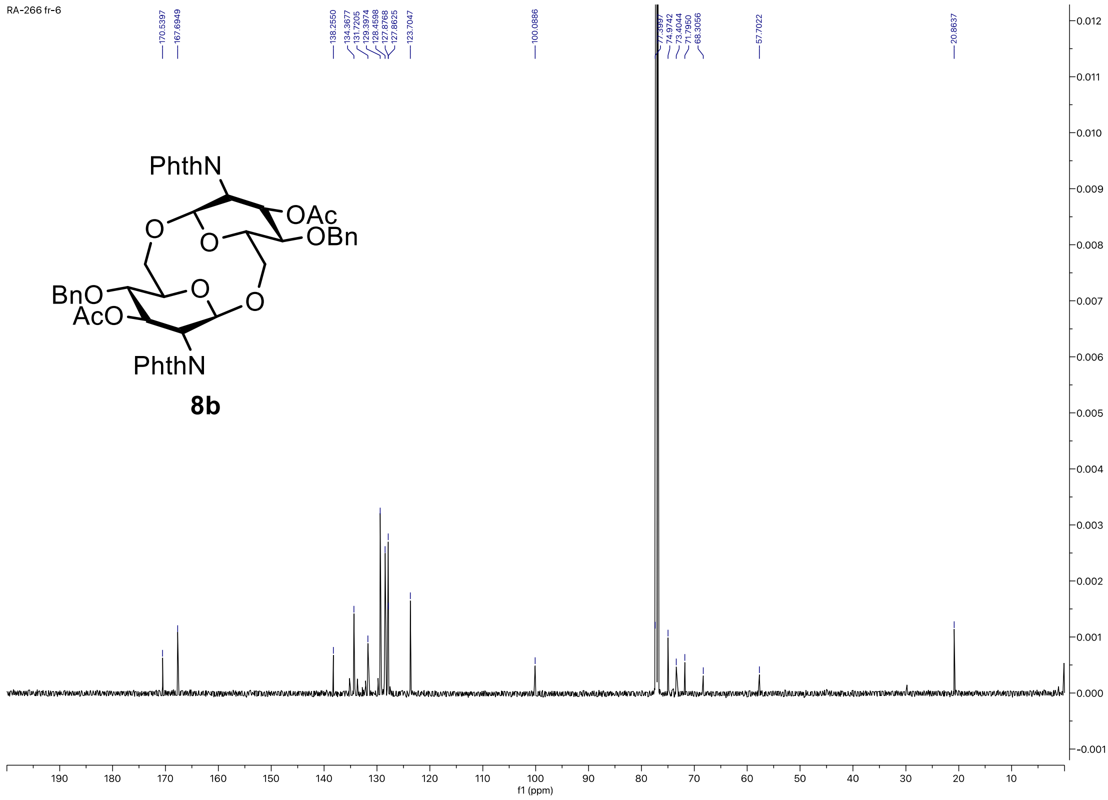

^{1}H, ^{1}H-COSY

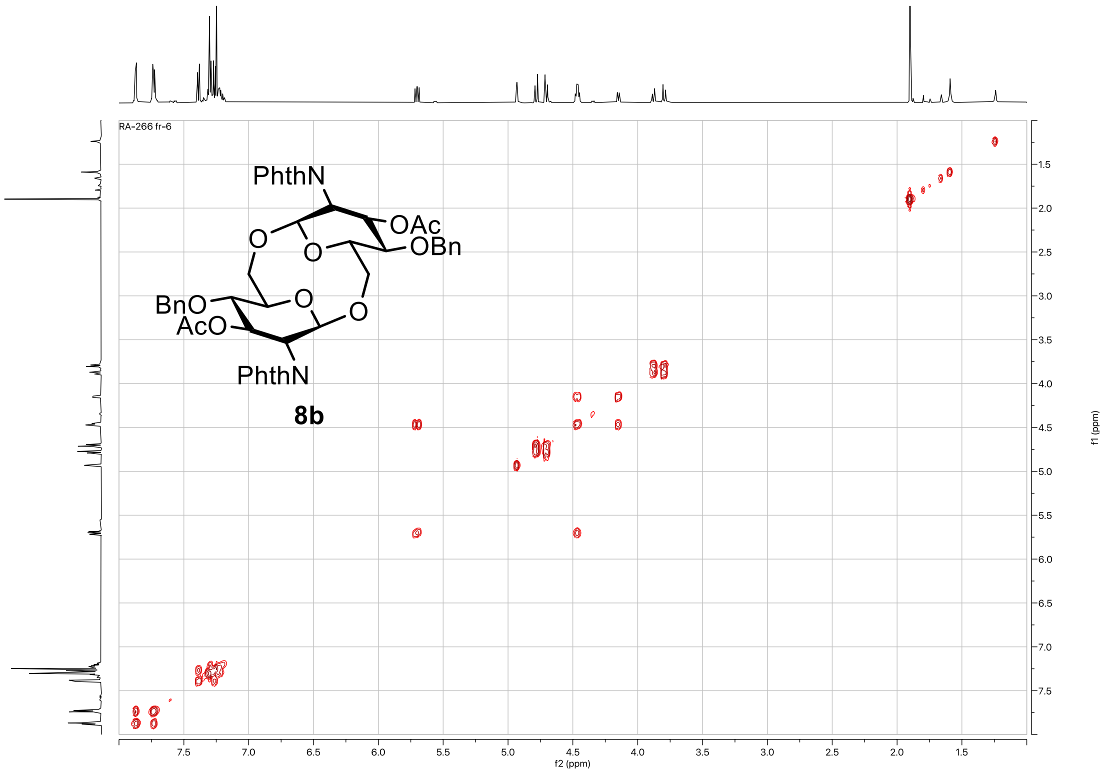

HMQC

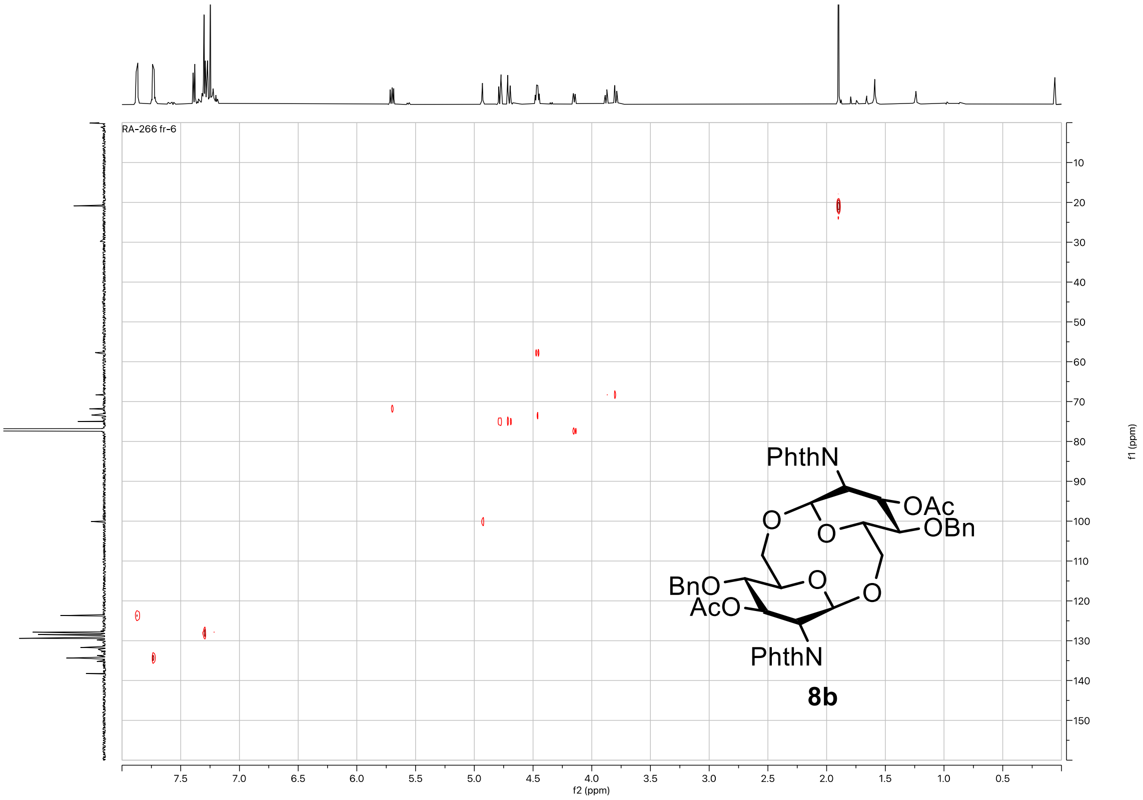

^{1}H NMR

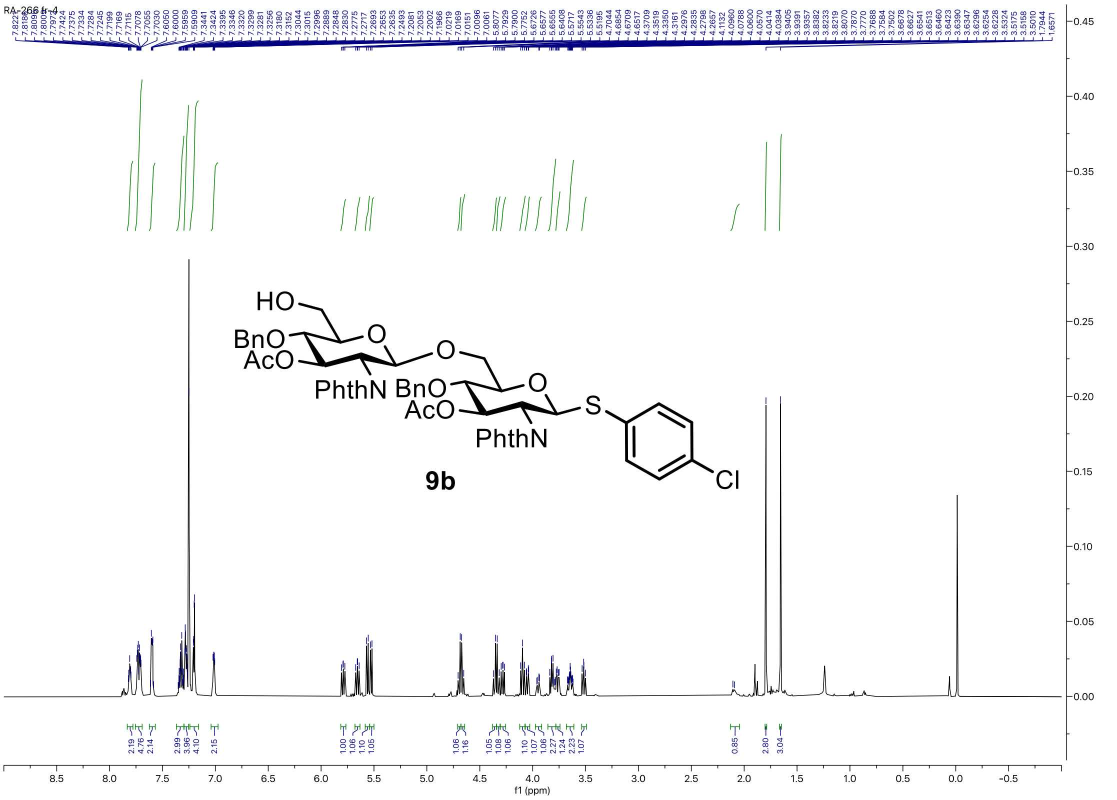

^{13}C NMR

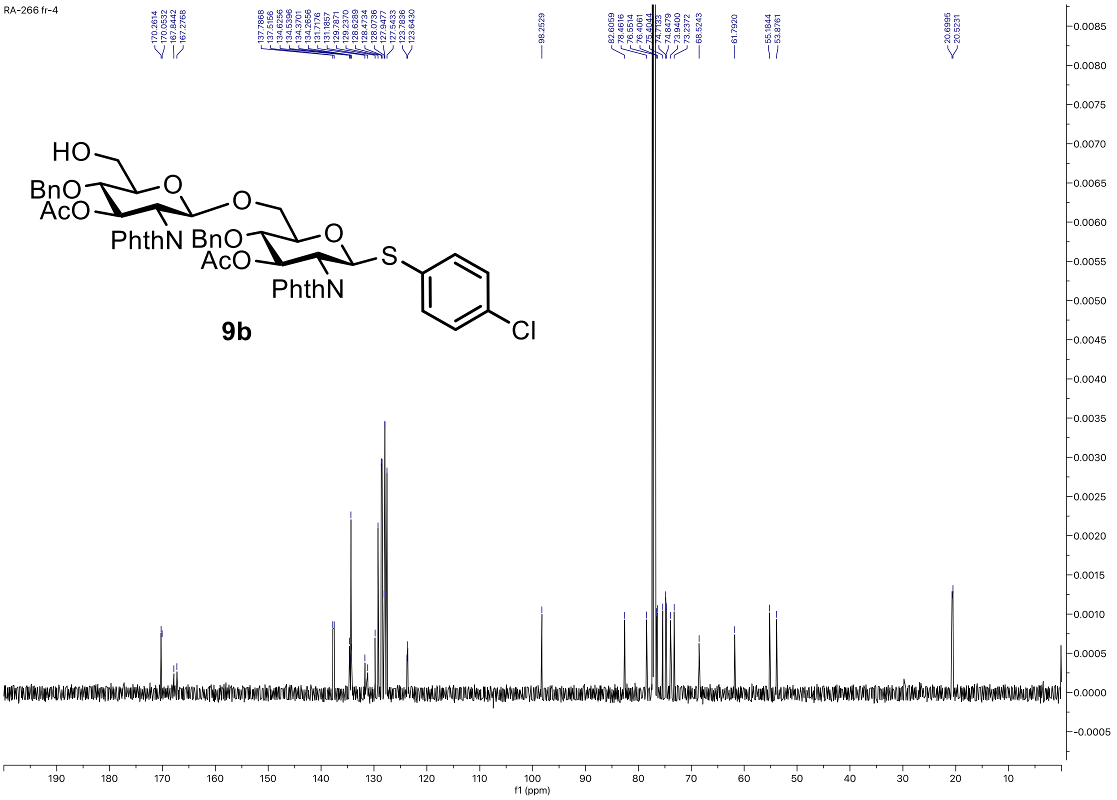

^{1}H, ^{1}H-COSY

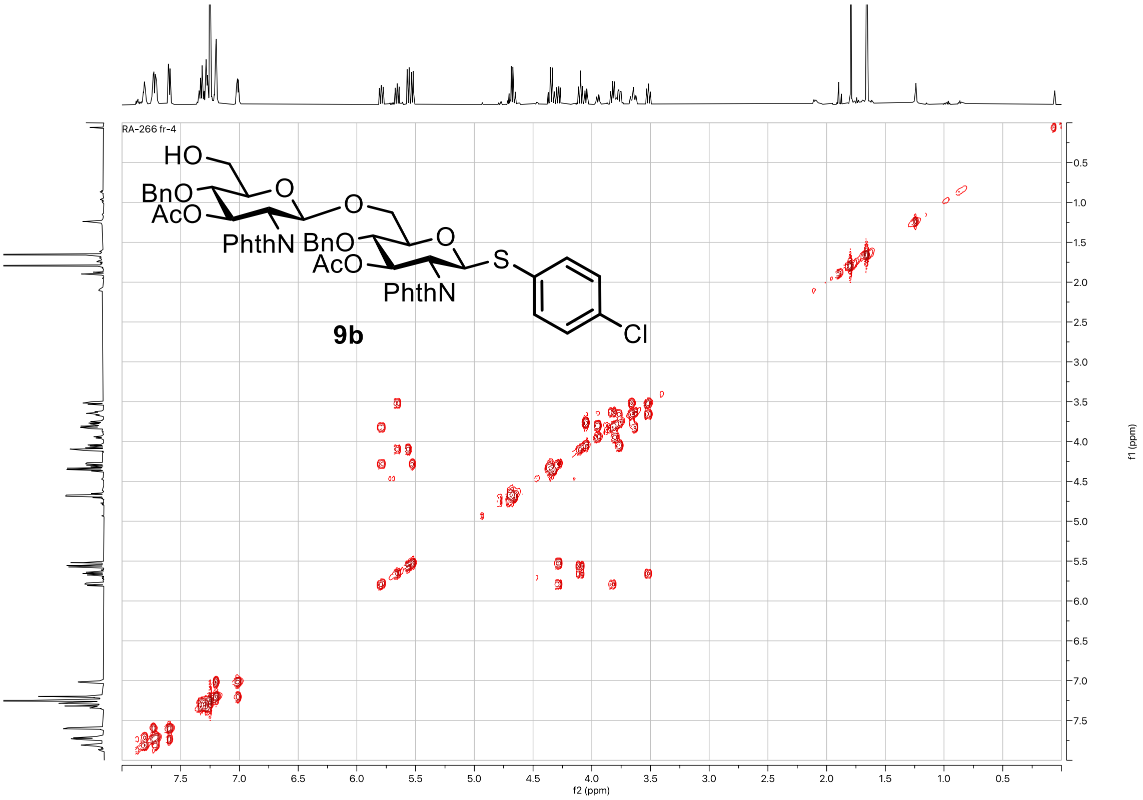

HMQC

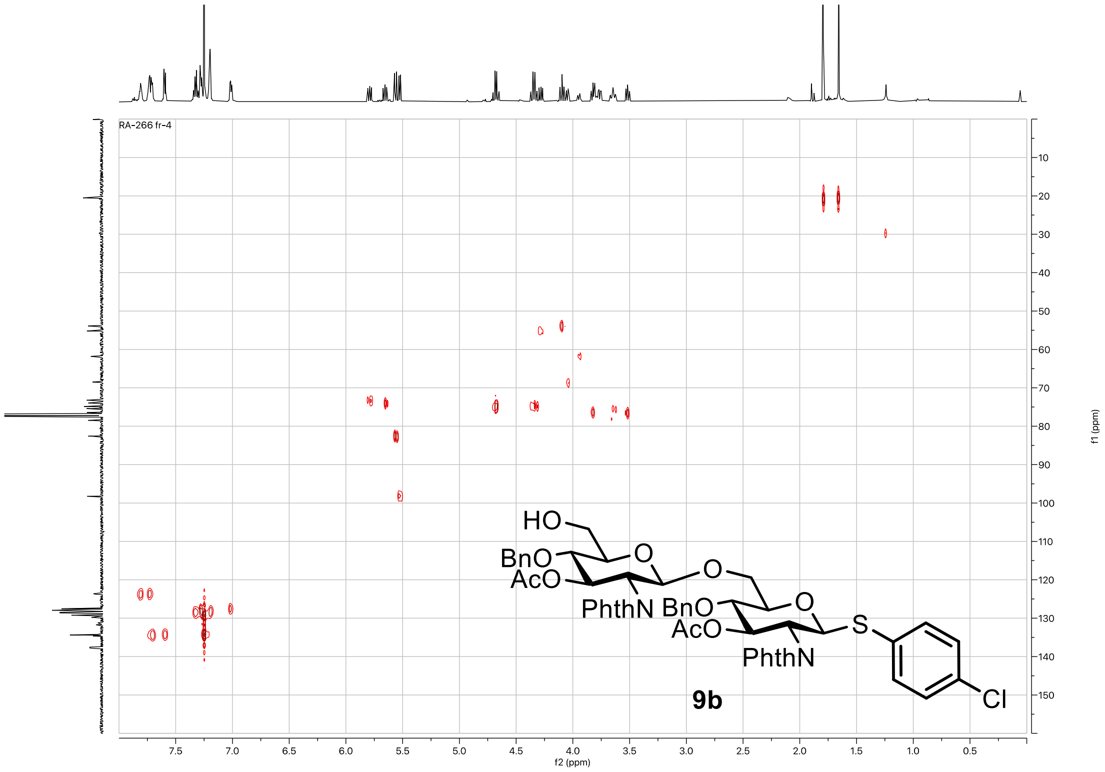

^{1}H NMR

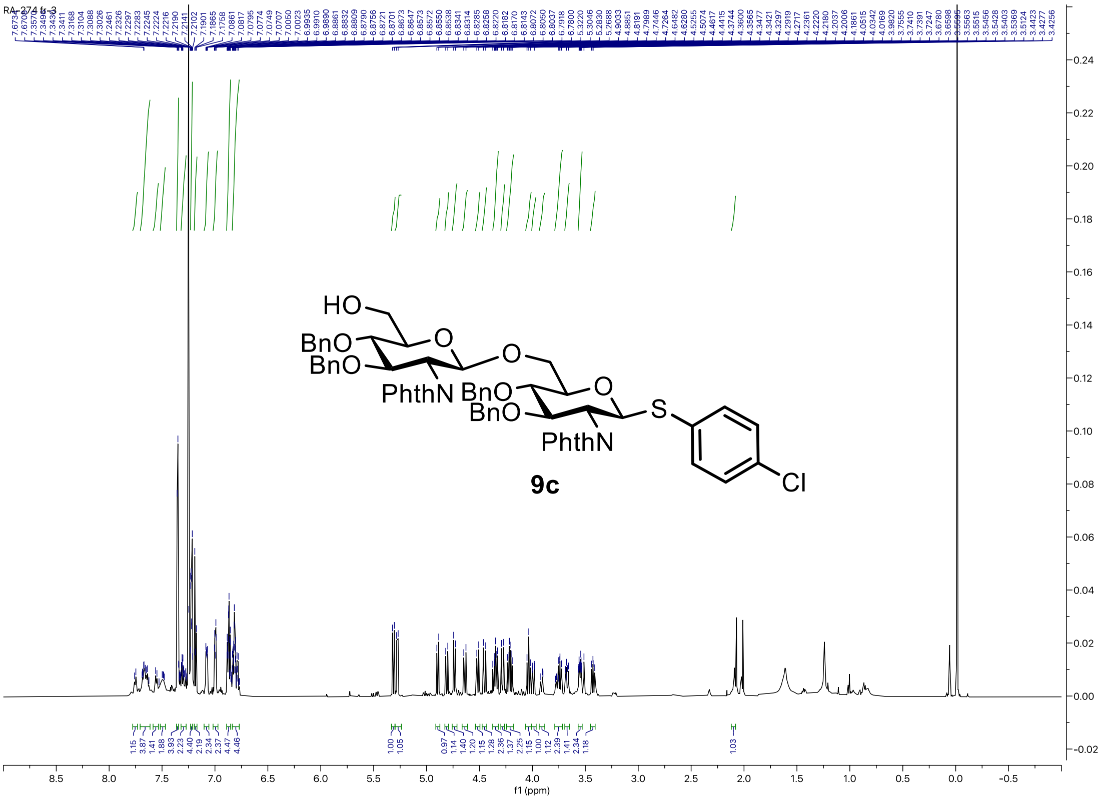

^{13}C NMR

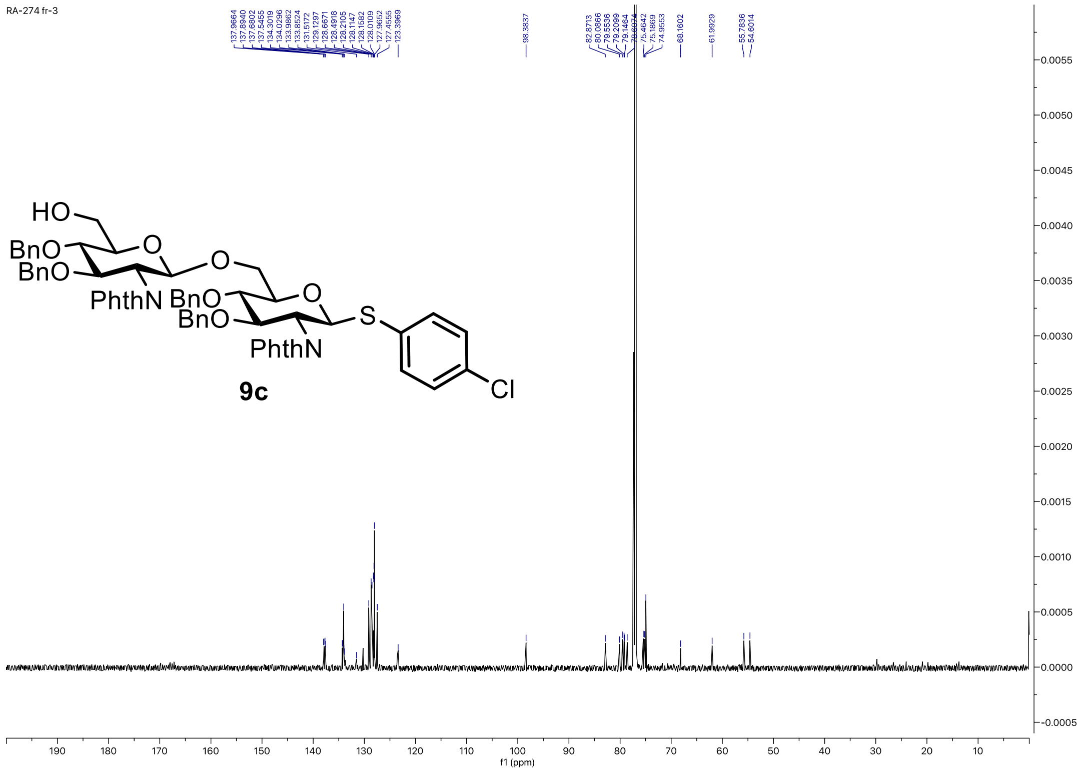

^{1}H, ^{1}H-COSY

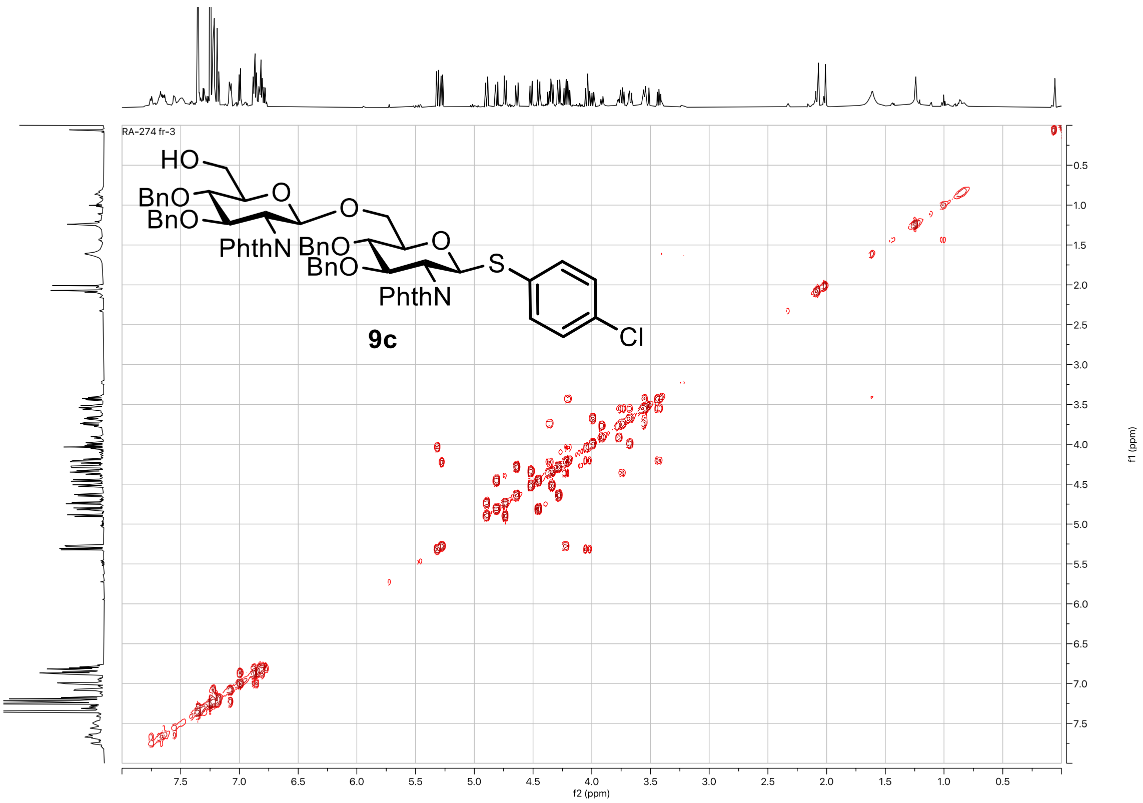

HMQC

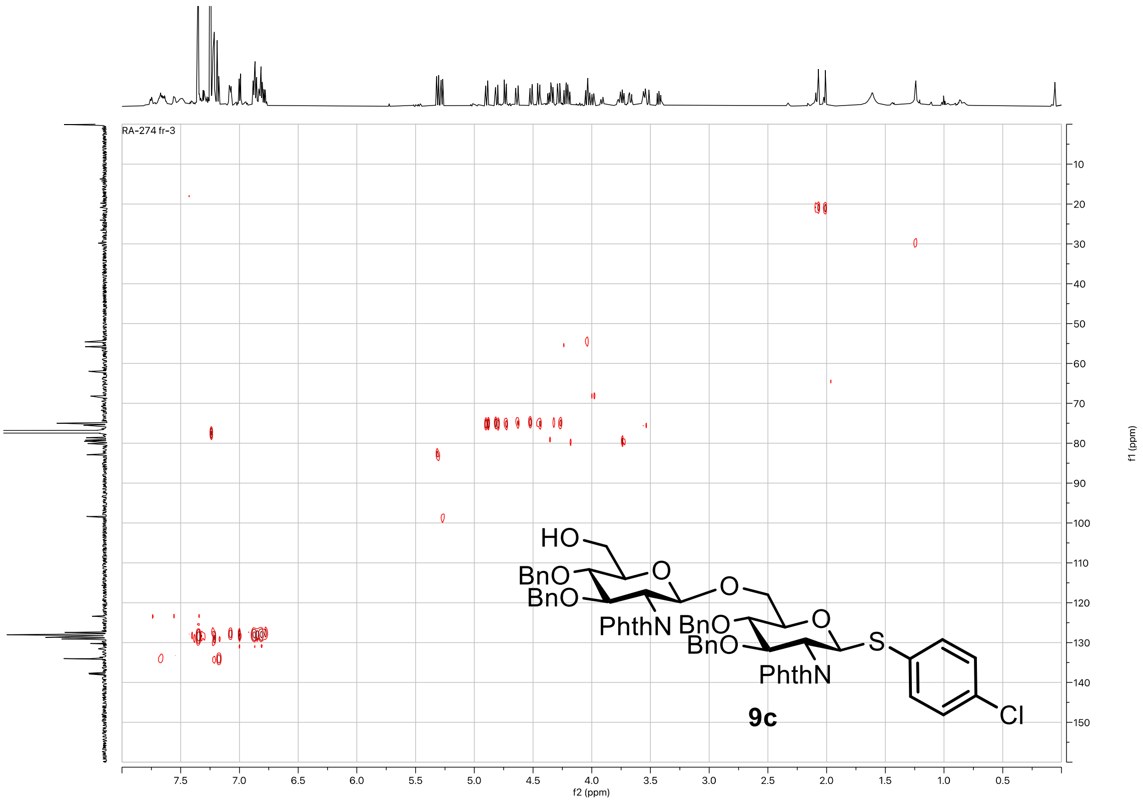

^{1}H NMR

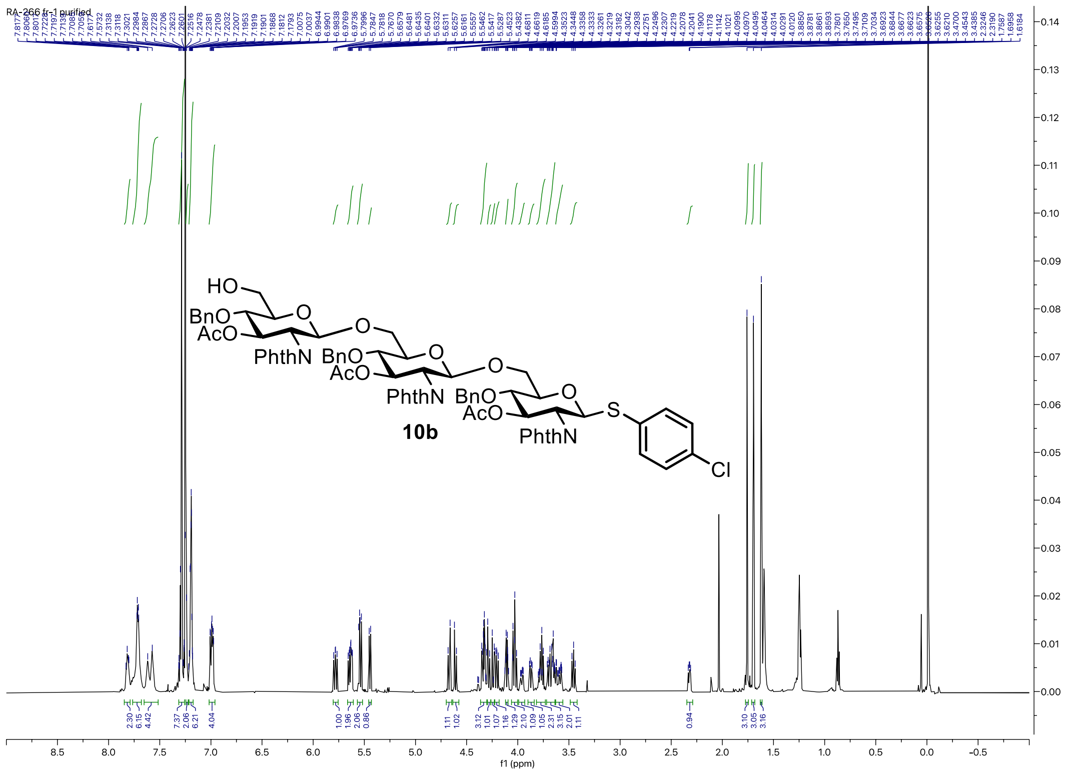

^{13}C NMR

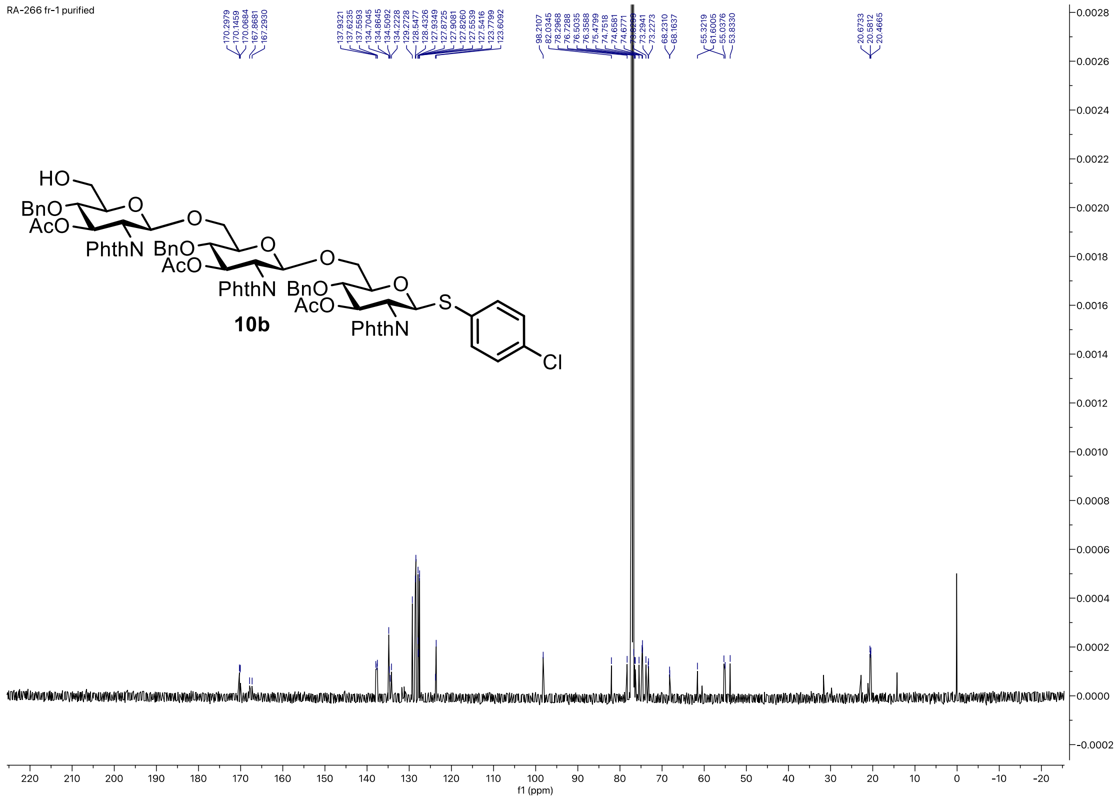

$^{1}H, ^{1}H$-COSY

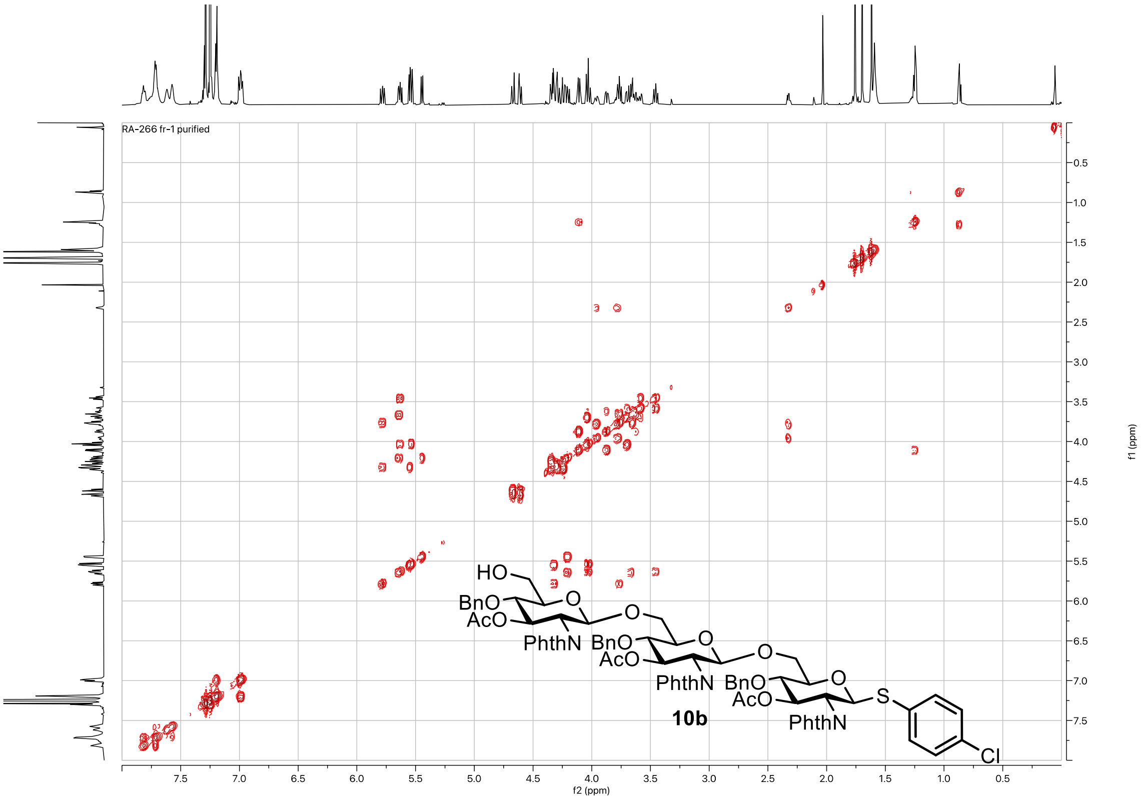

HMQC

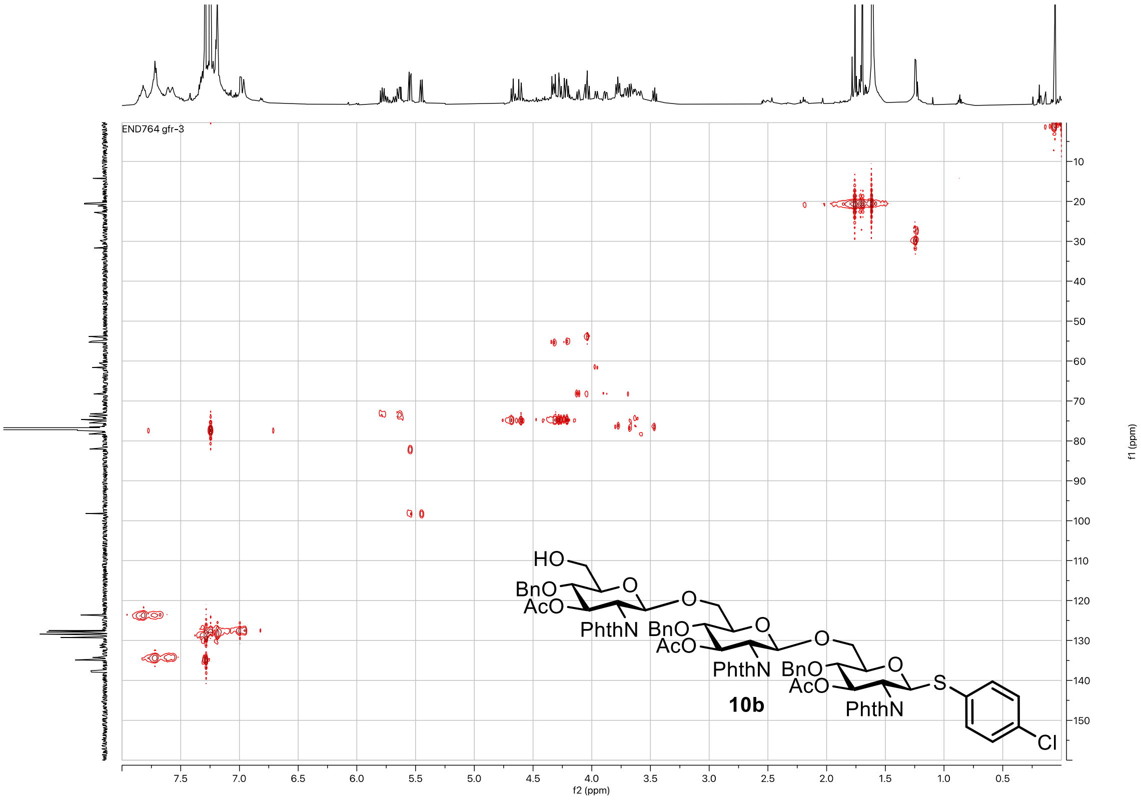

^{1}H NMR

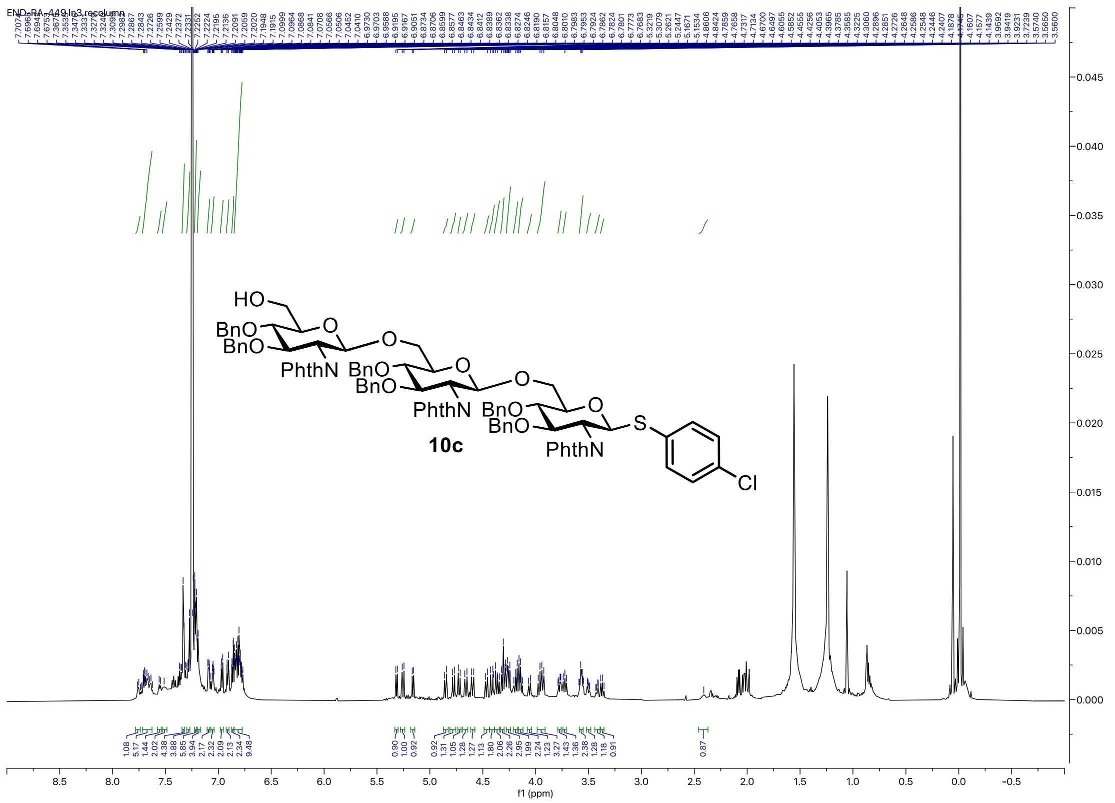

^{13}C NMR

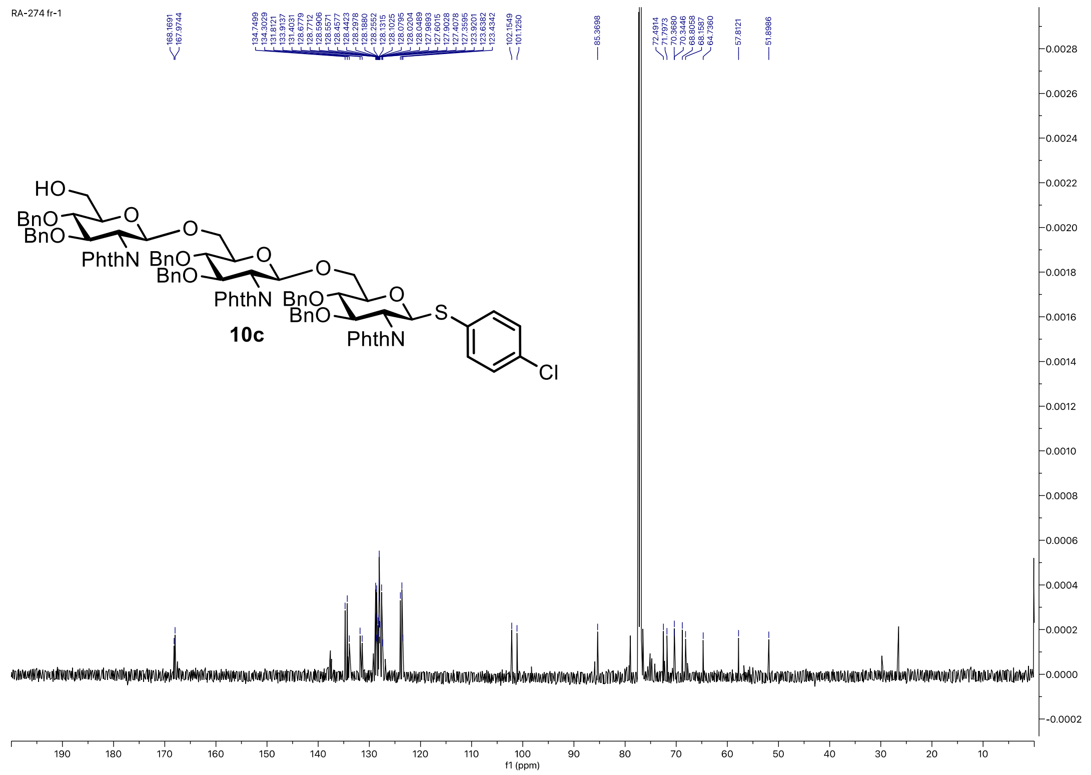

¹H, ¹H-COSY

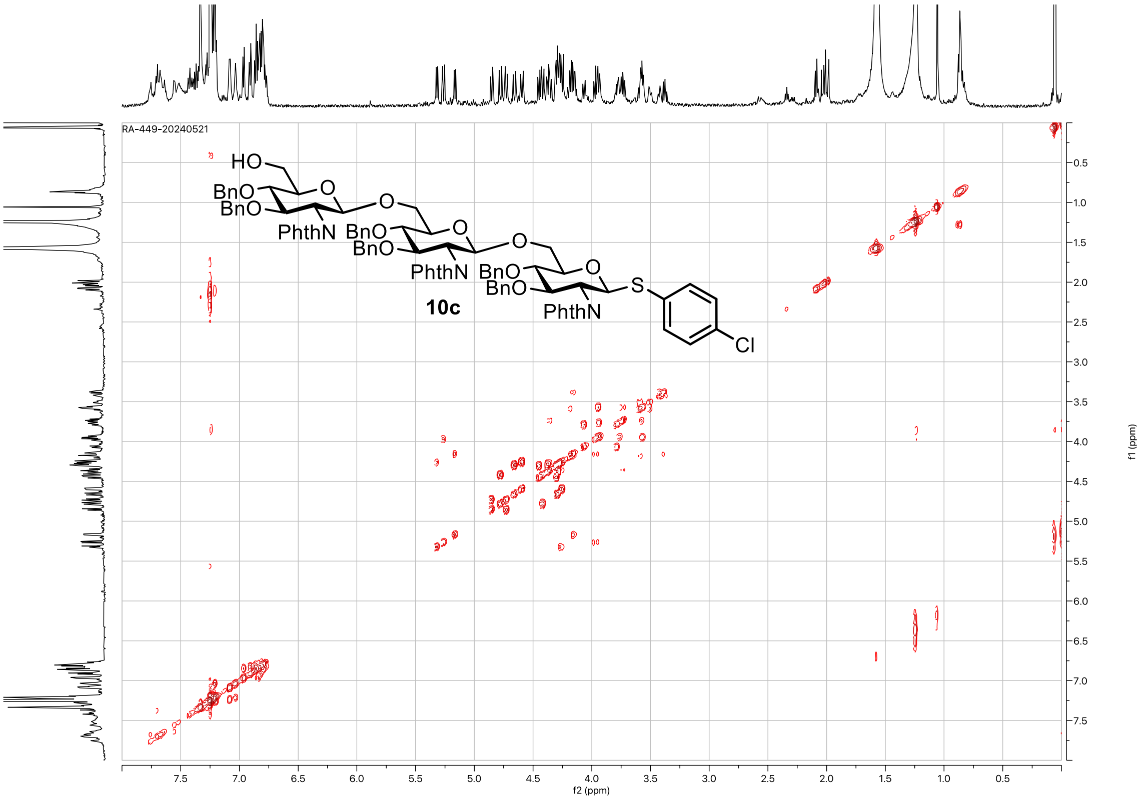

HMQC

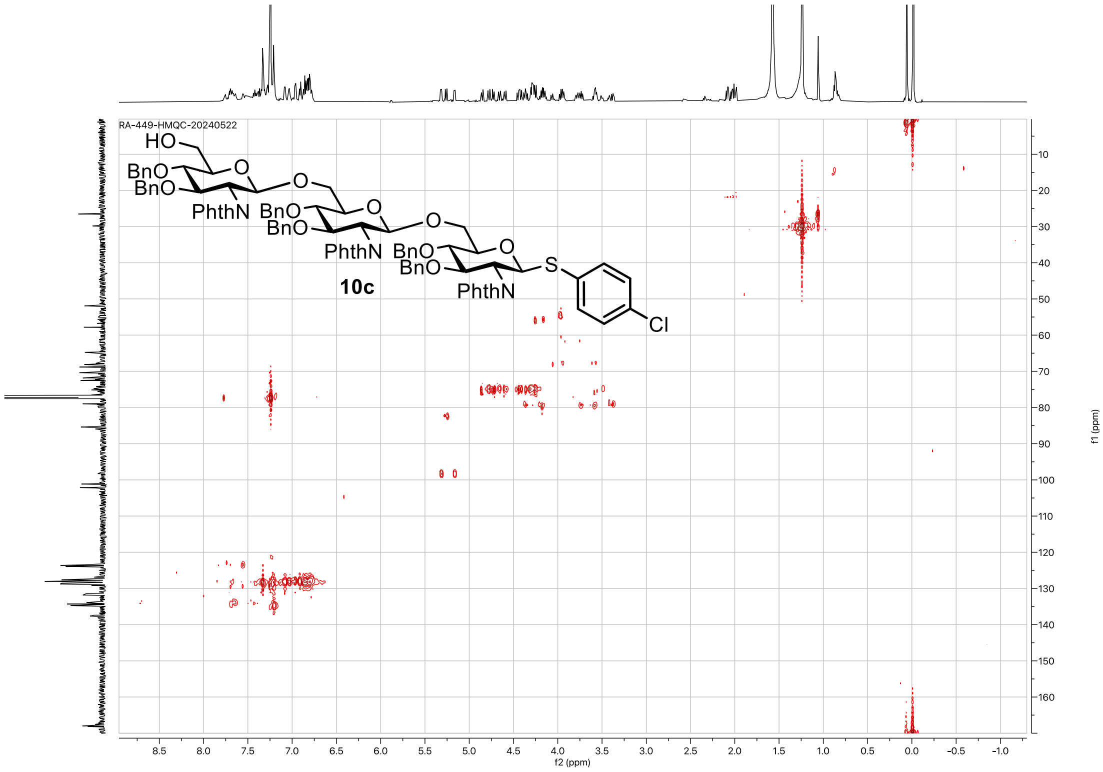

^{1}H NMR

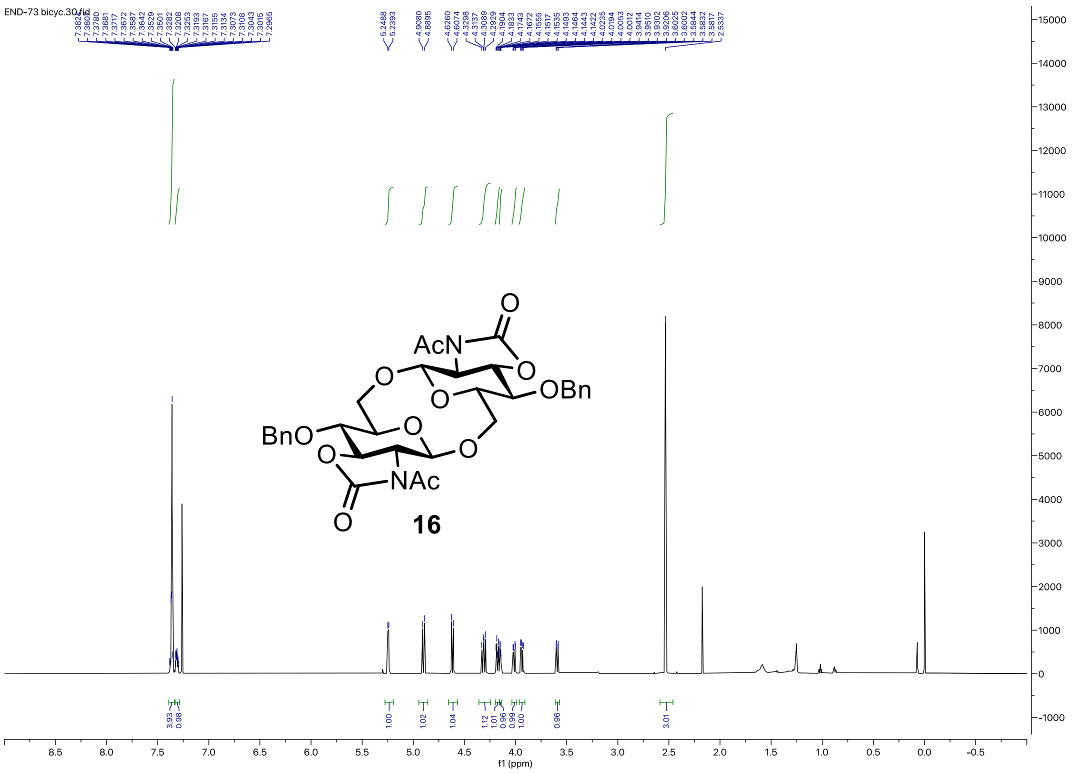

^{13}C NMR

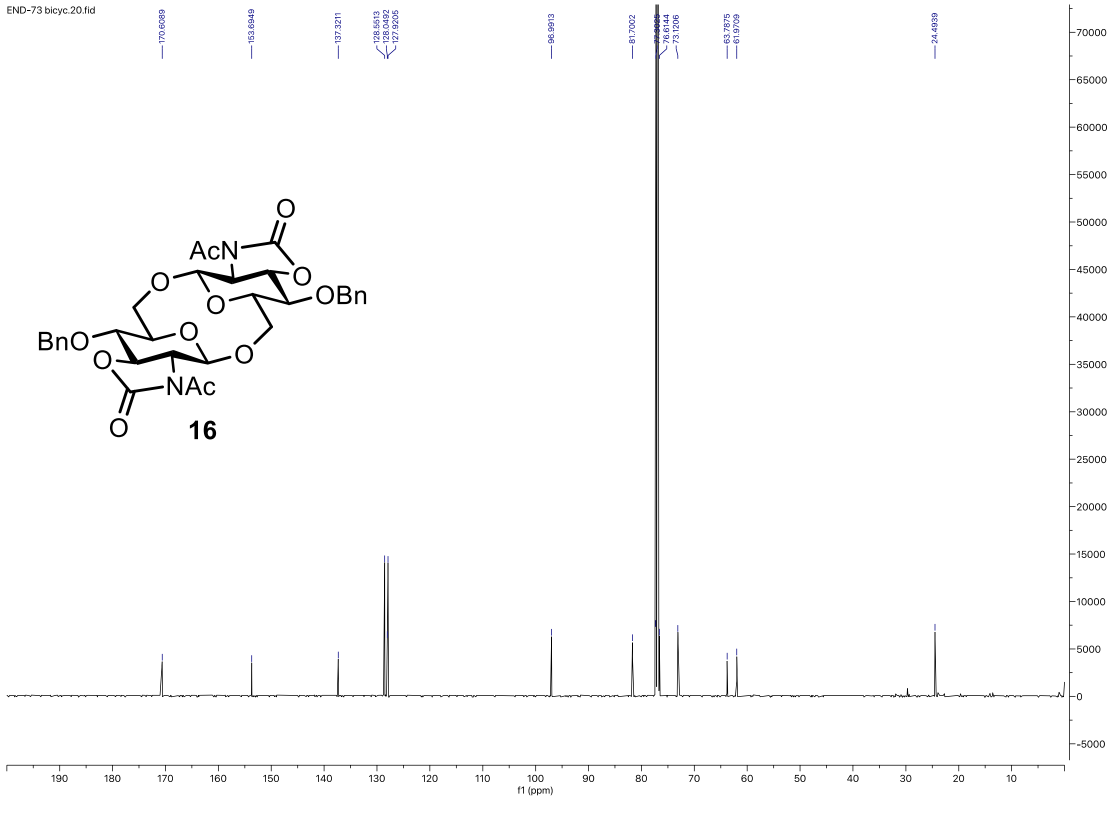

^{1}H, ^{1}H-COSY

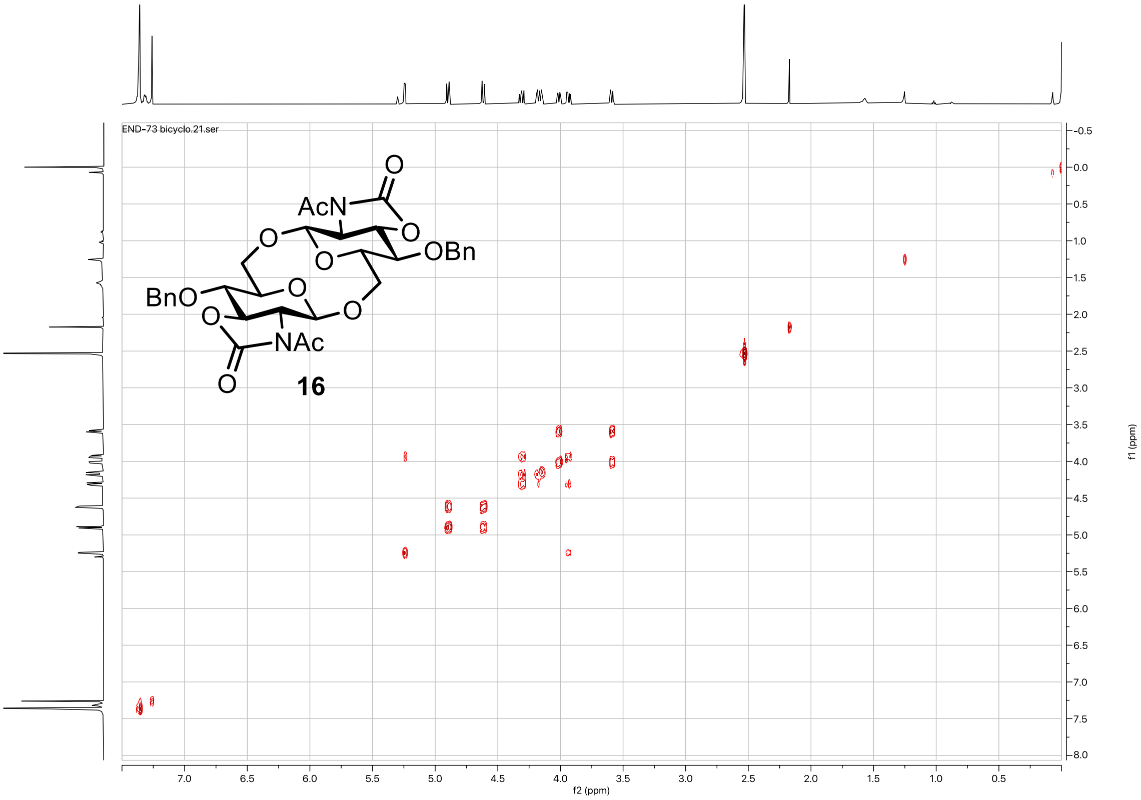

^{13}C NMR

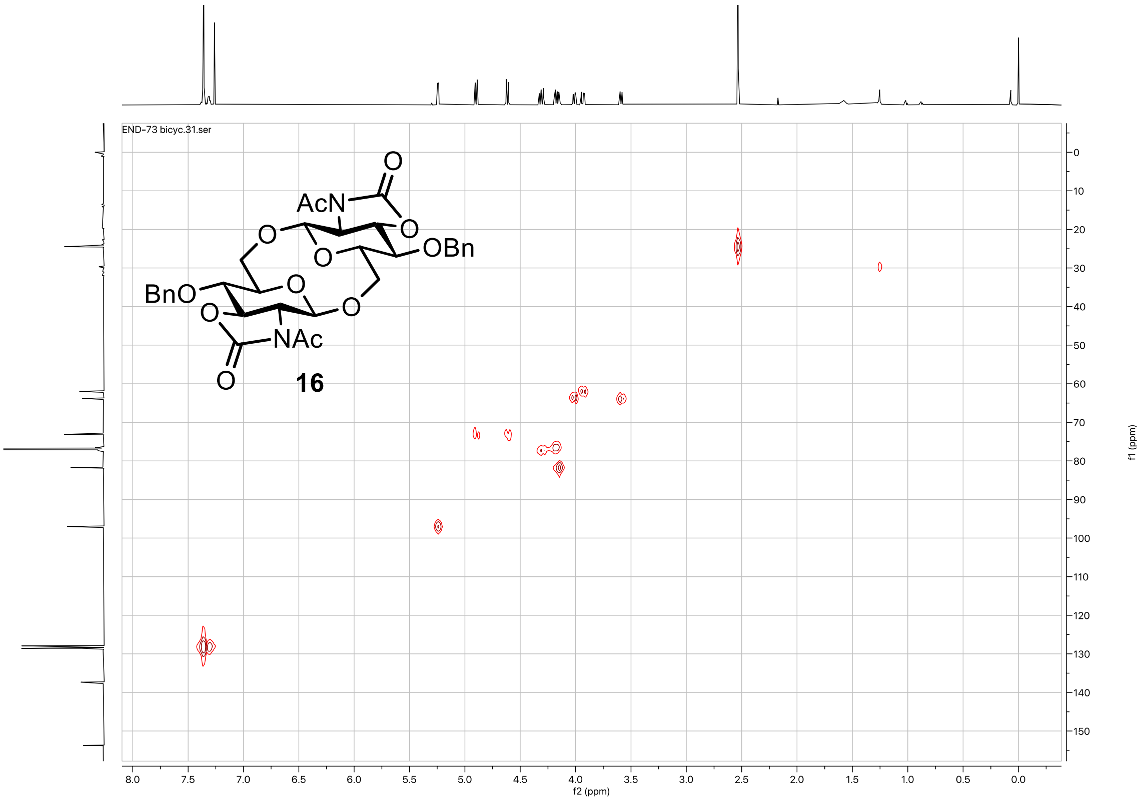

^{1}H NMR

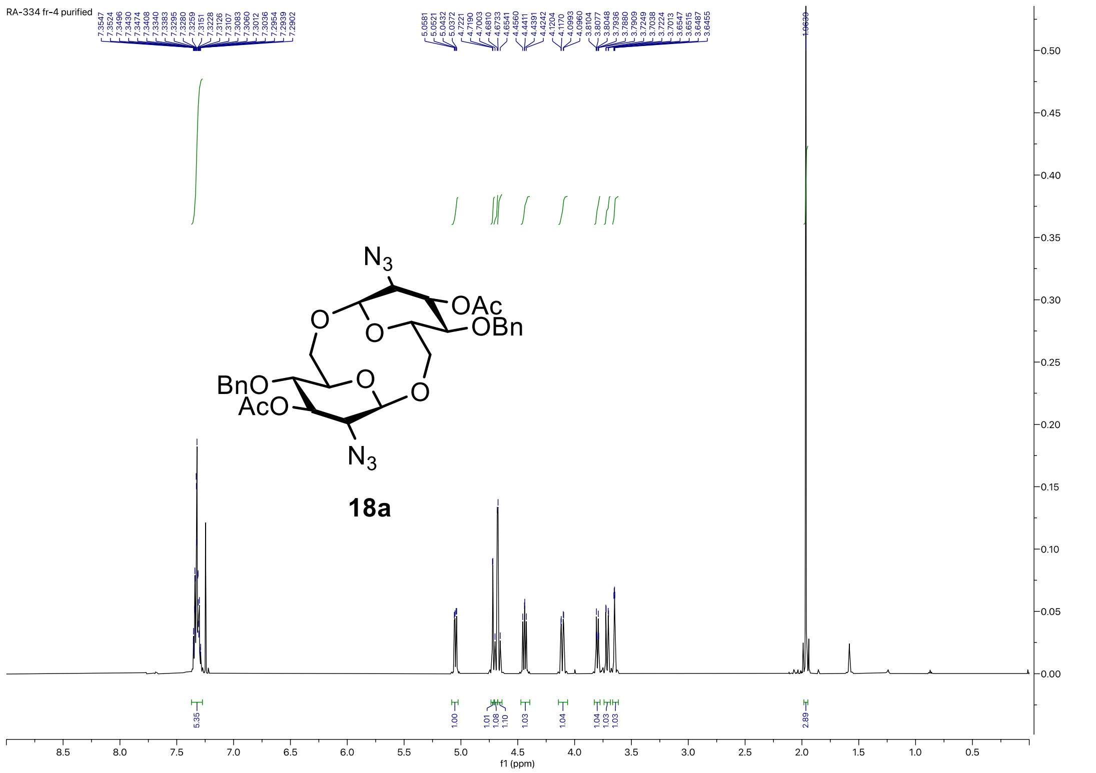

^{13}C NMR

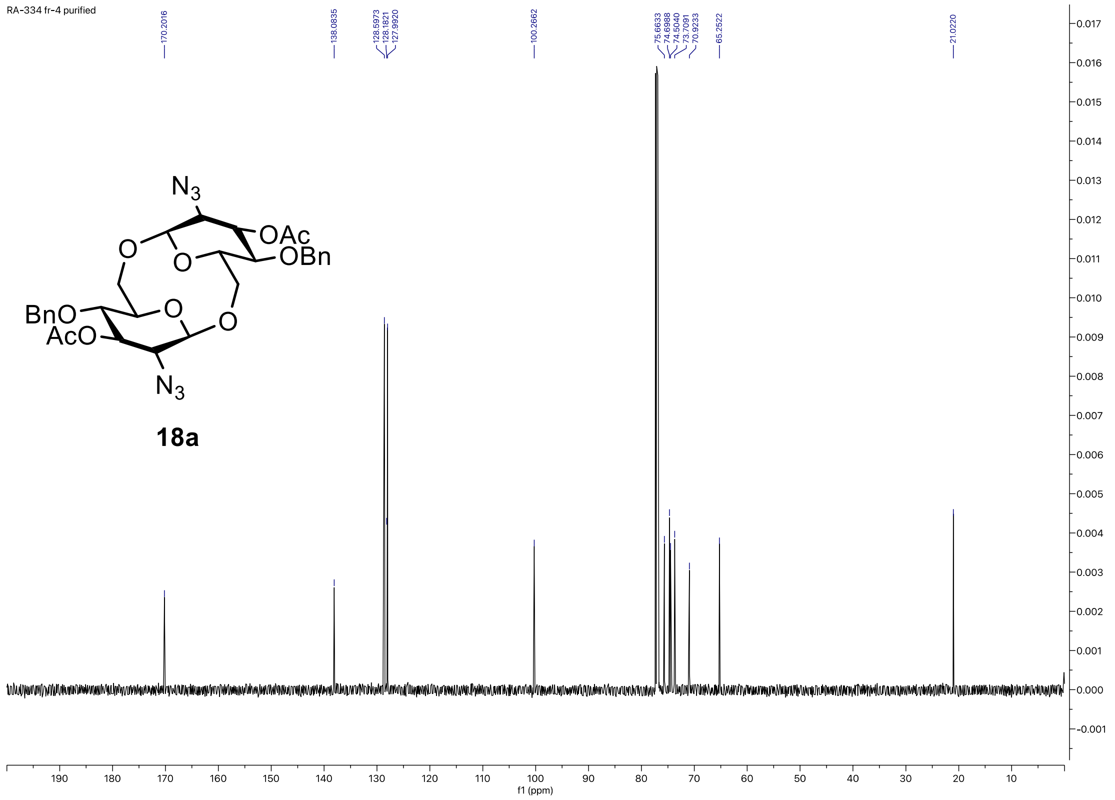

^1H, ^1H-COSY

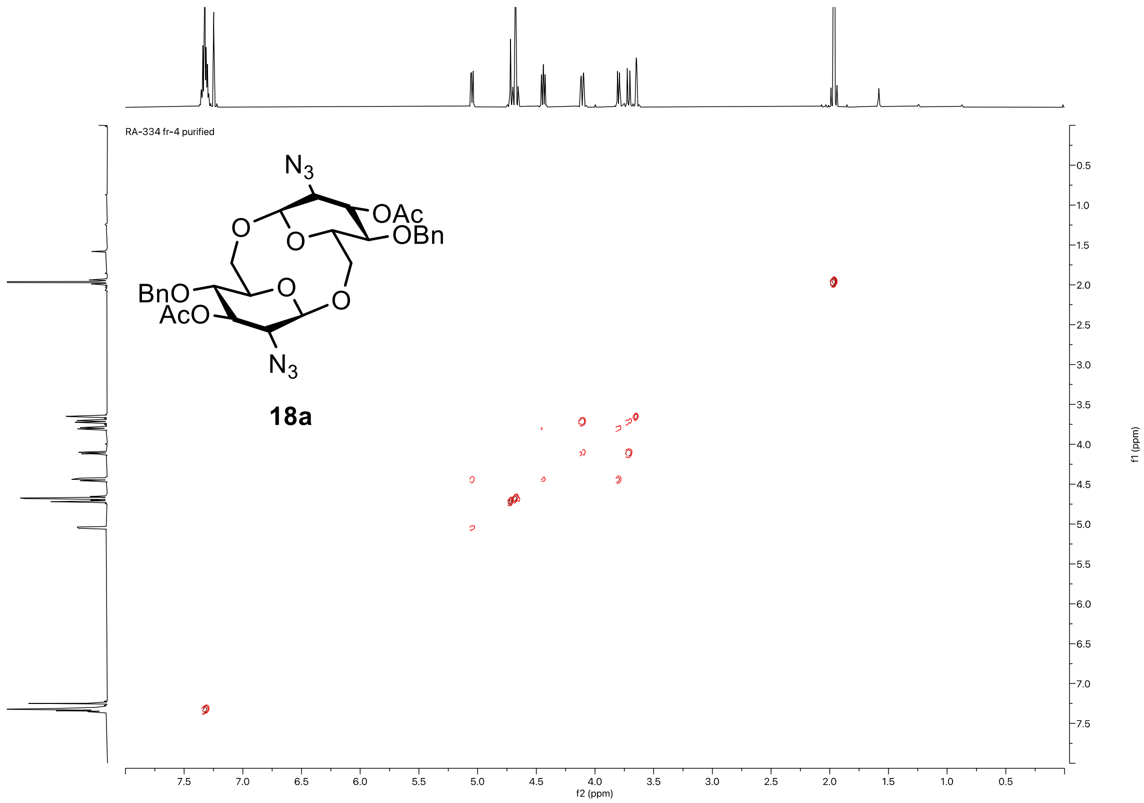

HMQC

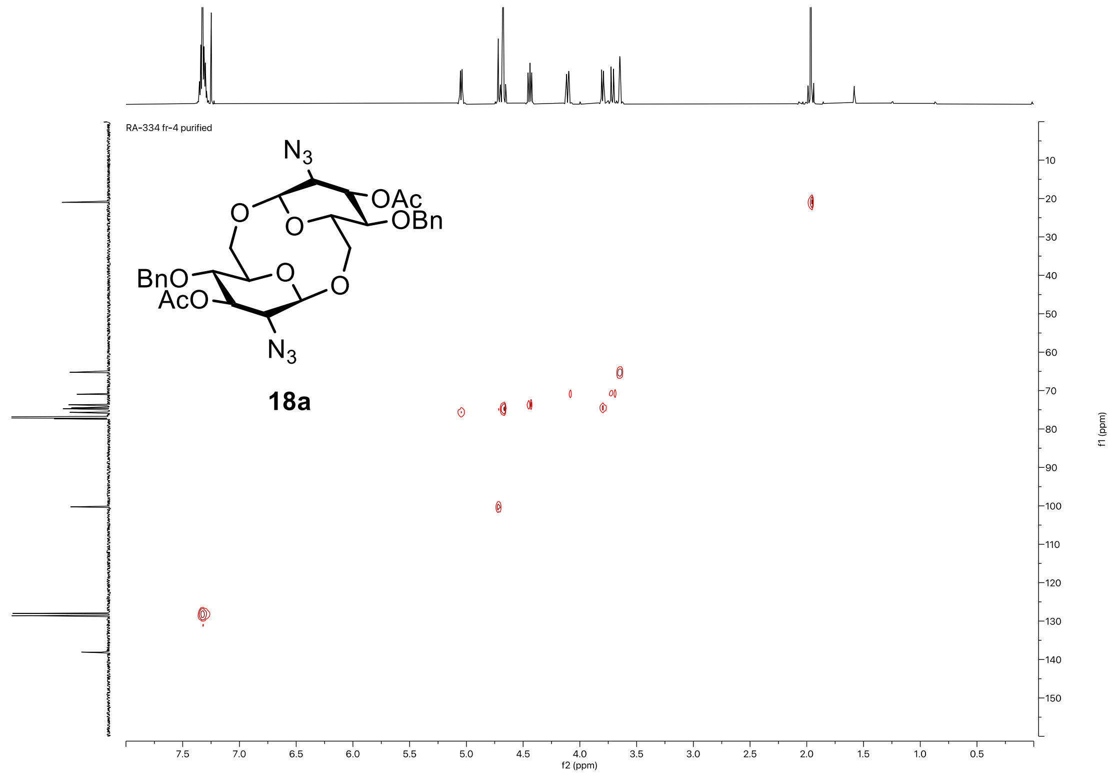

^{1}H NMR

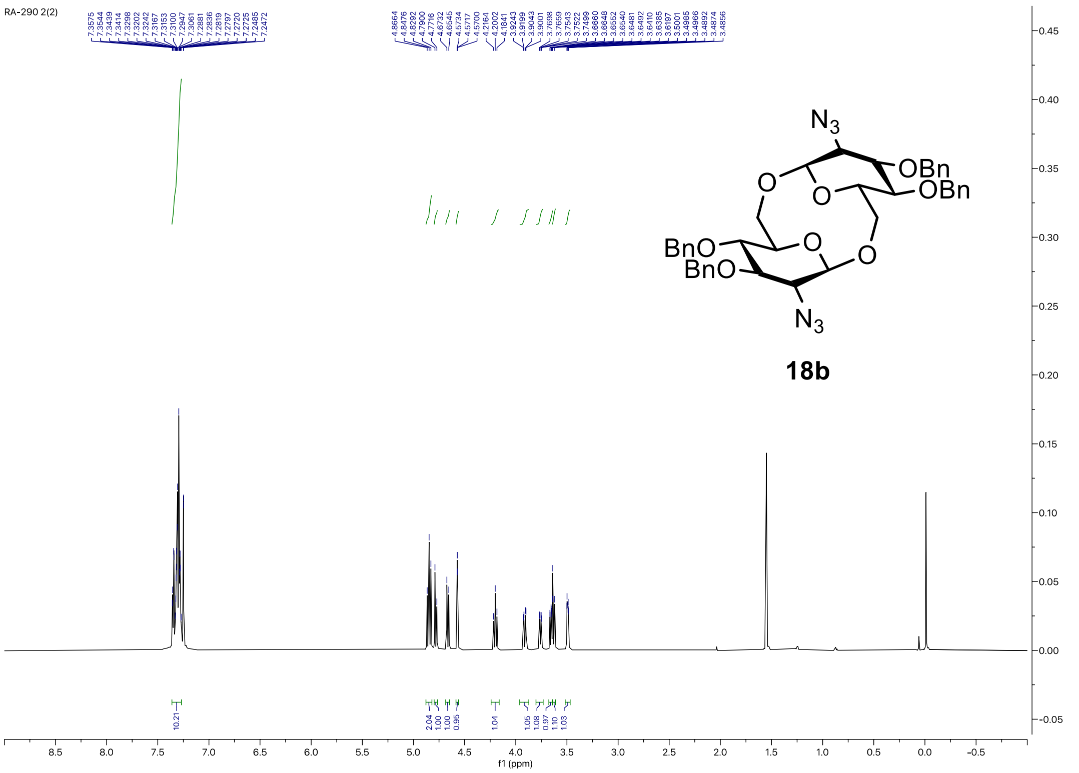

^{13}C NMR

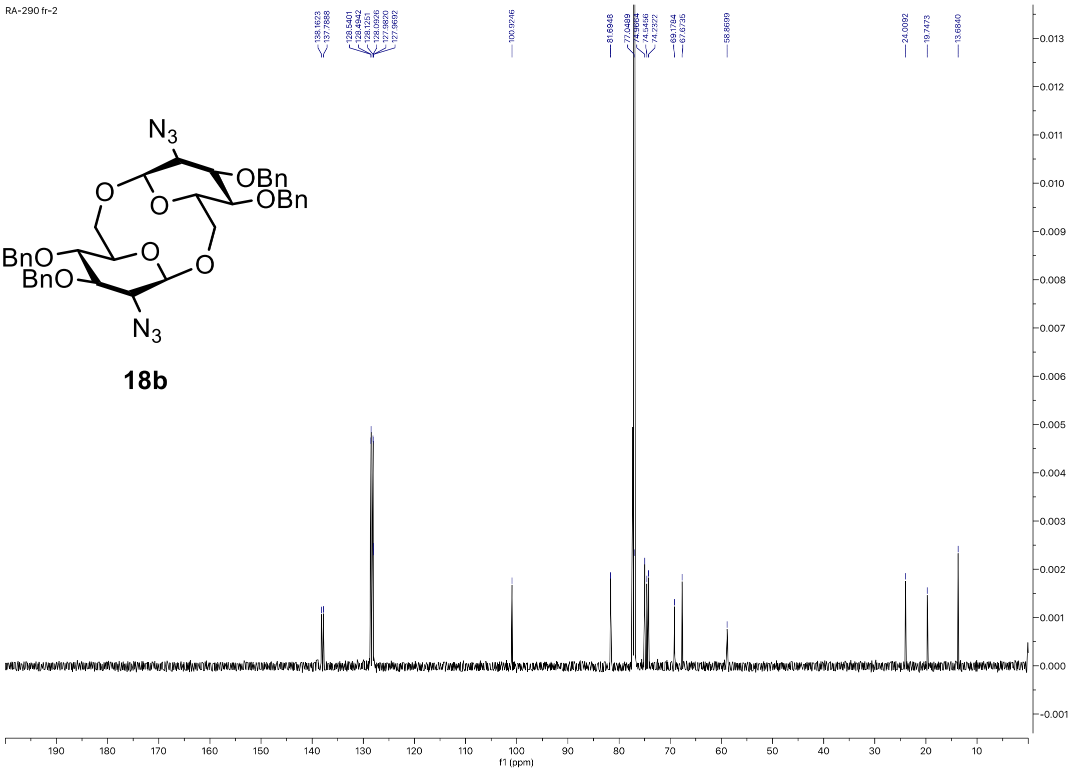

^{1}H, ^{1}H-COSY

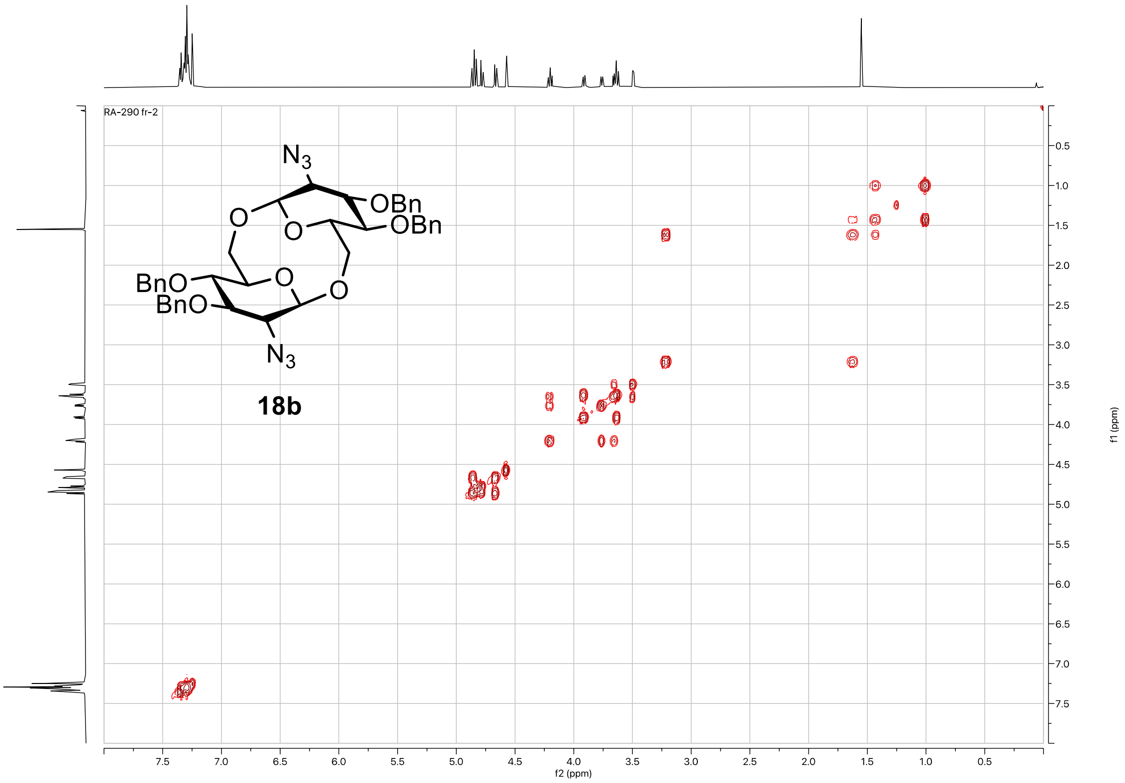

HMQC

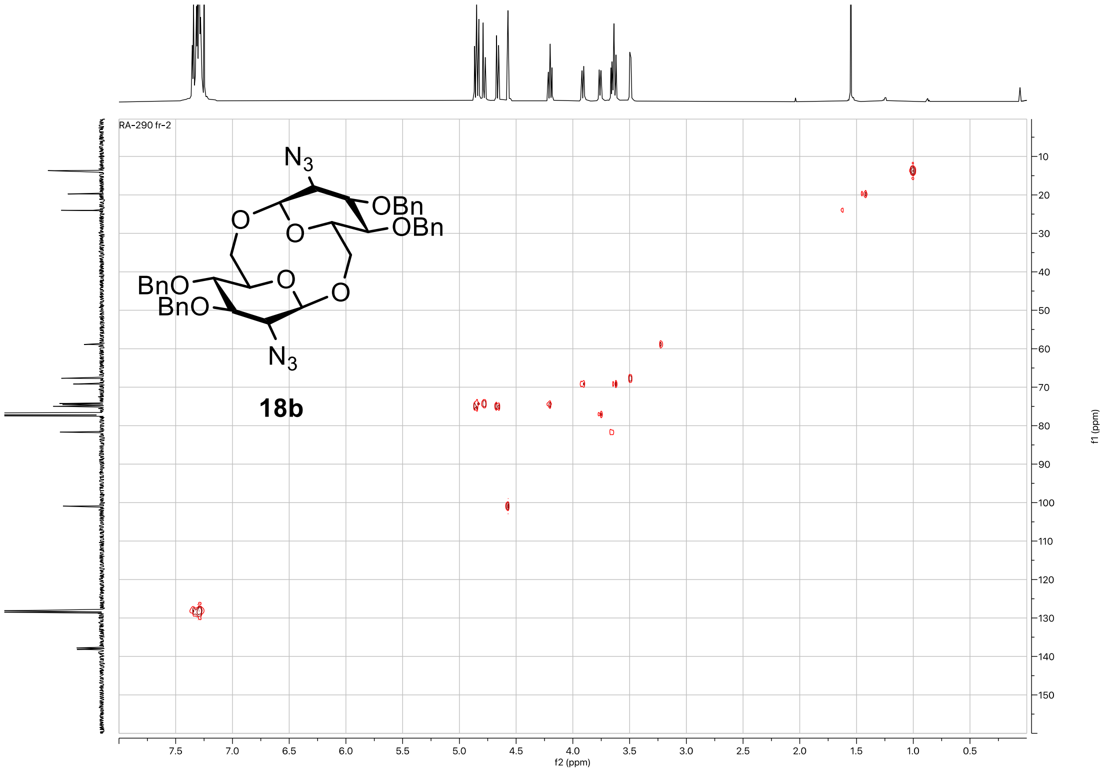

^{1}H NMR

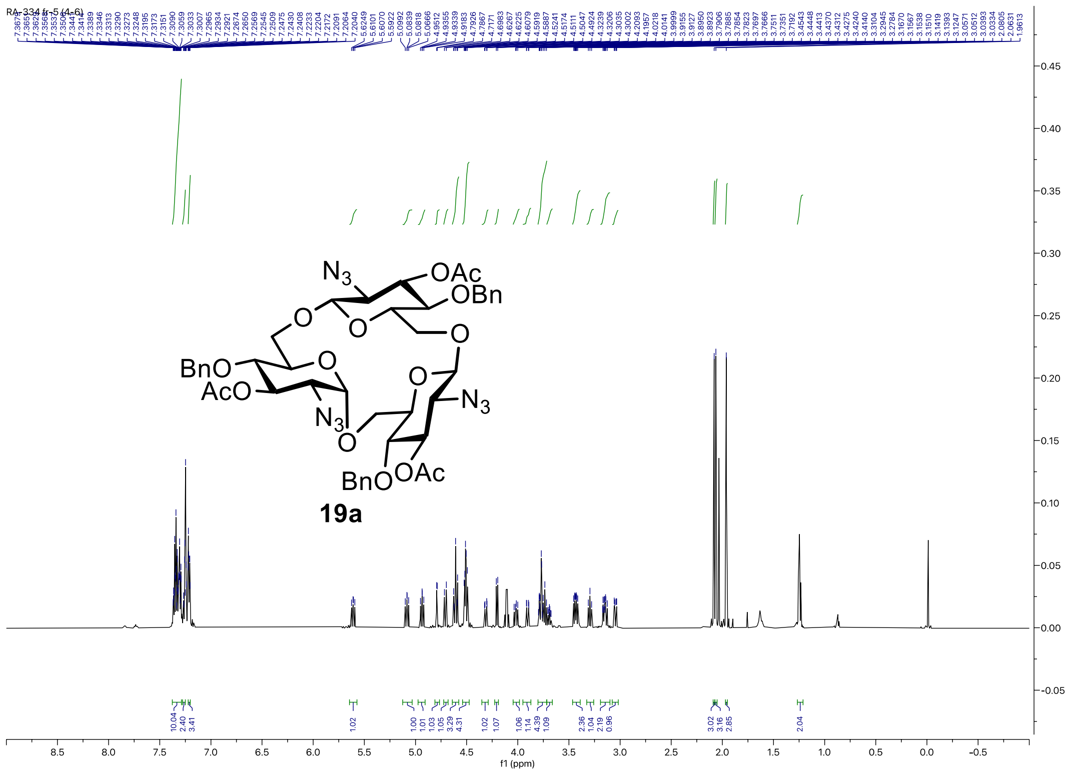

^{13}C NMR

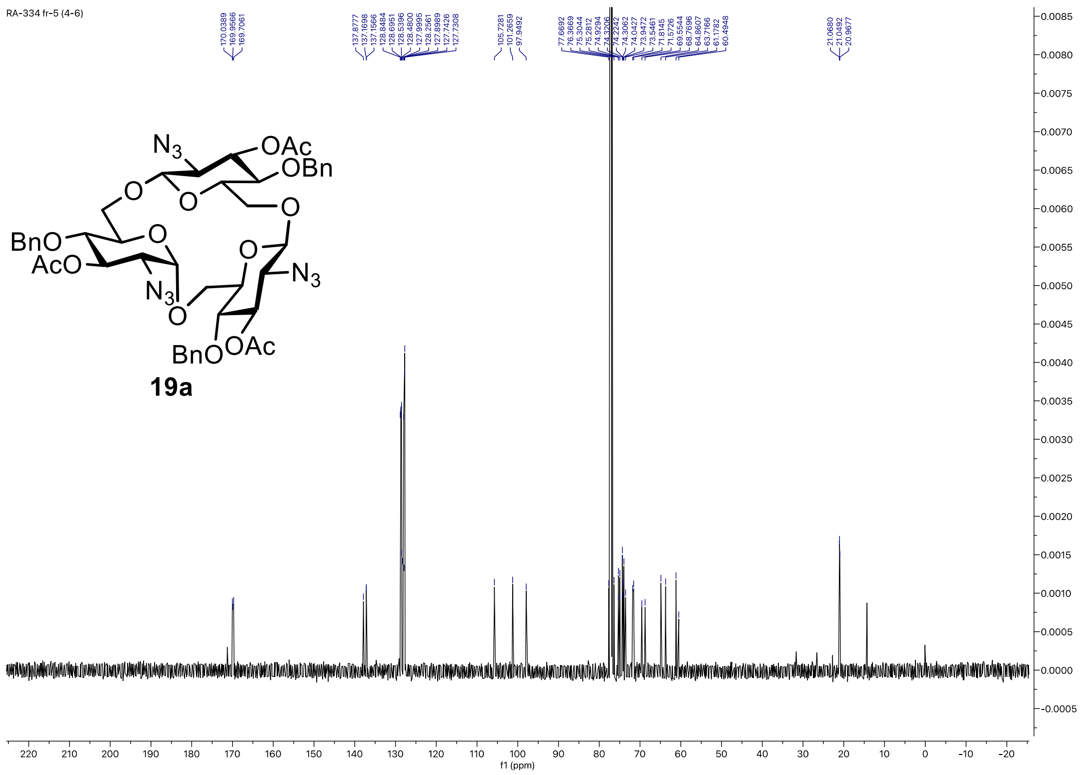

^{1}H, ^{1}H-COSY

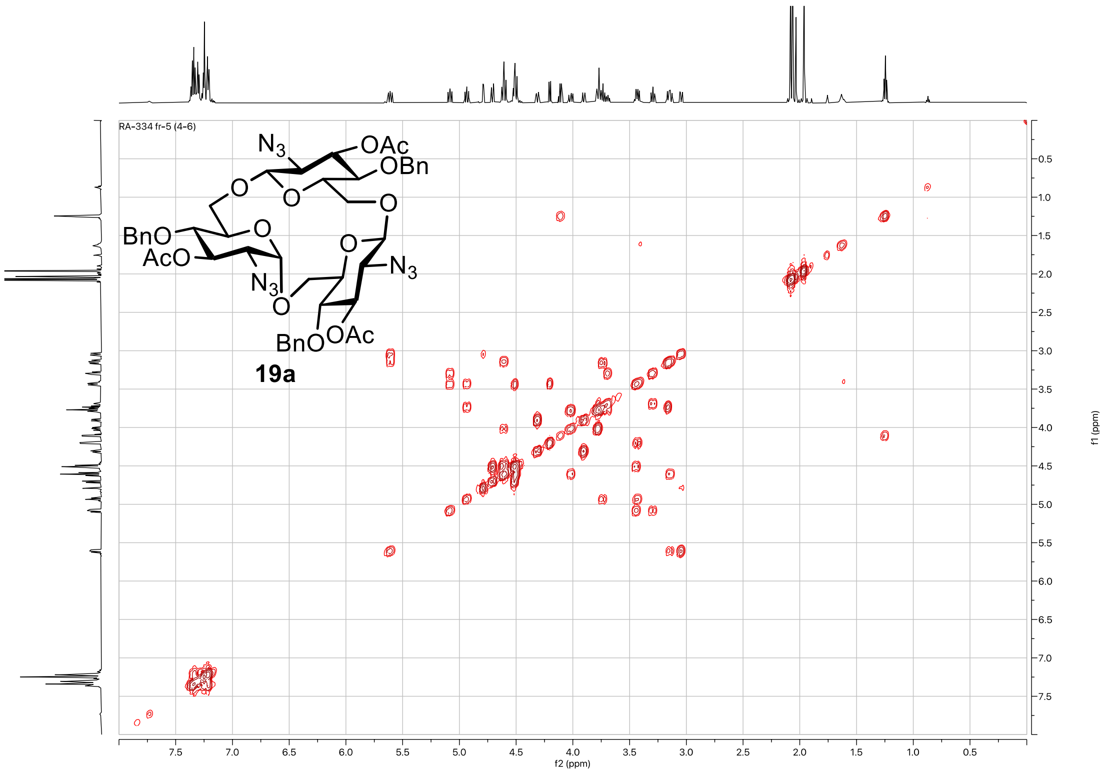

HMQC

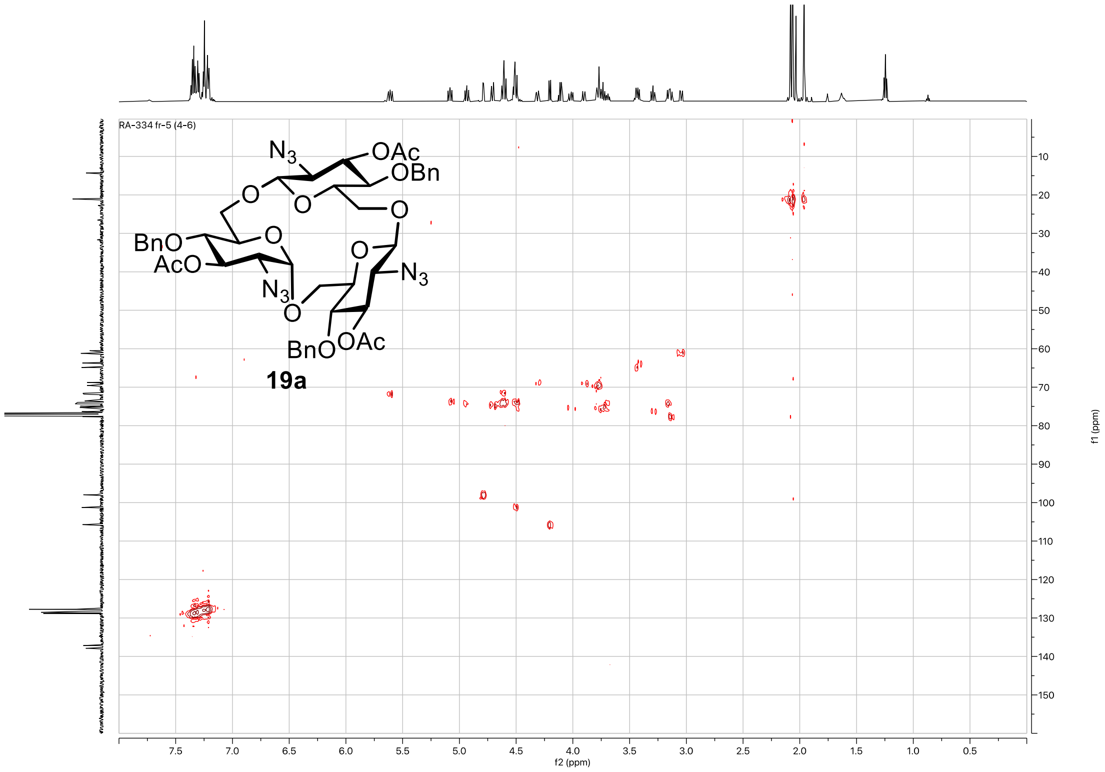

^{1}H NMR

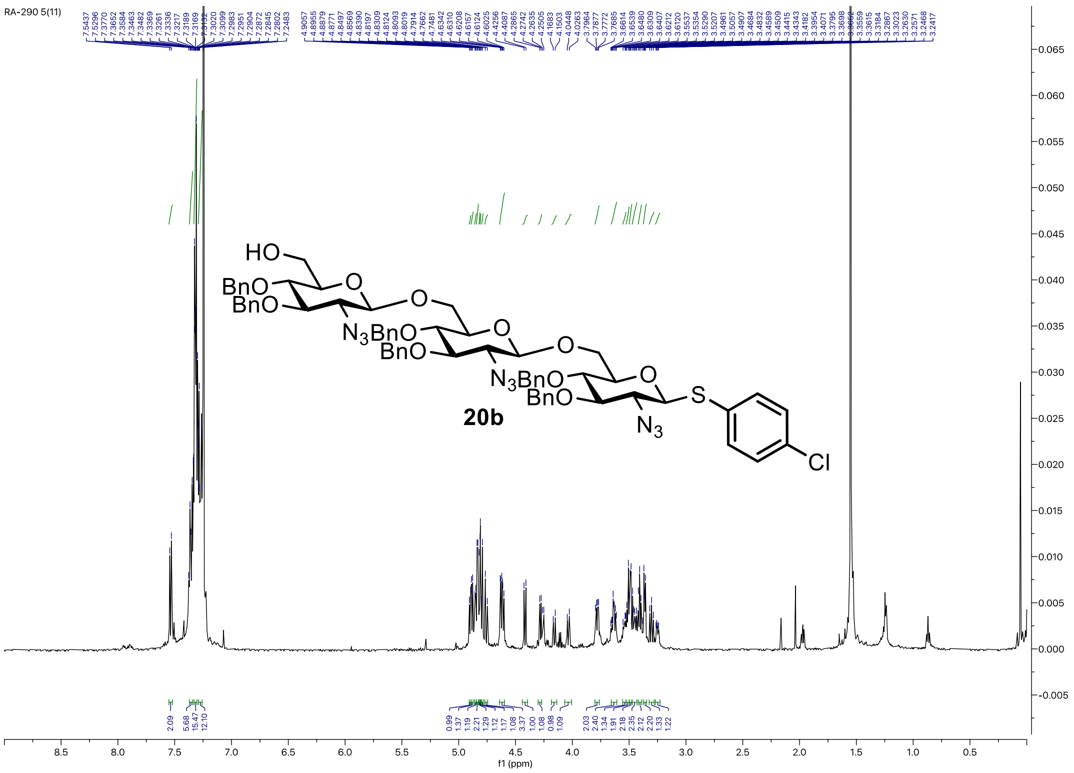

^{13}C NMR

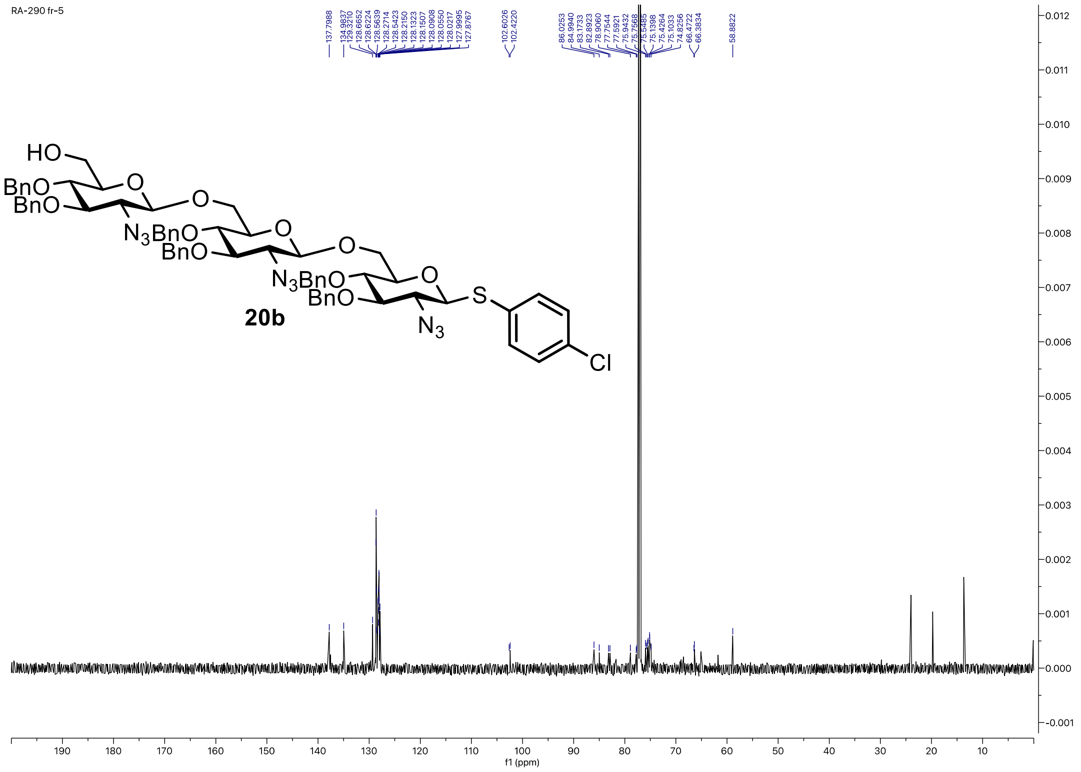

^{1}H, ^{1}H-COSY

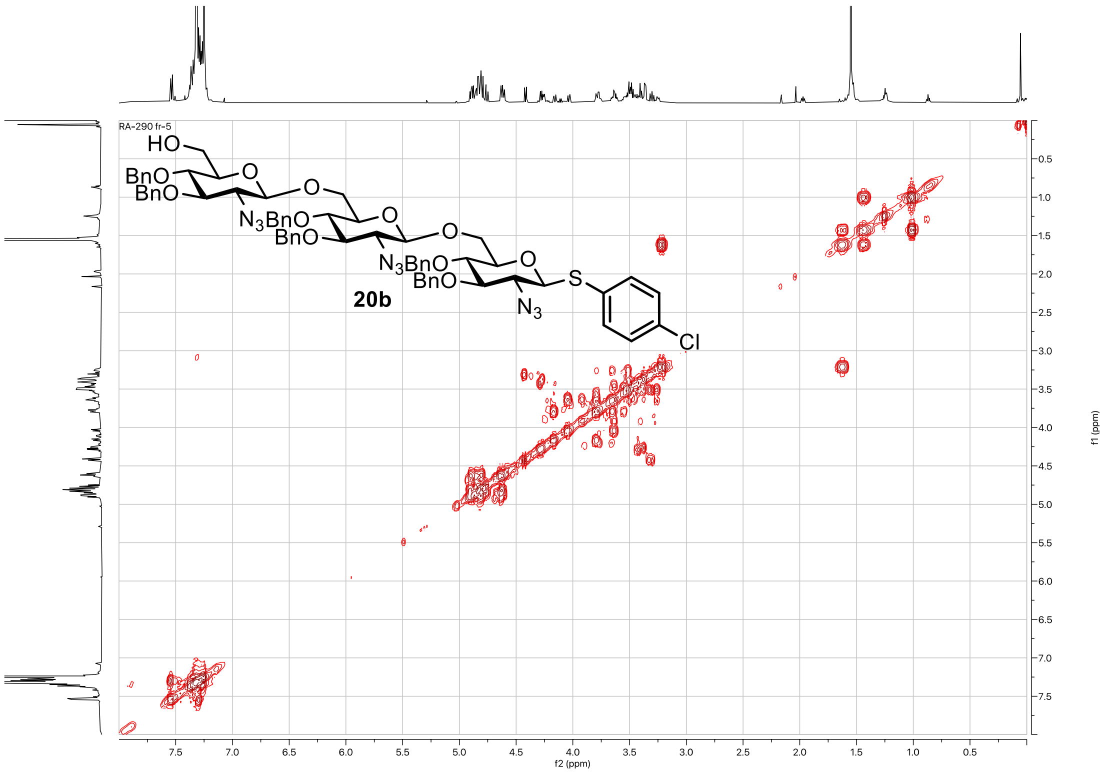

HMQC

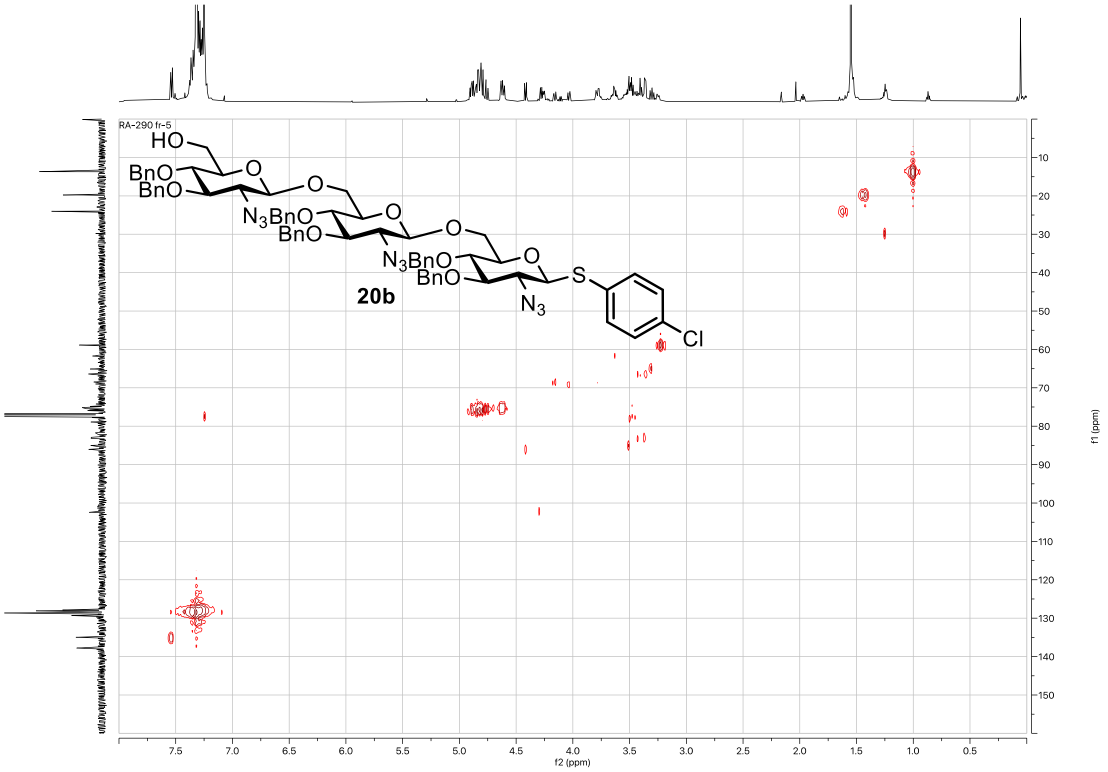

Supplement: File 1 — Synthetic details, DFT calculations, and compound characterization data. [file Beilstein_J_Org_Chem-20-1421-s001.pdf]
